# Unlocking coordination sites of metal–organic frameworks for high-density and accessible copper nanoparticles toward electrochemical nitrate reduction to ammonia†

**DOI:** 10.1039/d4sc07132h

**Published:** 2025-03-06

**Authors:** Cheng-Hui Shen, Yingji Zhao, Ho Ngoc Nam, Liyang Zhu, Quan Manh Phung, Vic Austen, Minjun Kim, Dong Jiang, Xiaoqian Wei, Tokihiko Yokoshima, Chung-Wei Kung, Yusuke Yamauchi

**Affiliations:** a Department of Chemical Engineering, National Cheng Kung University 1 University Road Tainan City Taiwan cwkung@mail.ncku.edu.tw; b Department of Materials Process Engineering, Graduate School of Engineering, Nagoya University Nagoya 464-8603 Japan zhao.yingji.n2@f.mail.nagoya-u.ac.jp y.yamauchi@uq.edu.au; c Department of Chemistry, Graduate School of Science, Nagoya University Nagoya 464-8603 Japan; d Institute of Transformative Bio-Molecules (WPI-ITbM), Nagoya University Nagoya 464-8603 Japan; e Australian Institute for Bioengineering and Nanotechnology (AIBN), The University of Queensland Brisbane Queensland 4072 Australia

## Abstract

Ordered pore engineering of metal–organic framework (MOF)-based catalysts by soft-template strategies can facilitate the mass transfer of reactants during heterogeneous electrocatalysis. Besides, the abundant open coordination sites generated by the removal of surfactants also open up a new avenue for incorporating active moieties within the framework; however, such studies are still limited. Herein, a mesoporous cerium-based MOF, MUiO-66(Ce), is synthesized by introducing a pluronic triblock copolymer as a template, where abundant open coordination sites are found to be present on the hexa-cerium nodes. By providing rich Ce–OH/Ce–OH_2_ sites, plenty of copper moieties are installed on the framework (denoted as Cu-MUiO-66(Ce)). After the *in situ* reduction process, a high density of copper nanoparticles is confined within MUiO-66(Ce), and Cu@MUiO-66(Ce) is thus obtained. With a high loading of active copper sites and efficient diffusion of reactants, the Cu@MUiO-66(Ce)-modified electrode can achieve an ammonia production rate of 1.875 mg h^−1^ mg_catalyst_^−1^ and a faradaic efficiency of 88.7% for nitrate-to-ammonia reduction. Findings here shed light on the importance of pore engineering of MOF-based catalysts for unlocking open coordination sites and facilitating the mass transfer to enhance the electrocatalytic activity.

## Abstract 2

Pore engineering of Ce-MOF creates open sites and mesoporous channels, enabling high copper loading and accessibility for nitrate-to-ammonia conversion, achieving high production rates and faradaic efficiency.

## Graphical abstract

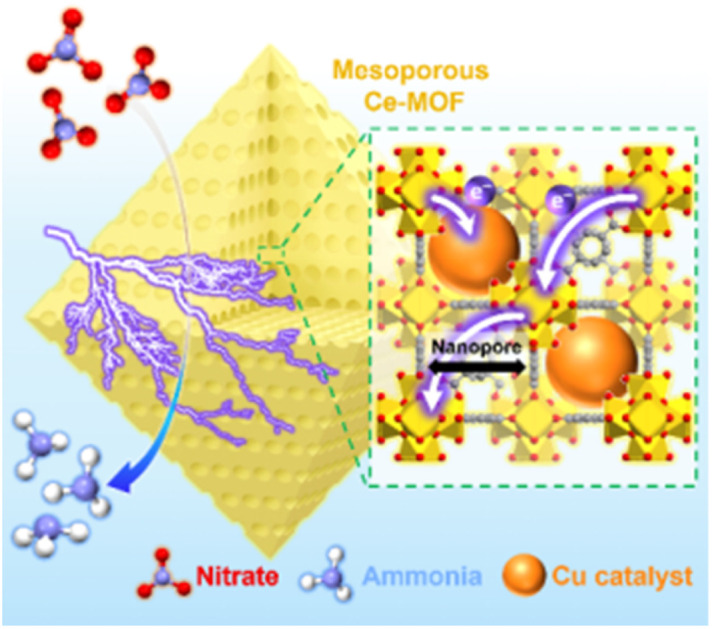

## Introduction

Featuring distinctive characteristics such as ultrahigh specific surface area, ordered porous structure, and tunable chemical functionality, metal–organic frameworks (MOFs) have garnered significant interest across numerous applications.^1–6^ Among various MOFs, frameworks constructed from cerium(iv)-based nodes are particularly appealing due to their exceptional chemical stability in aqueous environments.^7–10^ Moreover, the reversible redox behavior of hexa-cerium nodes provides a hopping pathway for charge transport within the intrinsically insulating frameworks during electrochemical operations,^11,12^ making cerium(iv)-based MOFs (Ce-MOFs) intriguing candidates for electrochemical applications.^13,14^ By performing post-synthetic modifications (PSM),^15–17^ spatially separated active units for targeted reactions can be further introduced within the entire framework structure,^18,19^ which makes Ce-MOFs especially attractive for catalysis.^20,21^ Nevertheless, the intrinsically limited open coordination sites restrict the installation of active units,^22^ and the inherent microporous nature of most MOFs severely hinders the transport of reactants,^23–25^ resulting in poor activity for electrocatalytic reactions.^26^ Recently, pore engineering of MOFs has been regarded as a promising strategy to facilitate mass transfer.^27–29^ By carefully adopting suitable polymeric surfactants as soft templates, the self-assembly of metal ions and organic linkers with surfactants can be performed.^30–32^ After removing the template agents from the hybrids, MOFs embedded with ordered mesoporous arrays can be facilely fabricated.^27,28^ Despite several studies dedicated to elucidating the design of ordered mesopores within frameworks for improving the diffusion of guest molecules, the presence of open coordination sites created by the removal of template agents for PSM has not been reported to date. In previous studies, Gu *et al.* disclosed the assembly process of a Ce-MOF with soft templates, where Ce–oxo clusters were initially coordinated with surfactant molecules before the growth of the framework.^28,33^ After the removal of templates, abundant Ce–OH sites were supposed to be present on cerium clusters exposed to the pore channels.^28^ We thus reason that such chemically robust and redox-active Ce-MOFs with ordered mesopores for facilitating mass transfer as well as providing abundant open coordination sites for the incorporation of specific moieties can further open new avenues for boosting electrocatalytic performances. To the best of our knowledge, the concept of bi-functional mesoporous MOFs with abundant open coordination sites and facile diffusion of reactants for electrocatalysis has not been reported yet.

Among various electrocatalytic reactions, the electrochemical reduction of nitrate (NO_3_^−^) to ammonia (NH_3_) is particularly noteworthy,^34,35^ where the metallic copper with electronic energy levels matching the molecular orbitals of nitrate is regarded as a promising electrocatalyst for suppressing the competitive hydrogen evolution reaction (HER) and achieving higher faradaic efficiency for ammonia production.^36–39^ However, such heterogeneous catalysis takes place at the interface between the catalyst and electrolytes, where the low porosity and limited surface-to-volume ratio of bulk copper-based materials result in low metal utilization.^40,41^ In this regard, an electrocatalyst with high accessibility of active sites is strongly desired to achieve satisfactory catalytic performances. Despite copper-based MOFs being reported to possess high surface area and regular porosity, most of them cannot preserve their crystallinity after long-term exposure to aqueous solutions or during the *in situ* formation of copper nanoparticles.^42,43^ By serving a Ce-MOF as a porous skeleton, copper ions can be decorated on the hexa-cerium nodes,^21,44,45^ and spatially separated copper particles confined within the framework can be further obtained after the electrochemical treatment.^46^ Additionally, the cerium(iv)-based support has also been found to facilitate the conversion of the intermediate on the metallic copper surface and achieve superior selectivity for ammonia production compared to zirconium(iv)– or graphene-based supports in our previous study.^45^ As a result, a state-of-the-art electrocatalyst with a high loading of active copper units and facile mass transfer of reactants for nitrate-to-ammonia reduction is anticipated through the *in situ* formation of copper nanoparticles within a mesoporous Ce-MOF. However, either the investigation of open coordination sites generated by the removal of soft templates within mesoporous MOFs for post-synthetic modifications or the introduction of copper nanoparticles within mesoporous Ce-MOFs for efficient nitrate-to-ammonia catalysts has not been reported in any literature to date.

**Fig. 1:**
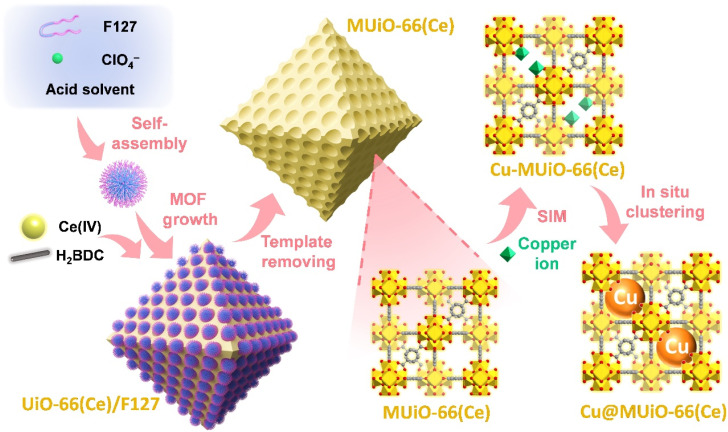
Schematic illustration of the synthetic process for preparing MUiO-66(Ce), Cu-MUiO-66(Ce), and Cu@MUiO-66(Ce). Yellow, red, and gray atoms shown in crystal structures represent Ce, O, and C, respectively. Hydrogen atoms are not shown for clarity.

Herein, a pluronic poly(ethylene oxide)–poly(propylene oxide)–poly(ethylene oxide) (PEO–PPO–PEO) surfactant, F127 (PEO_106_PPO_70_PEO_106_), is served as a soft template to induce the oriented crystallization of MOF,^47–49^ and crystals of a Ce-MOF, UiO-66(Ce), with ordered mesopores (denoted as MUiO-66(Ce)) are then synthesized *via* a hydrothermal method.^47^ The immobilization of copper ions on the hexa-cerium nodes of MUiO-66(Ce) is further conducted by a solvothermal deposition in MOFs (SIM) method to obtain “Cu-MUiO-66(Ce)”.^44^ Subsequently, copper nanoparticles are *in situ* constructed within the MUiO-66(Ce) skeleton through an electroreduction method,^46^ and “Cu@MUiO-66(Ce)” is thus prepared (Fig. 1). For comparison, samples without mesopores, UiO-66(Ce) and Cu-UiO-66(Ce), are also fabricated, and the Cu@UiO-66(Ce)-modified electrode is further prepared through the electrochemical treatment. Since UiO-66(Ce) has been reported to decompose under alkaline electrolytes,^8^ the ammonia production rates and faradaic efficiencies of these modified electrodes, particularly for the practical application using wastewater with low nitrate concentrations, are then investigated in a neutral electrolyte.

## Results and discussion

### Material characterizations

**Fig. 2:**
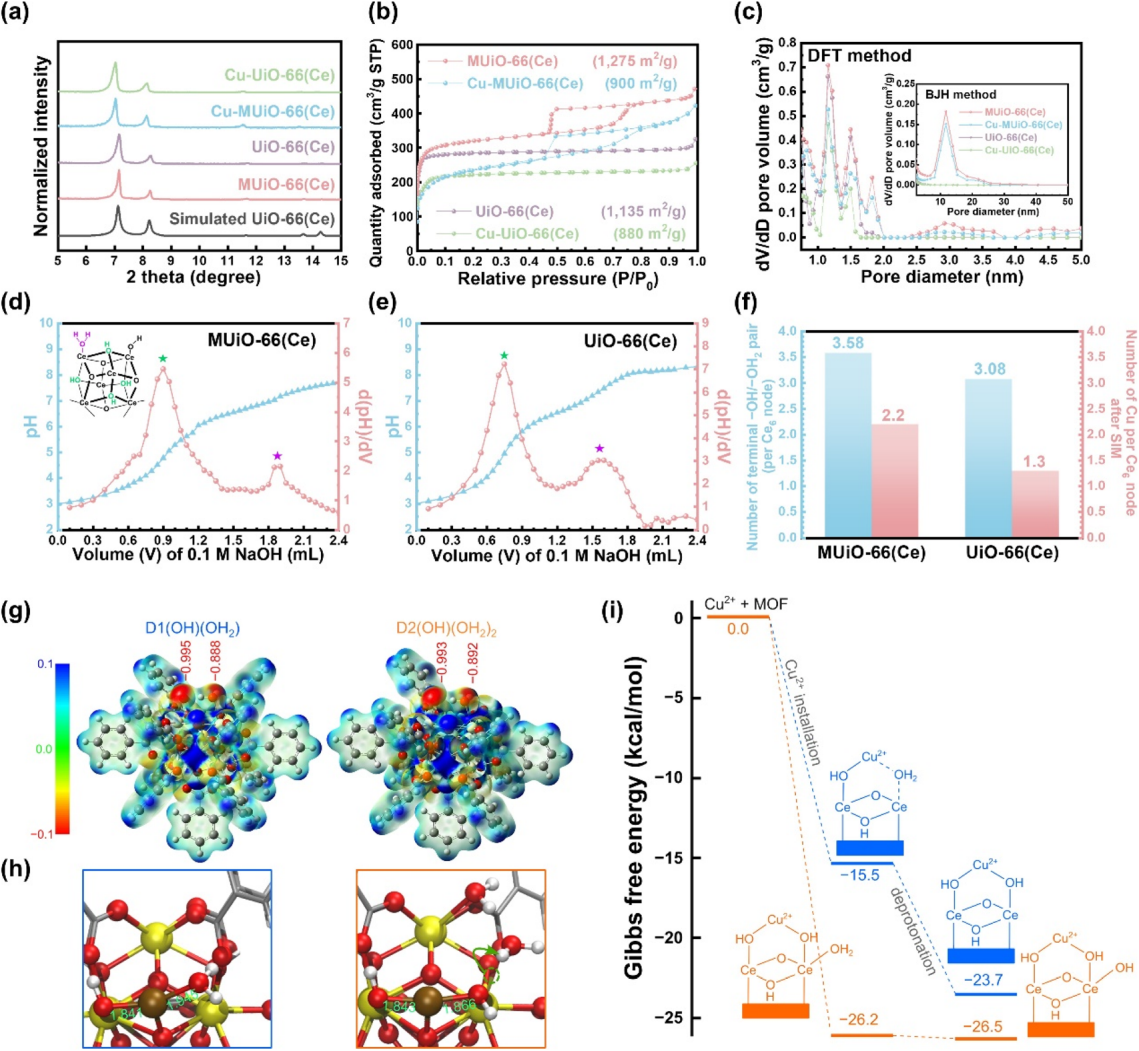
(a) XRD patterns of MUiO-66(Ce), UiO-66(Ce), Cu-MUiO-66(Ce), and Cu-UiO-66(Ce) in the low-angle range. The simulated pattern of UiO-66(Ce) is also shown. (b) Nitrogen adsorption–desorption isotherms of MUiO-66(Ce), UiO-66(Ce), Cu-MUiO-66(Ce), and Cu-UiO-66(Ce). The BET surface area of each sample is also shown. (c) Pore size distributions of MUiO-66(Ce), UiO-66(Ce), Cu-MUiO-66(Ce), and Cu-UiO-66(Ce) calculated from isotherms shown in (b) by using the DFT method and the BJH method (inset figure). Titration curves and their first-derivative curves for (d) MUiO-66(Ce) and (e) UiO-66(Ce). The hexa-cerium cluster of defective UiO-66(Ce) with the bridging μ_3_–OH and the terminal –OH/–OH_2_ pair is also shown in the inset of (d). (f) Plots of the number of terminal –OH/–OH_2_ pair per hexa-cerium node before SIM, and the number of copper anchored on each hexa-cerium node after SIM for MUiO-66(Ce) and UiO-66(Ce). (g) The electrostatic potential surfaces of two clusters, D1(OH)(OH_2_) and D2[(OH)(OH_2_)]_2_. The red numbers denote the DDEC6 atomic charges of oxygen atoms. (h) The structure of the Cu^2+^–D1(OH)(OH_2_) and Cu^2+^–D2[(OH)(OH_2_)]_2_ complexes. Yellow, brown, red, silver, and white balls represent Ce, Cu, O, C, and H atoms, respectively. The green numbers represent the Cu–O bond distances (in Å). (i) Gibbs free energy (in kcal mol^−1^) for the installation of Cu^2+^ into D1(OH)(OH_2_) and D2[(OH)(OH_2_)]_2_, one –OH group on the other side of the Cu^2+^ ion for charge balancing is not shown for clarity.

Experimental procedures for the hydrothermal synthesis of MUiO-66(Ce) and UiO-66(Ce) as well as the use of copper(ii) acetate monohydrate as the precursor for SIM to synthesize Cu-MUiO-66(Ce) and Cu-UiO-66(Ce) can be found in ESI.† Powder X-ray diffraction (PXRD) patterns of MUiO-66(Ce), UiO-66(Ce), Cu-MUiO-66(Ce), and Cu-UiO-66(Ce) in a wide-angle range are compared with the simulated one (Fig. S1†). This consistency indicates that the highly crystalline UiO-66(Ce) structure is formed during the growth of MOFs, and the crystallinities can be well preserved after the incorporation of copper sites. However, an obvious shift of the first diffraction peak to a smaller angle is observed in the low-angle range of the patterns after the SIM process, suggesting that the incorporation of copper sites within the frameworks slightly expands the lattice size of UiO-66(Ce) structure (Fig. 2a).

An octahedral crystal with a size of around 700 nm is observed in the scanning electron microscopy (SEM) image of MUiO-66(Ce), which reveals a large number of mesopores uniformly distributed on the surface of a MUiO-66(Ce) particle (Fig. S2a†). On the other hand, UiO-66(Ce) shows a smaller crystal size of around 240 nm in the SEM image, and there are not any visible pores on the surface of the particle (Fig. S2b†). After the installation of copper sites, similar morphologies can still be observed in SEM images of Cu-MUiO-66(Ce) and Cu-UiO-66(Ce) (Fig. S2c and d†). Low-magnification SEM images shown in Fig. S3† also indicate the morphological uniformity of these materials. As shown in Fig. S4a,† a transmission electron microscopy (TEM) image reveals the mesoporous structure of MUiO-66(Ce), and a high-resolution TEM (HRTEM) image of MUiO-66(Ce) further confirms the presence of spherical mesopores distributed within the particle (Fig. S4b†). Moreover, a selected-area electron diffraction (SAED) pattern collected from an MUiO-66(Ce) crystal exhibits the single-crystalline nature of the synthesized MUiO-66(Ce) (Fig. S5†). A TEM image of Cu-MUiO-66(Ce) demonstrates that the mesoporous structure still remains after the incorporation of copper sites (Fig. S6a†), and no obvious particles can be observed in the HRTEM image of Cu-MUiO-66(Ce) (Fig. S6b†), suggesting that there is no metallic copper present within the framework. The TEM and HRTEM images of UiO-66(Ce) reveal its non-mesoporous structure (Fig. S7a and b†), and there is no formation of particles after the SIM process (Fig. S7c and d†).

Nitrogen adsorption–desorption isotherms of MUiO-66(Ce), UiO-66(Ce), Cu-MUiO-66(Ce), and Cu-UiO-66(Ce) were recorded to investigate the porosity of each material (Fig. 2b). MUiO-66(Ce) and Cu-MUiO-66(Ce) show a combination of type I and type IV isotherms, where the vertically rising sorption at low relative pressure implies inherent micropores of the framework, while the hysteresis loop at relative pressures from 0.45 to 0.8 hints the presence of cage-type mesopores.^28^ On the other hand, only microporous properties can be observed in the isotherms of UiO-66(Ce) and Cu-UiO-66(Ce). The decreases in Brunauer–Emmett–Teller (BET) surface area are observed after the installation of copper sites, with the reductions from 1275 m^2^ g^−1^ to 900 m^2^ g^−1^ for MUiO-66(Ce) and from 1135 m^2^ g^−1^ to 880 m^2^ g^−1^ for UiO-66(Ce), respectively. Pore size distributions were further calculated by both the density functional theory (DFT) model and the Barrett–Joyner–Halenda (BJH) method (Fig. 2c). It can be observed that both MUiO-66(Ce) and UiO-66(Ce) possess the main micropore size around 1.1 nm along with additional pores of the size ranging from 1.3 to 2.0 nm caused by the defects,^26^ whereas only MUiO-66(Ce) has mesopores centered at a size of 11.7 nm, which corresponds to the pore size observed in the HRTEM image (Fig. S4b†). After the SIM process, their pore volumes are slightly decreased but pore sizes remain the same, suggesting that the copper sites are installed in the framework without clogging the majority of the porosities. Thereafter, periodically ordered mesopores in MUiO-66(Ce) were confirmed by a small-angle X-ray scattering (SAXS) measurement, and a pore-to-pore spacing of 14.8 nm is determined from the peak with a *q*-value of 0.42 nm^−1^ in the pattern (Fig. S8†).^28,50^

For the incorporation of copper ions, the terminal –OH/–OH_2_ pairs present on the hexa-cerium nodes of MUiO-66(Ce) and UiO-66(Ce) is required.^21^ The acid–base titrations were thus carried out to quantify the amount of terminal –OH/–OH_2_ pairs, and the resulting curves along with their first derivatives for MUiO-66(Ce) and UiO-66(Ce) are shown in Fig. 2d and e, respectively. Two distinct equivalence points with p*K*a values of around 3.44 and 6.54 represent the bridging μ_3_–OH and the first proton of the terminal –OH_2_ group on the clusters, respectively.^51–53^ The amounts of defective sites were thereafter calculated, and the structural formulas Ce_6_O_4_(OH)_4_(BDC)_4.21_[(OH)(OH_2_)]_3.58_ and Ce_6_O_4_(OH)_4_(BDC)_4.46_[(OH)(OH_2_)]_3.08_ for MUiO-66(Ce) and UiO-66(Ce), respectively, were determined; see Table S1 and detailed discussions in the ESI.† It is found that a greater number of terminal –OH/–OH_2_ pairs are present on MUiO-66(Ce), which may result from either the defective sites caused by missing linkers or the naked cerium clusters that are initially coordinated to F127 before washing. Findings here suggest that MUiO-66(Ce) can provide a higher amount of open coordination sites for anchoring active moieties through the post-synthetic modification compared to UiO-66(Ce). As a result, inductively coupled plasma-atomic emission spectrometry (ICP-AES) measurements for copper-decorated samples were performed, and the average copper loadings on each hexa-cerium node of Cu-MUiO-66(Ce) and Cu-UiO-66(Ce) are 2.2 and 1.3, respectively; the results of acid–base titrations and ICP-AES measurements are concluded in Fig. 2f. Although the amount of terminal –OH/–OH_2_ pairs on the nodes of MUiO-66(Ce) are 1.16 times higher than that of UiO-66(Ce), the average copper loading on the nodes of Cu-MUiO-66(Ce) is even higher, with a ratio of 1.69 compared to Cu-UiO-66(Ce). Findings suggest not only more open coordination sites but also other factors present in MUiO-66(Ce) allowing the installation of more Cu ions. As a result, density functional theory (DFT) calculations are utilized to get insight into the SIM process. Since Cu^2+^ initially interacts with hexa-cerium nodes *via* electrostatic interaction, we analyzed the electrostatic potential surfaces of two simplified model clusters: D1(OH)(OH_2_) with one terminal –OH/–OH_2_ pair and D2[(OH)(OH_2_)]_2_ with two pairs. The cluster, consisting of a [Ce_6_O_4_(OH)_4_] node and benzoate groups (substituting BDC linkers), is shown in Fig. S9.† For the latter case, we considered twelve configurations (Fig. S10†) and found that they are similar in energy (within 5 kcal mol^−1^, see Table S2†). Therefore, we focused on a representative one (Fig. 2g and Table S3†), in which one –OH/–OH_2_ pair could have an impact on the other pair. In all cases, we also reported the DDEC6 atomic charge of the oxygen atoms in –OH and –OH_2_. As expected, the electrostatic potential surfaces reveal electron-rich regions near the –OH/–OH_2_ pair, which is important for the installation of Cu^2+^. The slightly more negative electrostatic potential and oxygen atomic charge on –OH compared to –OH_2_ suggest a stronger interaction between Cu^2+^ and –OH, which is consistent with the shorter Cu–OH distance (1.84 Å) compared to Cu–OH_2_ (1.95 Å) (Fig. 2h). The electrostatic potential surface and the charges in D2[(OH)(OH_2_)]_2_ indicate that an –OH/–OH_2_ pair would have a minor impact on its neighboring pairs. Based solely on the electrostatic potential surfaces, one can conclude that the installations of Cu^2+^ on D1(OH)(OH_2_) and D2[(OH)(OH_2_)]_2_ are very similar. However, we found that the Gibbs free energy for Cu^2+^ installation on D2[(OH)(OH_2_)]_2_ (−24.4 kcal mol^−1^) is significantly more negative than D1(OH)(OH_2_) (−13.6 kcal mol^−1^) (Fig. 2i). This difference of more than 10 kcal mol^−1^ arises from the known flexible dynamics of –OH_2_ in UiO-66.^54^ Apparently, as Cu^2+^ approaches an –OH/–OH_2_ pair in D2[(OH)(OH_2_)]_2_, it simultaneously facilitates a proton transfer from a –OH_2_ to a nearby –OH. This creates a more favorable binding scenario where Cu^2+^ now interacts with two –OH centers of D2[(OH)(OH_2_)]_2_. Our results suggest that, with a higher loading of –OH/–OH_2_ pairs in MUiO-66(Ce), *i.e.*, with a more flexible proton transfer process, the incorporation of Cu^2+^ would be more favorable. Following the Cu^2+^ installation, the complexes can proceed with the deprotonation step.

The electrical conductivities of MUiO-66(Ce), UiO-66(Ce), Cu-MUiO-66(Ce) and Cu-UiO-66(Ce) were measured using a two-probe method; a similar approach has been reported in our previous studies for other MOF-based materials.^55,56^ As demonstrated in Fig. S11 and Table S4,† all the MOFs possess bulk electrical conductivities lower than 10^−12^ S cm^−1^. The results reveal that both copper-decorated Ce-MOFs are intrinsically insulating for electrons in the form of dry powder, and charge transport *via* the redox-hopping pathway must occur in the presence of applied potential and counter ions for the *in situ* formation of metallic copper within Ce-MOFs.^57,58^

**Fig. 3:**
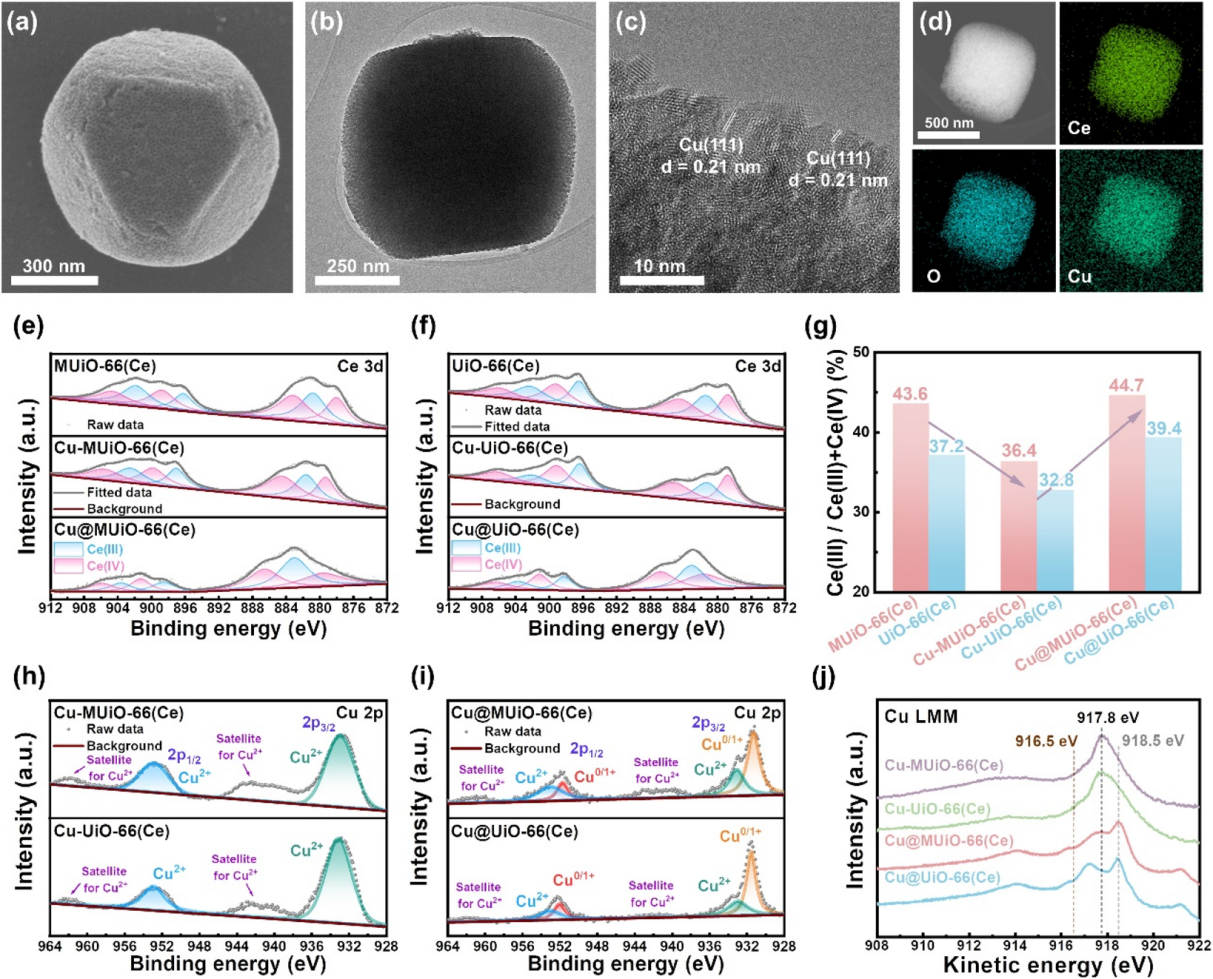
(a) High-magnification SEM image, (b) TEM image, and (c) HRTEM image of Cu@MUiO-66(Ce). (d) EDS elemental mapping data for Ce, O, and Cu of Cu@MUiO-66(Ce). XPS spectra of (e) MUiO-66(Ce) (top), Cu-MUiO-66(Ce) (middle), Cu@MUiO-66(Ce) (bottom), and (f) UiO-66(Ce) (top), Cu-UiO-66(Ce) (middle), Cu@UiO-66(Ce) (bottom) collected in the region of Ce 3d. (g) The ratios of Ce(iii) in all cerium atoms for all samples, extracted from the data shown in (e and f). XPS spectra of (h) Cu-MUiO-66(Ce) (top), Cu-UiO-66(Ce) (bottom), and (i) Cu@MUiO-66(Ce) (top), Cu@UiO-66(Ce) (bottom) collected in the region of Cu 2p. (j) Cu LMM Auger spectra of Cu-MUiO-66(Ce), Cu-UiO-66(Ce), Cu@MUiO-66(Ce), and Cu@UiO-66(Ce).

Carbon paper-based electrodes modified with Cu-MUiO-66(Ce) or Cu-UiO-66(Ce) were then prepared by drop-casting technique using Nafion/ethanol as the binder, and the *in situ* formation of metallic copper nanoparticles was further conducted by a chronoamperometric method under a potential of −1.51 V *vs.* Ag/AgCl/KCl (sat'd) for 2 h. After the electrochemical pretreatment, no significant morphological changes can be observed for Cu@MUiO-66(Ce) and Cu@UiO-66(Ce) (Fig. 3a and S12†), and the crystallinities of these two samples are preserved (Fig. S13†). The absence of metallic copper signals in the XRD patterns may be due to its content being below the detection limit of the instrument.^46^ The TEM image of Cu@MUiO-66(Ce) suggests that the mesoporous structure can still be retained after the electrochemical pretreatment (Fig. 3b), and the HRTEM image shown in Fig. 3c further confirms the formation of metallic copper nanoparticles with a lattice of the (111) plane (*d* = 0.21 nm). Thereafter, EDS elemental mapping data were collected with the use of TEM, revealing a uniform distribution of Cu within the entire Cu@MUiO-66(Ce) particle, as shown in Fig. 3d. On the other hand, the lattice of the (111) plane of metallic copper as well as the spatially separated copper element throughout the particle can also be observed for Cu@UiO-66(Ce) (Fig. S14 and S15†).

Thereafter, X-ray photoelectron spectroscopic (XPS) measurements were conducted to probe the chemical compositions of materials and confirm the coordination between the copper sites and hexa-cerium nodes. XPS spectra of both MUiO-66(Ce) and UiO-66(Ce) in the C 1s region are shown in Fig. S16a and b.† The peaks located at around 283.8, 284.9, 287.8, and 290.5 eV are associated with the C–C, C–O, C=O, and O–C=O bonds, respectively, which confirm the chemical composition of the organic linker in MUiO-66(Ce) and UiO-66(Ce).^59^ Fig. S16c and d† show the O 1s spectra of MUiO-66(Ce) and UiO-66(Ce), where three peaks at around 531.0, 531.8, and 532.7 are attributed to the Ce–O, O–C=O, and H_2_O/–OH bonds, respectively.^60^ It should be noticed that around 29.4% of O atoms in MUiO-66(Ce) are in the form of H_2_O/–OH according to the integrated areas of the fitted peaks, while only 19.6% are in this form for those in UiO-66(Ce). Findings here disclose that a higher amount of –OH/–OH_2_ pairs exist in MUiO-66(Ce) compared to UiO-66(Ce), which is consistent with the results obtained from the titration experiments and confirms the presence of more open coordination sites within MUiO-66(Ce). Ce 3d XPS spectra of all MOF-based samples are shown in Fig. 3e and f, which reveal that both Ce(iv) and Ce(iii) are present in all samples. The fractions of Ce(iii) in all cerium atoms for each material were subsequently estimated based on the integrated areas of the fitted peaks, as shown in Fig. 3g. It is revealed that the Ce(iii) fractions for MUiO-66(Ce) and UiO-66(Ce) are 43.6% and 37.2%, respectively. The presence of Ce(iii) is supposed to be attributed to the partial reduction of cerium atoms that are coordinated to the –OH/–OH_2_ pairs by the solvent during synthesis,^33,61,62^ and MUiO-66(Ce) with more open coordination sites results in a higher content of Ce(iii) compared to UiO-66(Ce). The structure of Ce-MOFs is expected to remain unchanged in the presence of both Ce(iii) and Ce(iv) ions in the hexa-cerium clusters, as reported in previous work.^63^ After the SIM process, the Ce(iii) fractions decrease to 36.4% for Cu-MUiO-66(Ce) and 32.8% for Cu-UiO-66(Ce), indicating the lower electron density on cerium atoms when coordinated with copper ions. However, increments in the Ce(iii) fractions are found after the electrochemical pretreatment, with up to 44.7% and 39.4% of cerium atoms in the valence state of Ce(iii) for Cu@MUiO-66(Ce) and Cu@UiO-66(Ce), respectively. Such observation suggests that the chronoamperometric pretreatment with an applied potential more negative than the potential reported for the faradaic reaction of Ce^3+^/Ce^4+^ may partially reduce cerium atoms.^64^ To investigate the valence states of installed copper, XPS spectra of Cu-MUiO-66(Ce) and Cu-UiO-66(Ce) in the Cu 2p region are shown in Fig. 3h. Two peaks with binding energies at around 933.0 and 953.1 eV reveal that copper atoms coordinated on hexa-cerium nodes of frameworks mainly exist in the valence state of Cu(ii).^65^ Besides, two humps appearing at 937.5–947.0 eV and 957.8–964.0 eV are consistent with satellite signals usually observed for CuO-based materials.^66^ After the electrochemical pretreatment, the valence states of copper in Cu@MUiO-66(Ce) and Cu@UiO-66(Ce) were also probed in the Cu 2p region, as shown in Fig. 3i. Peaks corresponding to Cu(0)/Cu(i) at 931.4 and 951.7 eV as well as Cu(ii) at 933.1 and 953.0 eV were deconvoluted from the spectrum of Cu@MUiO-66(Ce),^67^ where the integrated areas of the fitted peaks indicate that the majority of copper atoms here exists in the reduced states. Both XPS signals of Cu(0)/Cu(i) as well as Cu(ii) can also be found in the spectrum of Cu@UiO-66(Ce), which exhibits a similar result with copper atoms primarily present in reduced states as Cu@MUiO-66(Ce). Nevertheless, it is challenging to distinguish between metallic Cu(0) and reduced Cu(i) states in the Cu 2p region due to their similar binding energies.^58^ Therefore, XPS spectra of copper-containing samples for the Cu LMM Auger region were collected (Fig. 3j). Both spectra of Cu-MUiO-66(Ce) and Cu-UiO-66(Ce) exhibit major peaks located at a kinetic energy of 917.8 eV, which is similar to those reported for CuO and indicates the predominant Cu(ii) of the copper atoms. On the other hand, noticeable peaks located at 918.5 eV in the spectra of Cu@MUiO-66(Ce) and Cu@UiO-66(Ce) confirm the presence of metallic Cu(0) in these materials. Additionally, these spectra also exhibit extra peaks at binding energies higher than Cu(i) (916.5 eV) but lower than Cu(ii) (917.8 eV),^68^ indicating that a portion of copper atoms are present as both Cu(i) and Cu(ii). Findings here reveal that the copper atoms were primarily reduced to Cu(0), with only a minor amount of Cu(i) and Cu(ii) existing in the samples after the electrochemical pretreatment. It should be noted that the Cu(ii) ions in Cu@MUiO-66(Ce) and Cu@UiO-66(Ce) that are not accessible to electrons *via* the redox-hopping process during electrochemical pretreatment will be electrochemically inactive and will not participate in the nitrate reduction reaction.

### Electrochemical performance

**Fig. 4:**
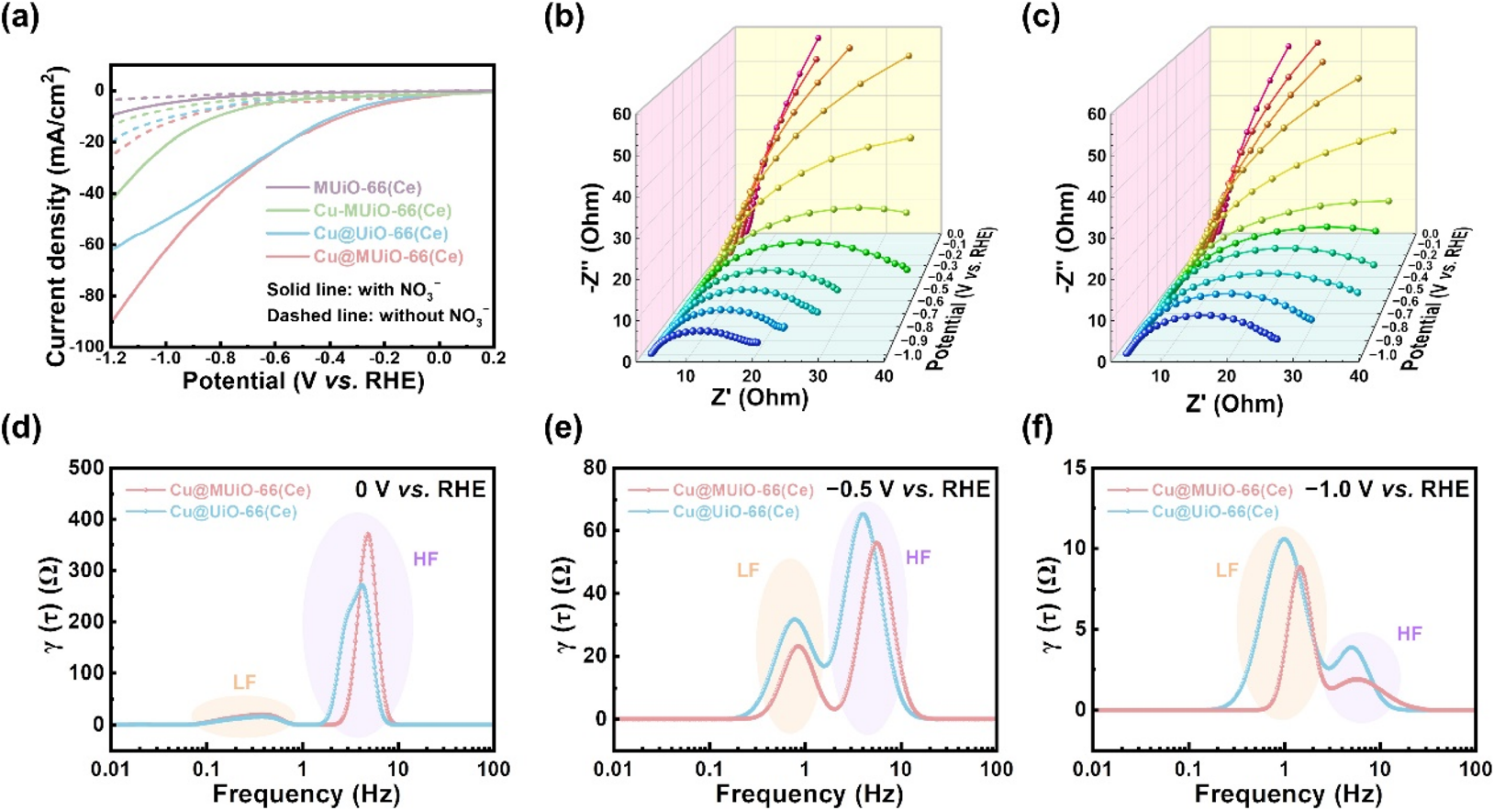
(a) LSV curves of the MUiO-66(Ce)-modified electrode, Cu-MUiO-66(Ce)-modified electrode, Cu@UiO-66(Ce)-modified electrode, and Cu@MUiO-66(Ce)-modified electrode, measured in 0.5 M Na_2_SO_4_ (aq) with/without 10 mM NaNO_3_ at a scan rate of 10 mV s^−1^. *Operando* EIS spectra of (b) Cu@MUiO-66(Ce)-modified electrode and (c) Cu@UiO-66(Ce)-modified electrode, measured in 0.5 M Na_2_SO_4_ (aq) with 10 mM NaNO_3_ at applied potentials ranging from 0 V to −1.0 V *vs.* RHE. DRT plots for the modified electrodes of Cu@MUiO-66(Ce) and Cu@UiO-66(Ce) at applied potentials of (d) 0 V, (e) −0.5 V, and (f) −1.0 V *vs.* RHE, extracted from the EIS plots shown in (b and c).

To evaluate the performance of electrocatalytic nitrate reduction to ammonia, carbon paper-based modified electrodes with a mass loading of 0.96 mg cm^−2^ were fabricated, and a typical three-electrode H-cell setup with 15 mL of aqueous solution containing 0.5 M of Na_2_SO_4_ and 10 mM of NaNO_3_ as the electrolyte in both compartments was applied; similar electrochemical setups using low concentrations of nitrate in Na_2_SO_4_ aqueous solution for electrochemical nitrate reduction to ammonia has also been reported for other MOF-supported metallic copper nanoparticles.^46,69^ (see experimental details in the ESI†). Linear sweep voltammetric (LSV) measurements were first performed in electrolytes with and without nitrate to assess the activity of each material during the electrolytic process (Fig. 4a). The LSV curves of each modified electrode show an enhancement in current density upon the addition of nitrate, which suggests the catalytic activities of these materials for nitrate reduction. Compared to the MUiO-66(Ce)-modified electrode, the Cu-MUiO-66(Ce)– and Cu@MUiO-66(Ce)-modified electrodes exhibit a noticeable enhancement in catalytic current, indicating that the copper-based species are highly active electrocatalysts for nitrate reduction. Moreover, Cu@MUiO-66(Ce), which possesses copper species in reduction states, exhibits electrocatalytic activity that remarkably outperforms Cu-MUiO-66(Ce) with copper species only in the state of Cu(ii); the related study of reduced copper species with superior nitrate-to-ammonia electrocatalytic activity compared to Cu(ii) has been reported in the previous literature.^70^ For comparison, LSV curves of the Cu@UiO-66(Ce)-modified electrode were also collected, which demonstrate similar current signals to those of the Cu@MUiO-66(Ce)-modified electrode in the potential range of +0.2 to −0.7 V *vs.* RHE. However, the enhancement of the catalytic current is more pronounced for the Cu@MUiO-66(Ce)-modified electrode in the potential range of −0.7 to −1.2 V *vs.* RHE. Findings here reveal that the diffusion of reactants within the Cu@UiO-66(Ce) thin film becomes the primarily limitation of the catalytic reaction when the potential is more negative than −0.7 V *vs.* RHE, and the Cu@MUiO-66(Ce)-modified electrode, with additional mesopores within the entire crystals and more copper active sites, can facilitate the mass transport of nitrate and enhance the catalytic current. Cyclic voltammetric (CV) curves for the Cu@MUiO-66(Ce)– and Cu@UiO-66(Ce)-modified electrodes were collected at various scan rates within the potential window of the non-faradaic current region (Fig. S17†), and the respective double-layer capacitances were then estimated according to the slope shown in Fig. S18.† The Cu@MUiO-66(Ce)-modified electrode demonstrates a double-layer capacitance of 159 μF cm^−2^, which is higher than the 44 μF cm^−2^ observed for the Cu@UiO-66(Ce)-modified electrode. Typically, the electrochemically active surface area (ECSA) of an electrode is proportional to the double-layer capacitance,^71^ and it should be mentioned that contrary to the BET surface area, the ECSA here reflects the surface area that is electrochemically available for reactants or ions in the electrolyte, which depends not only on the surface area and porosity of the material but also on its conductivity. Since Ce-MOF scaffolds are porous but completely electrically insulating for electrons, the ECSA attributed to Ce-MOF scaffolds is negligible and should solely come from the metallic copper and the underlying carbon paper substrate, where the Cu@MUiO-66(Ce)-modified electrode with a higher loading of copper nanoparticles could possess a higher amount of accessible metallic copper sites compared to the Cu@UiO-66(Ce)-modified electrode.

*Operando* electrochemical impedance spectra (EIS) at different applied potentials were obtained to probe the charge transfer kinetics and the mass transfer behaviors within the thin films of Cu@MUiO-66(Ce) and Cu@UiO-66(Ce) (Fig. 4b and c). Both modified electrodes demonstrate large impedance semicircles at high frequencies before −0.3 V *vs.* RHE, suggesting that the charge transfer reaction is not sufficiently promoted at low potentials. As the potential is negatively increased, the impedance semicircles significantly shrink and suggest the boosting charge transfer reaction of nitrate reduction process. Thereafter, the distribution of relaxation time (DRT) analysis was conducted using a Gaussian basis function based on the EIS data, which renders a model-free approach to probe the resistance of the system without relying on equivalent circuits.^72^ The DRT plots derived from the EIS data at applied potentials of 0 V, −0.5 V, and −1.0 V *vs.* RHE for both modified electrodes are shown in Fig. 4d–f. Two distinct peaks in the low frequency (LF) and high frequency (HF) regions can be distinguished in these plots, which are related to the mass transfer process and the charge transfer process involved in the catalytic reaction, respectively.^73^ When the potential is applied at 0 V *vs.* RHE, both modified electrodes demonstrate a major peak with a large underlying area in the HF region, suggesting that the charge transfer reaction is not sufficiently promoted at this potentials, so this reaction serves as the rate-limiting step (Fig. 4d). However, at −0.5 V *vs.* RHE, the peaks in the HF region decrease significantly but still retain a larger underlying area compared to those in the LF region, indicating that the charge transfer resistance becomes smaller, while it still serves as the rate-limiting step (Fig. 4e). Besides, the Cu@MUiO-66(Ce)-modified electrode exhibits smaller peaks than those of the Cu@UiO-66(Ce)-modified electrode in both the HF and LF regions, which implies that Cu@MUiO-66(Ce), with a high loading of active copper sites and mesoporous channels, can achieve superior catalytic performance with enhanced charge transfer and mass transfer in the reaction. For the potential applied at −1.0 V *vs.* RHE, the mass transfer process turns out to be the rate-determining step, and Cu@UiO-66(Ce) displays a major peak with a much larger underlying area and lower frequency than Cu@MUiO-66(Ce) in the LF region, revealing that the diffusion of nitrate can be strongly promoted in the ordered mesopores (Fig. 4f).^74^ Findings here suggest that the overall reaction converts from charge transfer-controlled step to mass transfer-controlled step as the overpotential increases, and Cu@MUiO-66(Ce) with facile diffusion of reactants from the electrolyte to the catalytic sites can outperform Cu@UiO-66(Ce) for nitrate reduction at high overpotentials.

**Fig. 5:**
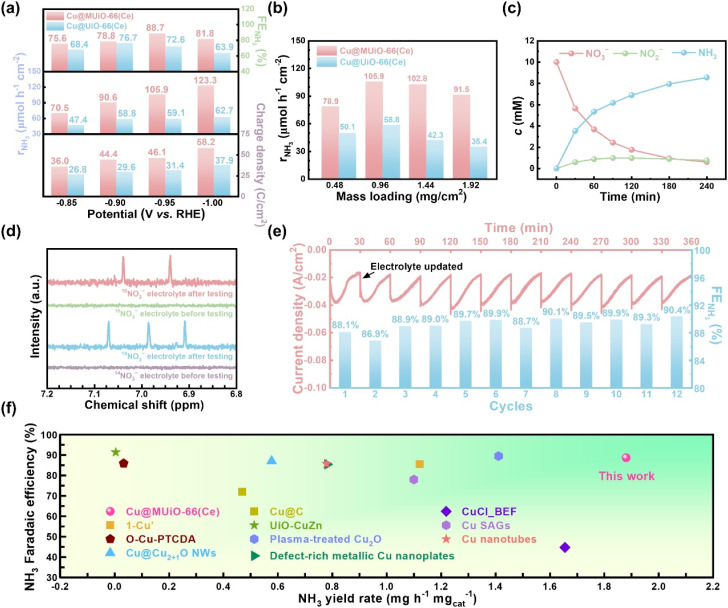
(a) The integrated charge density, *r*_NH_3__, and FE_NH_3__ for electrocatalysis using the modified electrodes of Cu@MUiO-66(Ce) and Cu@UiO-66(Ce) at different applied potentials for 30 min. (b) The *r*_NH_3__ values achieved by modified electrodes with different mass loadings of Cu@MUiO-66(Ce) and Cu@UiO-66(Ce) for 30 min of electrocatalysis. The applied potentials were −0.95 and −0.90 V *vs.* RHE for Cu@MUiO-66(Ce)– and Cu@UiO-66(Ce)-modified electrodes, respectively. (c) Concentration evolution curves of nitrate, nitrite, and ammonia in the electrolyte during the electrolysis at −0.95 V *vs.* RHE for 4 h, extracted from the data shown in Fig. S22.† (d) ^1^H NMR spectra of samples prepared with electrolytes after 30 min of electrolysis, with the use of Cu@MUiO-66(Ce) as the electrocatalyst as well as Na^14^NO_3_ and Na^15^NO_3_ as N sources in the electrolyte, respectively. Spectra of the samples before electrolysis are also shown. (e) Amperometric curves and corresponding FE_NH_3__ for 12 consecutive cycles of electrolysis at −0.95 V *vs.* RHE with 10 mM nitrate using the Cu@MUiO-66(Ce)-modified electrode, with electrolyte refreshment every 30 min. (f) Comparison of ammonia yield rate and faradaic efficiency for Cu@MUiO-66(Ce) with 1-Cu,^46^ O–Cu-PTCDA,^76^ Cu@Cu_2+1_O NWs,^77^ Cu@C,^78^ UiO-CuZn,^69^ plasma-treated Cu_2_O,^79^ defect-rich metallic Cu nanoplates,^80^ CuCl_BEF,^81^ Cu SAGs,^82^ and Cu nanotubes^41^ reported in literature.

Subsequently, electrolytic experiments for the modified electrodes of Cu@MUiO-66(Ce) and Cu@UiO-66(Ce) were performed at various applied potentials for 30 min with a constant stirring speed of 500 rpm, and the concentrations of nitrate, nitrite, and ammonia in the electrolyte after each electrolytic experiment were determined by UV-visible spectroscopic methods with well-established calibration curves (see experimental details and Fig. S19 and S20 in the ESI†).^69,75^ The integrated charge density, yield rate of ammonia (*r*_NH_3__), and faradaic efficiency for NH_3_ production (FE_NH_3__) were then calculated (see equations in the ESI†), and the results are shown in Fig. 5a. The overall charge density passing through the Cu@MUiO-66(Ce)-modified electrode during the electrolysis for 30 min is generally larger than that of the Cu@UiO-66(Ce)-modified electrode at the same applied potential, suggesting that the Cu@MUiO-66(Ce) electrocatalyst possesses higher catalytic activities for either nitrate reduction or hydrogen production compared to the Cu@UiO-66(Ce) electrocatalyst. The *r*_NH_3__ for the Cu@MUiO-66(Ce)-modified electrode constantly enhances with the increasing overpotential, and an *r*_NH_3__ of 123.3 μmol h^−1^ cm^−2^ can be achieved at an applied potential of −1.00 V *vs.* RHE. However, the enhancement of *r*_NH_3__ for the Cu@UiO-66(Ce)-modified electrode is significantly limited when the applied potential is more negative than −0.90 V *vs.* RHE, and only an *r*_NH_3__ of 62.7 μmol h^−1^ cm^−2^ is achieved for the Cu@UiO-66(Ce)-modified electrode at −1.00 V *vs.* RHE. At −0.85 and −0.90 V *vs.* RHE, the ratios of *r*_NH_3__ between the Cu@MUiO-66(Ce)– and Cu@UiO-66(Ce)-modified electrodes are around 1.5, while it increases to around 1.8 and nearly 2.0 at −0.95 and −1.00 V *vs.* RHE, respectively. It should be noted that the Cu loading in Cu@MUiO-66(Ce) is 1.69 times higher compared to that in Cu@UiO-66(Ce). Findings here suggest that the enhanced production rate of ammonia at −0.95 and −1.00 V *vs.* RHE, where a significant limitation from the diffusion of nitrate is present, not only comes from the higher Cu loading, but also results from the mesopore facilitating the mass transfer. The Cu@MUiO-66(Ce)-modified electrode exhibits a volcanic tendency in FE_NH_3__, reaching a maximum value of 88.7% at −0.95 V *vs.* RHE, with an *r*_NH_3__ of 105.9 μmol h^−1^ cm^−2^ and a specific activity of 1.875 mg_NH_3__ h^−1^ mg_catalyst_^−1^. In contrast, a lower FE_NH_3__ of 76.7% is achieved by the Cu@UiO-66(Ce)-modified electrode with an optimized potential of −0.90 V *vs.* RHE, suggesting the competitive HER is more pronounced for the Cu@UiO-66(Ce) electrocatalyst compared to the Cu@MUiO-66(Ce) electrocatalyst when a constant charge is applied. The turnover frequencies (TOF) for NH_3_ production were calculated, and the Cu@MUiO-66(Ce) electrocatalyst exhibits a value of 98.48 h^−1^ at −0.95 V *vs.* RHE, which is comparable to the 90.27 h^−1^ observed for the Cu@UiO-66(Ce) electrocatalyst at −0.90 V *vs.* RHE. Such TOF results suggest that the electrocatalytic activities of the copper species in both MUiO-66(Ce) and UiO-66(Ce) are similar, which is attributed to the similar valence states of the copper species in both electrocatalysts. Nevertheless, it should be noted that the copper loading in Cu@MUiO-66(Ce) is 1.69 times higher than that in Cu@UiO-66(Ce), and such higher copper content can contribute to a greater quantity of ammonia production, even with similar TOF values. Additionally, the *r*_NH_3__ values achieved by MUiO-66(Ce)– and UiO-66(Ce)-modified electrodes are shown in Fig. S21.† Findings here suggest that only minor catalytic performance is attributed to the cerium sites in Cu@MUiO-66(Ce) and Cu@UiO-66(Ce). Modified electrodes with different mass loadings of Cu@MUiO-66(Ce) and Cu@UiO-66(Ce) were then performed, and the resulting *r*_NH_3__ values for these electrodes after 30 min of electrolysis are shown in Fig. 5b. It can be observed that *r*_NH_3__ increases with the increasing mass loading of Cu@MUiO-66(Ce) electrocatalyst on the carbon-paper substrate from 0.48 to 0.96 mg cm^−2^, which is attributed to the more copper active sites present on the modified electrode. However, *r*_NH_3__ begins to decrease slightly when the mass loading of Cu@MUiO-66(Ce) exceeds 0.96 mg cm^−2^, suggesting that the increased mass loading may hinder the diffusion of nitrate from the external electrolyte and start to limit the overall reaction. On the other hand, the mass loading of Cu@UiO-66(Ce) electrocatalyst also exhibits a volcano-shaped trend in *r*_NH_3__ but with lower values at all mass loadings compared to the Cu@MUiO-66(Ce) electrocatalyst. Besides, the difference in *r*_NH_3__ between the two electrocatalysts becomes more pronounced with increasing mass loading. A dramatic decrease of 39.8% in *r*_NH_3__, from 58.8 to 35.4 μmol h^−1^ cm^−2^, is observed for the Cu@UiO-66(Ce)-modified electrode as the loading increases from 0.96 to 1.92 mg cm^−2^. In contrast, the decrease for Cu@MUiO-66(Ce) is only 13.6%, suggesting that the diffusion of nitrate within the modified thin film is strongly hindered by the microporous nature of Cu@UiO-66(Ce). Findings here reveal that Cu@MUiO-66(Ce) with additional mesoporous channels facilitating the mass transfer of reactants can achieve better utilization of the loaded active sites during the electrolysis. The electrocatalytic activity of the Cu@MUiO-66(Ce) electrocatalyst was assessed at −0.95 V *vs.* RHE for 4 h, and the concentration evolution of nitrate, nitrite, and ammonia in the electrolyte were monitored by UV-visible spectroscopic methods (Fig. S22†). As shown in Fig. 5c, the concentration of nitrate keeps decreasing with the increasing concentration of ammonia, which suggests the efficient conversion of nitrate to ammonia. Meanwhile, the concentration of nitrite increases as the concentration of nitrate decreases at first 90 min, suggesting that nitrite is an intermediate for ammonia production. After 90 min of electrolysis, the concentration of nitrate decreases from 10 mM to 2.4 mM. With the insufficiency of the nitrate source, the production rate of nitrite becomes slower than the consumption rate, which leads to a decline in nitrite concentration and indicates that the selectivity for the formation of ammonia is enhanced along with the prolonged electrolysis time. The conversion ratio of nitrate reduction and the selectivity for ammonia production were calculated, as shown in Fig. S23.† An inverted-volcano shape with the lowest selectivity for ammonia production, which corresponds to the highest concentration of nitrite in the electrolyte, can be found in the plot. Moreover, after the electrolysis for 4 h, a selectivity of 91.1% with a conversion ratio of 93.9% can be achieved by the Cu@MUiO-66(Ce)-modified electrode. Additionally, the XRD pattern of the Cu@MUiO-66(Ce) from the resulting electrode indicates that the crystallinity of the MOF can be preserved after 4 h of electrolysis (Fig. S24†).

A blank experiment was conducted using the Cu@MUiO-66(Ce)-modified electrode for 30 min of electrolysis at −0.95 V *vs.* RHE in Ar-saturated electrolytes with or without 10 mM of nitrate (Fig. S25†). An ammonia concentration of 3.53 mM is detected in the nitrate-containing electrolyte, while negligible ammonia production is observed in the nitrate-free electrolyte. Moreover, ^15^N isotope labeling experiments were carried out to trace the source of ammonia. The Cu@MUiO-66(Ce)-modified electrode was subjected to electrolysis at −0.95 V *vs.* RHE for 30 min with the use of Na^14^NO_3_ and Na^15^NO_3_ as N sources in the electrolyte, respectively, and the resulting electrolytes were then subjected to ^1^H nuclear magnetic resonance (^1^H NMR) measurements (Fig. 5d). The spectra display triple peaks of ^14^NH_4_^+^ when Na^14^NO_3_ is served as the reactant in the electrolyte, while only double peaks of ^15^NH_4_^+^ for the adoption of Na^15^NO_3_. Both the blank experiment and isotope labeling results verify the produced ammonia originated from the reduction of nitrate in the electrolyte.

The durability of the Cu@MUiO-66(Ce) electrocatalyst for nitrate reduction to ammonia was evaluated through twelve consecutive cycles of electrolysis with the electrolyte refreshed every 30 min (Fig. 5e). The current density gradually declines for each cycle, indicating the consumption of nitrate in the electrolyte. After refreshing the electrolyte, the current density returns to a relatively high level and reveals the sustained activity of the electrocatalyst. Furthermore, no significant change in FE_NH_3__ is observed over twelve cycles of electrolysis, which demonstrates the excellent electrochemical durability of Cu@MUiO-66(Ce) for ammonia production. Electrocatalytic performances achieved by the Cu@MUiO-66(Ce) electrocatalyst are compared with those reported for Cu-based electrocatalysts, as summarized in Fig. 5f and Table S5†.^36,41,46,65,69,76–85^ Benefiting from the high loading of copper active sites and the facile diffusion of reactants, superior electrocatalytic nitrate-to-ammonia activities can be achieved by the Cu@MUiO-66(Ce)-modified electrode.

### Molecular dynamics simulations

**Fig. 6:**
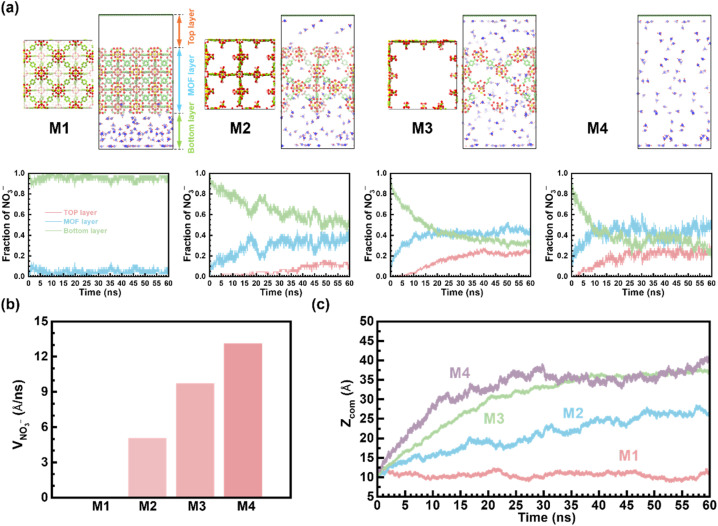
(a) Top and side views of the M1–M4 models. The side views are taken from snapshots at 60 ns. Yellow, red, green, and blue balls represent Ce, O, C, and N atoms, respectively. Hydrogen atoms of MOF and water molecules are omitted for clarity. The simulation cells of M1, M2, and M4 were duplicated to improve visibility. The subplots show the fraction of NO_3_^−^ ions in each region (bottom, MOF, and top) throughout the simulations. (b) The *z* component of NO_3_^−^ velocity (in Å ns^−1^) within the MOF region in M1–M4. (c) *z*-component of the center-of-mass (in Å) of NO_3_^−^ during the simulations in M1–M4 models.

Since mass transfer could play an important role in electrochemical performance, we studied the diffusion behavior of NO_3_^−^ using molecular dynamics (MD) simulations. To simplify the simulations, four UiO-66(Ce) models (M1–M4) with pore sizes ranging from nanoscale to macroscale were used, as depicted in Fig. 6a. Model M1 represents a defect-free UiO-66(Ce), while models M2 and M3 represent a defective UiO-66(Ce) with micropores and an extended pore, respectively. The last model, M4, represents the framework with a macropore. The details of these models and the MD simulation parameters are provided in the ESI.† To quantify the movement of NO_3_^−^ through the pores, we tracked the number of NO_3_^−^ ions in each region (bottom, middle MOF, and top) throughout the simulations. Additionally, we calculated the *z* component of NO_3_^−^ velocity in MOFs (Fig. 6b). This velocity was determined by measuring the time NO_3_^−^ travels in the MOF region (from *z* = 20 Å to *z* = 60 Å) and dividing the thickness of the MOF slab (40 Å) by this residence time. Furthermore, the center-of-mass of all NO_3_^−^ ions during the simulations was tracked (Fig. 6c) to provide a more comprehensive analysis of their movement within the MOFs.

The MD simulations clearly confirm a strong influence of the pore size on the transport property. Micropores in defect-free M1 severely restrict the diffusion, as evidenced by the absence of NO_3_^−^ ions in the top region even after 60 ns. This slow diffusion is likely due to the combined effect of the limited pore size and the modest rotational barrier of the BDC linker (∼12 kcal mol^−1^),^86,87^ which governs the gate-opening mechanism in MOF.^88^ Consequently, NO_3_^−^ velocity in M1 is negligible. As the pore size increases to around 16 Å (M2), NO_3_^−^ diffusion clearly accelerates, reaching a velocity of 5.1 Å ns^−1^. However, the velocity in M2 is still well below the limiting velocity in M4 (13.1 Å ns^−1^). The finding suggests that further improvement in diffusion rates might be achievable by introducing mesopores. Indeed, the results of M3 highlight this strategy. Compared to M2, the window size of M3 increases by around 2.3 times (38 Å), whereas the velocity continues to increase by 1.9 times to 9.7 Å ns^−1^. This value is now approaching the limiting velocity of 13.1 Å ns^−1^, which should also be the velocity of NO_3_^−^ in the mesoporous MUiO-66(Ce) with pores size of 11.7 nm. The diffusion behavior of NH_3_ using MD simulations is also demonstrated in Fig. S26,† showing a trend similar to that observed with NO_3_^−^. To conclude, the MD simulations conclusively demonstrate a strong correlation between the pore size and mass transport. These findings suggest that tailoring UiO-66(Ce) porosity offers a powerful strategy to improve mass transfer and enhance the electrochemical performance.

## Conclusions

Open coordination sites on the hexa-cerium nodes of a Ce-based MOF can be unlocked by a pore engineering strategy, where abundant terminal –OH/–OH_2_ pairs are present after the removal of soft-template agents. A high loading of copper sites can be further installed on the framework through post-synthetic modification. Computational studies indicate that a proton transfers from the –OH_2_ of the first terminal pair to the –OH of the second pair during the installation of copper ions, rendering a more favorable binding scenario when there are more open coordination sites on the nodes. Copper nanoparticles can be confined within the framework through the electrochemical pretreatment, and the modified electrode of the copper-based electrocatalyst is then subjected to electrochemical nitrate-to-ammonia reduction. Featuring the facile mass transport of reactants provided by the mesoporous channels, enhanced electrocatalytic activities can be achieved at relatively high overpotentials. With an optimized thin-film thickness, a maximal FE_NH_3__ of 88.7%, an *r*_NH_3__ of 105.9 μmol h^−1^ cm^−2^, and a specific activity of 1.875 mg_NH_3__ h^−1^ mg_catalyst_^−1^ can be achieved at an applied potential of −0.95 V *vs.* RHE for 30 min under a low nitrate concentration condition. Additionally, it exhibits a high conversion ratio of 93.9% and a selectivity of 91.1% after 4 h of electrolysis. Findings here shed light on the use of mesoporous MOFs for unlocking the open coordination sites of nodes and facilitating the mass transfer of reactants, where the rational design of mesoporous properties can be a promising strategy to enhance the performance of MOF-based materials for electrocatalysis.

## Data availability

The data supporting this article are included in the ESI† and are also available from the corresponding author upon reasonable request.

## Author contributions

C.-H. S. and Y. Z. conceived the project. C.-H. S. performed the experiments, analyzed the data and drafted the manuscript. Y. Z., L. Z., M. K., D. J., X. W. and T. Y. helped collect some of the experimental data. H. N. N., Q. M. P. and V. A. helped the simulations. C.-W. K. and Y. Y. supervised the research. All authors have given approval to the final version of the manuscript.

## Conflicts of interest

There are no conflicts to declare.

## Supplementary Material

SC-OLF-D4SC07132H-s001
