# Supplementary material for: Unlocking coordination sites of metal–organic frameworks for high-density and accessible copper nanoparticles toward electrochemical nitrate reduction to ammonia
Source: Chem Sci. 2025 Mar 6;16(16):7026–38. doi: 10.1039/d4sc07132h (PMC11934058; doi:10.1039/d4sc07132h)
Supplement: SC-016-D4SC07132H-s001 [file SC-016-D4SC07132H-s001.pdf]

Supplementary Information (SI) for Chemical Science.
This journal is © The Royal Society of Chemistry 2025

Supporting Information

Unlocking Coordination Sites of Metal−Organic Frameworks for High-Density and Accessible Copper Nanoparticles toward Electrochemical Nitrate Reduction to Ammonia

Cheng-Hui Shen, Yingji Zhao, Ho Ngoc Nam, Liyang Zhu, Quan Manh Phung, Vic Austen, Minjun Kim, Dong Jiang, Xiaoqian Wei, Tokihiko Yokoshima, Chung-Wei Kung,* and Yusuke Yamauchi**

C.-H. Shen, C.-W. Kung
Department of Chemical Engineering
National Cheng Kung University
1 University Road, Tainan City, 70101, Taiwan
E-mail: cwkung@mail.ncku.edu.tw

C.-H. Shen, Y. Zhao, H. N. Nam, L. Zhu, D. Jiang, X. Wei, T. Yokoshima, Y. Yamauchi
Department of Materials Process Engineering, Graduate School of Engineering
Nagoya University
Nagoya 464−8603, Japan
E-mail: zhao.yingji.n2@f.mail.nagoya-u.ac.jp, y.yamauchi@uq.edu.au

Q. M. Phung, V. Austen
Department of Chemistry, Graduate School of Science
Nagoya University
Nagoya 464−8603, Japan

Q. M. Phung

Institute of Transformative Bio-Molecules (WPI-ITbM)

Nagoya University

Nagoya 464−8603, Japan

M. Kim, Y. Yamauchi

Australian Institute for Bioengineering and Nanotechnology (AIBN) and School of Chemical Engineering

The University of Queensland

Brisbane, QLD 4072, Australia

1. Experimental section

1.1 Materials.

Ammonium cerium(IV) nitrate ($(NH_4)_2Ce(NO_3)_6$, 95%), copper(II) acetate monohydrate ($Cu(OAc)_2 \cdot H_2O$, 97%), sodium sulfate (Na_2SO_4, 99%), sodium nitrate ($NaNO_3$, 99%), sodium nitrite ($NaNO_2$, 97%), sodium hydroxide (NaOH, 97%), sulfamic acid (99%), sulfanilic acid (99%), trisodium citrate dihydrate (99%), salicylic acid (99.5%), sodium nitrate-^{15}N ($Na^{15}NO_3$, 98 % ^{15}N), sodium hypochlorite (5%), acetic acid (99.7%), nitric acid (HNO_3, 60%), hydrochloric acid (HCl, 35.0-37.0%), sulfuric acid (95%), hydrogen peroxide solution (H_2O_2, 30%), N,N-dimethylformamide (DMF, 99.5%), ethanol (99.5%), acetone (99%) were purchased from Wako Pure Chemical Corporation. Pluronic poly(ethylene oxide)$_{106}$−poly(propylene oxide)$_{70}$−poly(ethylene oxide)$_{106}$ ($PEO_{106}PPO_{70}PEO_{106}$, F127), terephthalic acid (H_2BDC, 98%), Nafion perfluorinated resin solution (5 wt% in lower aliphatic alcohols and water), sodium nitroferricyanide (≥99%), dimethyl sulfoxide-d$_6$ (DMSO-d$_6$, 99.9 atom% D) were purchased from Sigma-Aldrich. Sodium perchlorate monohydrate ($NaClO_4 \cdot H_2O$, 98%) was purchased from Strem Chemicals, Inc. N-(1-naphthyl)-ethylenediamine dihydrochloride (97%) was purchased from Combi-Blocks Inc.

Carbon paper substrates (thickness of 0.19 mm, Teflon treated: 10%) was purchased from Moubic Co. Deionized water was used as the water source throughout this work.

1.2 Synthesis of MUiO-66(Ce) and UiO-66(Ce).

The synthesis of MUiO-66(Ce) powder was carried out using a slightly modified version of reported procedures.[1] Briefly, 191 mg of F127 was added into a 50 mL scintillation vial with 46 mL of deionized water. After sonicated for 10 min, 0.574 mL of acetic acid, 3825 mg of $NaClO_4 \cdot H_2O$, 1.1 mL of HNO_3, and 1048 mg of $(NH_4)_2Ce(NO_3)_6$ were successively added into the resulting solution. The mixture was then sonicated for 10 min to obtain a homogeneous solution. Thereafter, 317 mg of H_2BDC was added into the obtained solution, and the resulting mixture was heated in an oil bath at 60 °C under stirring at 500 rpm for 20 min. After the MOF growth, the obtained solid was washed with 40 mL of DMF for three times. Subsequently, the as-synthesized sample was soaked in 40 mL of ethanol for one day to remove the F127 template. Then, the obtained solid was subjected to solvent exchange with 40 mL of acetone three times, with the immersion period of 2 h, overnight, and 2 h, respectively. Finally, the MUiO-66(Ce) powder was activated in a vacuum oven at 60 °C overnight.

The synthesis of UiO-66(Ce) was according to previously published procedures with a slight modification.[2] First, 1170 mg of $(NH_4)_2Ce(NO_3)_6$ was dissolved in 4 mL of deionized water by sonication. The obtained solution was added into a 20 mL scintillation vial containing 354 mg of H_2BDC dispersed in 12 mL of DMF. The resulting mixture was heated at 100 °C in an oil bath for 15 min with stirring. After cooling down the suspension to room temperature, the solid was separated from the mother liquor by centrifugation. Subsequently, the obtained sample was washed with DMF for three times to remove residual reactants. Solvent exchange with acetone and thermal activation as described above were further applied, and the UiO-66(Ce) powder was then obtained.

1.3 Installation of copper sites in Ce-MOFs.

The spatially isolated copper sites were installed in Ce-MOFs using a solvothermal deposition in MOFs (SIM) method with some modifications from the published literature.[3] Firstly, 100 mg of copper(II) acetate monohydrate was dissolved in 30 mL of DMF by sonication, and 130 mg of Ce-MOFs was added into the resulting solution. The mixture was then heated at 80 °C with 300 rpm stirring for 24 h. After the reaction, the solid was washed with 30 mL of DMF for three times to remove the excess copper precursor. The obtained sample was subjected to solvent exchange and thermal activation, which are the same as those steps mentioned for the synthesis of Ce-MOFs. The resulting copper-decorated Ce-MOFs were designated as “Cu-MUiO-66(Ce)” and “Cu-UiO-66(Ce)”, respectively.

1.4 Preparation of modified electrodes.

The deposition of Cu-MUiO-66(Ce) and Cu-UiO-66(Ce) thin films on carbon paper substrates was performed using the drop-casting technique. First, 6 mg of Cu-MUiO-66(Ce) or Cu-UiO-66(Ce) was dispersed in the mixture containing 980 μL of ethanol and 20 μL of Nafion™ 117 by sonication. On the other hand, carbon paper substrates with a size of 1 cm × 2 cm were immersed in ethanol and sonicated for 10 min before thin-film deposition. Thereafter, 40 μL of the as-prepared suspension was drop-casted onto carbon paper substrates with a controlled area of 1 cm × 1 cm. The drop-casting process was performed on both sides of the carbon paper substrate for two times, and the modified electrodes of Cu-MUiO-66(Ce) and Cu-UiO-66(Ce) were thus obtained with a mass loading of 0.96 mg/cm^2.

The in situ clustering of copper nanoparticles in MUiO-66(Ce) and UiO-66(Ce) was further carried out by electrochemical reduction of the as-prepared modified electrodes. A three-electrode electrochemical setup at room temperature with an Ag/AgCl/KCl (sat’d) electrode as the reference electrode, a platinum wire as the counter electrode, and 30 mL of 0.5 M Na_2SO_4 aqueous solution as the electrolyte was applied. A chronoamperometric method under a potential of −1.51 V *vs.* Ag/AgCl/KCl (sat’d) for 2 h was used; the similar technique has been utilized for the reduction of copper atom within a Ce-MOF in the reported

literature.[4] The obtained modified electrodes were designated as "Cu@MUiO-66(Ce)" and "Cu@UiO-66(Ce)", respectively.

1.5 Instrumentations.

Powder X-ray diffraction (PXRD) patterns of samples were collected on a D2 PHASER 2nd Generation system (Bruker) operating at 30 kV voltage and 15 mA current with Cu K_α radiation (λ = 0.15406 nm). Scanning electron microscopy (SEM) images were collected by a GeminiSEM 560 (ZEISS) worked at 2 kV. Transmission electron microscopy (TEM) images, high-resolution TEM (HRTEM) images, and energy dispersive X-ray spectroscopy (EDS) elemental mapping data for Cu@MUiO-66(Ce) and Cu@UiO-66(Ce) were collected by a HF5000 (Hitachi). TEM and HRTEM images for MUiO-66(Ce), UiO-66(Ce), Cu-MUiO-66(Ce), and Cu-UiO-66(Ce) were collected by a JEM-ARM200F (JEOL). To prepare the samples of Cu@MUiO-66(Ce) and Cu@UiO-66(Ce) for XRD, SEM, TEM, and EDS measurements, the modified electrodes were sonicated in water for 10 s after the electrochemical pretreatment. The suspensions were then centrifugated to collect the solids. Thereafter, the obtained solids were immersed in the mixture of DMF and ethanol for 2 h. The powder samples were thus prepared after solvent-exchanged by acetone and activated in a vacuum oven. A selected-area electron diffraction (SAED) pattern was collected by JEM-1400 Flash (JEOL) under an accelerating voltage of 40 kV. Nitrogen adsorption−desorption isotherms were recorded at 77 K by Belsorp mini II (MicrotracBEL Corp.). The samples were degassed in a vacuum oven at 100 °C for 8 h before measurement. The specific surface area was calculated by the Brunauer-Emmett-Teller (BET) method. Pore size distributions were estimated using both the density functional theory (DFT) method and the Barrett−Joyner−Halenda (BJH) method. The small-angle X-ray scattering (SAXS) pattern was collected on a MicroMax-007HF (Rigaku) operating at 40 kV voltage and 30 mA current with Cu K_α radiation (λ = 0.15406 nm). X-ray photoelectron spectroscopic (XPS) data were collected using a PHI Quantes (Ulvac-PHI) equipped with an Al K_α X-ray radiation as the source gun. The binding energy data were calibrated by referencing the C 1s peak to 284.8

eV. Inductively coupled plasma-atomic emission spectrometry (ICP-AES) measurements were conducted by a SPS 7800 Plasma Spectrometer (SII−Seiko Instruments Inc.). The MOF-based samples were first digested by the piranha solution; the experimental details of sample preparation for measurements can be found in our previous work.[5] All electrical conductivity measurements were performed with a CHI6273E potentiostat (CH Instruments Inc.) at room temperature. Electrochemical impedance spectra (EIS) were recorded with a SP-300 Potentiostat (BioLogic), and all other electrochemical measurements were performed using a CHI 705EN electrochemical workstation (CHI Instruments, BAS Inc., Japan). Ultraviolet−visible (UV−vis) absorbance spectra were collected using a V-770 spectrometer (Jasco). ^{1}H nuclear magnetic resonance (NMR) spectra were measured on an AVANCE 500NMR spectrometer (Bruker). The pH values of titration experiments were determined by a LAQUA F-72 (HORIBA).

1.6 Titration experiments.

Prior to each titration, the pH meter was first calibrated using the standard solutions at pH values of 1.68, 4.01, 7.01, and 10.01, respectively. The details for the sample preparation can be found in our previous studies.[6] Briefly, 50 mg of the precisely weighted sample was activated in a vacuum oven at 80 °C for 8 h and transferred into a plastic beaker containing 50 mL of 0.01 M $NaNO_3$ aqueous solution. The mixture was stirred at 500 rpm for 18 h at room temperature. The pH value of the mixture was then adjusted to 3.0 with 0.1 M HCl. For the titration, a manual method by injecting a little amount (0.1 or 0.05 mL) of 0.1 M NaOH solution into the above mixture each time and measuring the pH value after stirring at 300 rpm for 5 min was conducted. The titration experiment was terminated until a total use of 2.4 mL of NaOH solution. The first derivative of the titration curves was calculated for the determination of the equivalence points.

1.7 Conductivity measurements.

For measuring the bulk electrical conductivity of each material, pellets of MUiO-66(Ce), UiO-66(Ce), Cu-MUiO-66(Ce) and Cu-UiO-66(Ce) were prepared using a mini-pellet press

with a 7-mm die set and a load of 0.5 ton; the experimental details for preparing and drying the MOF-based pellets can be found in our previous studies.[5, 7] The thickness of each pellet was measured by a digimatic caliper after collecting the current–voltage (I–V) curve of the pellet.

1.8 Electrochemical measurements.

All electrochemical measurements were conducted by using a three-electrode setup as mentioned previously with the carbon paper-based modified electrodes as the working electrode and 30 mL of 0.5 M Na_2SO_4 aqueous solution with different concentrations of $NaNO_3$ as the electrolyte. The applied potentials were adjusted to reversible hydrogen electrode (RHE), according to the Nernst equation (1):

$$E_{\mathrm{RHE}} = E_{\mathrm{experiment}} + 0.0592\mathrm{pH} + E_{\mathrm{Ag/AgCl}} \quad (1)$$

where a pH value of 7 was used.

For all electrolytic experiments, an H-type electrolytic cell with anode and cathode compartments separated by a Nafion NR212 membrane was used, and 15 mL of 0.5 M Na_2SO_4 aqueous solution with different concentrations of $NaNO_3$ were served as the electrolyte in both compartments. The electrolyte was purged with Ar gas for 15 min before electrocatalysis. Linear sweep voltammetric (LSV) curves were collected at a scanning rate of 10 mV/s from +0.2 V to −1.2 V *vs.* RHE without iR compensation. Cyclic voltammetric (CV) experiments were conducted under a potential window between +0.5 V and +0.6 V *vs.* RHE at various scan rates in 0.5 M Na_2SO_4 (aq). Electrolytic experiments were carried out for 30 min with a constant stirring speed of 500 rpm. For the Cu@MUiO-66(Ce)-modified electrode, the catalytic performance was also assessed for 4 h. The electrolyte in the cathode compartment was collected for the following product analysis. For EIS measurements, the data were collected with the frequency range from 100 kHz to 0.1 Hz; an applied amplitude of 10 mV was used. EIS measurements were performed from 0 V to −1.0 V *vs.* RHE with

−100 mV increment. The distribution of relaxation times (DRT) was further evaluated from EIS results.[8]

1.9 Product analysis.

The UV−vis spectrophotometer was utilized to quantify the concentration of nitrate, nitrite, and ammonia in the electrolytes according to the reported method.[9] The calibration curves of each ion were first established through the absorbance of 0.5 M Na_2SO_4 aqueous solution with different concentrations of $NaNO_3$, $NaNO_2$ or NH_4Cl. The procedures for determining the concentration of products are showing below:

For the detection of nitrate, 0.1 mL of the obtained electrolyte was added into a glass vial containing 4.9 mL of 0.5 M Na_2SO_4 to reach the detection range. Subsequently, 0.1 mL of 1 M HCl and 0.01 mL of 0.8 wt.% sulfamic acid solution were further added to the solution. The mixture was kept in darkness for 15 min, and the UV−vis spectrum was then collected. The absorbance of nitrate for determining the concentration was calculated according to the formula (2):

$$A = A_{220\ nm} - 2A_{275\ nm} \quad (2)$$

For the detection of nitrite, 500 mg of sulfanilic acid was mixed with 95 mL of deionized water and 5 mL of acetic acid. After sonication for 5 min, 5 mg of N-(1-naphthyl)-ethylenediamine dihydrochloride was dissolved in the obtained solution; the color reagent was thus prepared. To prepare the samples for UV−vis measurements, 200 μL of the obtained electrolyte was added into a glass vial containing 800 μL of 0.5 M Na_2SO_4 to reach the detection range. Thereafter, 4 mL of the prepared color reagent was added into the above solution. The UV−vis spectrum was collected after keeping the mixture in darkness for 15 min. The concentration was then determined through the absorbance obtained at a wavelength of 540 nm.

For the detection of ammonia, an indophenol blue method was applied.[10] Briefly, a solution containing 2 g of NaOH, 2.889 g of sodium citrate, and 2.889 g of salicylic acid

dissolved in 50 mL of deionized water was first prepared. Subsequently, 40 μL of the obtained electrolyte was diluted by 1.96 mL of 0.5 M Na_2SO_4 and further added with 2 mL of the as-prepared solution. Then, the obtained solution was mixed with 1 mL of 0.05 M sodium hypochlorite solution and 200 μL of 1.0 wt.% sodium nitroferricyanide. The UV−vis spectrum of the resulting solution was collected after leaving in darkness for 2 h, and the concentration was determined through the absorbance obtained at a wavelength of 655 nm.

1.10 Isotope labeling experiments.

For experiments using the ^{15}N isotope, $Na^{15}NO_3$ was served as the feeding N-source to confirm the source of ammonia. The same two-compartment H-cell setup with Cu@MUiO-66(Ce)-modified electrode as the working electrode was applied while 15 mL of aqueous solution containing 10 mM of $Na^{15}NO_3$ and 0.5 M of Na_2SO_4 was served as the electrolyte in each compartment. After the electrolysis at −0.95 V *vs.* RHE for 2 h, the electrolyte in the cathode compartment was collected and added with H_2SO_4 to adjust the pH value to weak acid. Subsequently, the obtained solution was mixed with DMSO-d_6, and the ^1H NMR spectrum of the resulting sample was collected. As a comparison, the obtained electrolyte using $Na^{14}NO_3$ as the feeding N-source and the fresh electrolytes before electrolysis were also subjected to the same ^1H NMR measurement.

1.11 Calculation of the yield rate, selectivity, conversion ratio, and Faradaic efficiency.

The yield rate of NH_3 was calculated using the formula (3):

$$\text{The yield rate of } NH_3 \ (r_{NH_3}) = \frac{C_{NH_3} \times V}{t \times A} \quad (3)$$

The selectivity of NH_3 was calculated using the formula (4):

$$\text{The selectivity of } NH_3 \ (S_{NH_3}) = \frac{C_{NH_3}}{C_0 - C_{NO_3^-}} \times 100\% \quad (4)$$

The conversion ratio of NO_3^- was calculated using the formula (5):

$$\text{The conversion ratio of } NO_3^- = \frac{C_0 - C_{NO_3^-}}{C_0} \times 100\% \quad (5)$$

The Faradaic efficiency for NH_3 production was calculated using the formula (6):

$$\text{The Faradaic efficiency for } NH_3 \text{ production } (FE_{NH_3}) = \frac{8 \times F \times C_{NH_3} \times V}{Q} \times 100\% \quad (6)$$

The turnover frequency for NH_3 production was calculated using the formula (7):

$$\text{The turnover frequency for } NH_3 \text{ production (TOF)} = \frac{C_{NH_3} \times V}{n_{Cu} \times t} \times 100\% \quad (7)$$

where C_{NH_3} (mol L^{-1}) is the measured concentration of NH_3, C_0 (mol L^{-1}) is the initial concentration of NO_3^-, $C_{NO_3^-}$ (mol L^{-1}) is the measured concentration of NO_3^- after electrolysis, V (L) is the volume of electrolyte in the cathode compartment, t (h) is the electrolysis time, A (cm^2) is the area of the modified electrode, F (C mol^{-1}) is the Faraday constant, Q (C) is the total charge applied for electrolysis, n_{Cu} (mol) is the molar quantity of Cu element on the electrode, which is calculated based on the Cu content and the catalyst loading; n_{Cu} for modified electrodes of Cu@MUiO-66(Ce) and Cu@UiO-66(Ce) are 1.075×10^{-6} and 6.514×10^{-7}, respectively.

2. Computational methods.

2.1 DFT calculations

To investigate the binding of Cu^{2+} to the cluster of MOFs, we employed a cluster model as done in the literature.[11] This cluster, consisting of a $[Ce_6O_4(OH)_4]$ node and benzoate groups (substituting BDC linkers), was extracted from the optimized crystal structure of UiO-66(Ce) done at the PBE-D3(BJ) level of theory within the Vienna Ab initio Simulation Package (VASP).[12] The projector-augmented wave (PAW) method was employed with a kinetic energy cutoff of 450 eV.[13] The lattice constant of UiO-66(Ce) was found to be 21.590 Å, being in good agreement with the experimental data (21.472 Å).[2]

Starting from the cluster, we removed one or two benzoate groups and substituted a pair of −OH/−OH_2 for each missing linker.[14] The complexes between a Cu^{2+} ion and the defective clusters were also optimized at the PBE-D3(BJ) level of theory. All para-carbon atoms of benzoate groups were fixed during the optimization to preserve the cluster's structure.[15] The Stuttgart/Dresden (SDD) and 6-31G(d,p) basis sets were employed for the metal centers and the remaining atoms, respectively.[16] Frequency calculations were performed to verify the characteristics of the optimized structures and obtain the quasi-harmonic free energy corrections (with a cutoff of 100 cm^{-1}).[17] On top of the optimized structures, single-point calculations at the B3LYP-D3(BJ)/def2-TZVP level of theory were done.[18] The SMD solvation model with the solvent-excluded surface (SES) was used in order to account for the water environment.[19] A correction term of 1.89 kcal/mol was added to the computed solvation free energy to convert from p = 1 atm standard state to 1 M standard state. All calculations were carried out using the Gaussian 16 and Turbomole 7.6 software packages.[20] The DDEC6 atomic population analysis calculations were performed with the chargemol program.[21]

2.2 Molecular dynamics simulations

Molecular dynamics (MD) simulations were used to investigate the diffusion of NO_3^- and NH_3 within the pores of UiO-66(Ce). All calculations were performed with the LAMMPS software package.[22] Four models (M1−M4) were constructed to represent varying pore sizes. The base model (M1) was built by cleaving a (001) slab (~40 Å thick) from the defect-free UiO-66(Ce) structure. All dangling bonds created by the cleave operation were capped with hydrogen. Two vacuum regions (~20 Å each) were added on either side of the slab, resulting in a tripartite simulation box with the bottom (z = 0−20 Å), middle MOF (z = 20−60 Å), and top (z = 60−80 Å) regions. This model maintains all linkers and has a dimension of 21.59 × 21.59 × 79.62 Å³. Model M2 was created from M1 by introducing defects as described in the work of Liu et al.[23] In M2, all missing linkers were substituted with −OH. To model a larger cavity, we constructed model M3 with an extended

unit cell 43.18 × 43.18 × 79.62 Å³. The cavity size in M3 is around 1/3 of that found in MUiO-66(Ce) (117 Å). To represent this mesoporous cavity, we used the M4 model with an empty unit cell with the same dimension as M1 and M2. All slabs were then solvated with equilibrated TIP4P water reservoirs. NO_3^- and Na^+ ions as well as NH_3 molecules were subsequently introduced in the bottom region at a concentration of ~4.5 x10^{-3} particles/Å³ (or 7.4 M). Water molecules overlapping with MOF and ions/molecules were deleted using a cutoff value of 1.5 Å. Finally, a graphene barrier was placed at the bottom of the simulation box to prevent the ions/molecules from directly entering the top region without diffusing through MOF. This graphene layer remained immobile during the simulations.

For MOFs, we used the UFF4MOF force field,[24] generated with lammps-interface code.[25] Repeating Electrostatic Potential Extracted ATomic (REPEAT) partial atomic charges were assigned using the PACMAN model.[26] OPLS-2009IL and OPLS-AA force field parameters were used for NO_3^-, Na^+, and NH_3,[27] whereas the TIP4P force field was used for water.[28]

The simulations employed periodic boundary conditions in all directions. After model construction, all systems underwent energy minimization followed by an equilibration step for 30 ps, using the microcanonical ensemble (NVE) and Berendsen thermostat (T = 300 K).[29] The *lj/cut/tip4p/long* pairstyle with a cutoff of 12 Å was used.[30] The bond lengths and angle in H_2O were constrained using the SHAKE algorithm, allowing a larger timestep of 1 fs.[31] Finally, 60 ns (for NO_3^-) and 30 ns (for NH_3) production runs were performed at 300 K in the canonical ensemble (NVT) with the Nosé-Hoover thermostat.[32] The trajectories were saved every 2000 steps (2 ps) and analyzed with the mdanalysis toolkit.[33]

3. Supplemental figures and tables

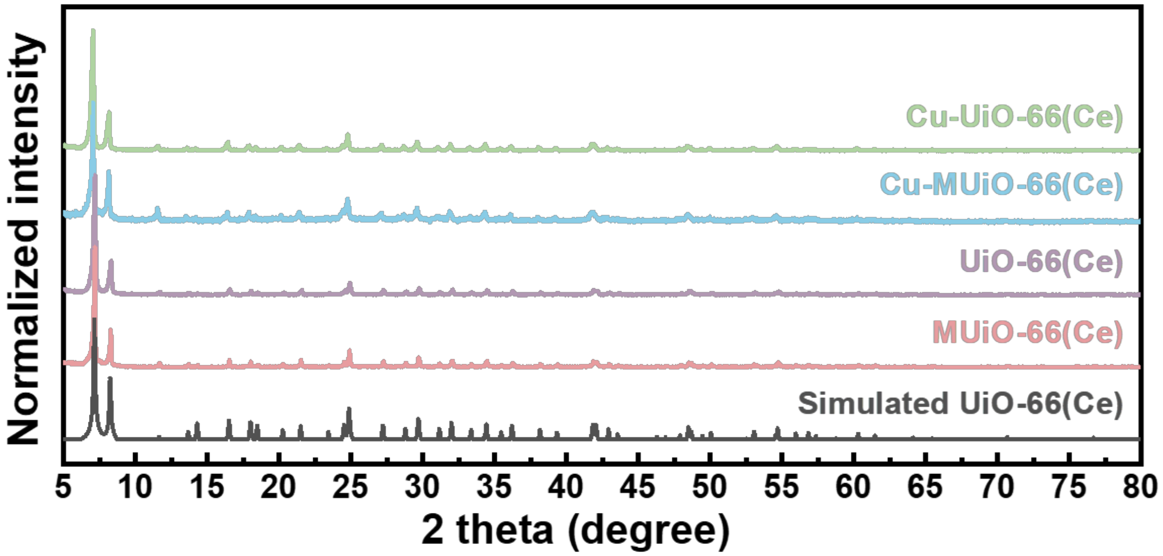

Figure S1. XRD patterns of MUiO-66(Ce), UiO-66(Ce), Cu-MUiO-66(Ce), and Cu-UiO-66(Ce) in the wide-angle range. The simulated pattern of UiO-66(Ce) is also shown.

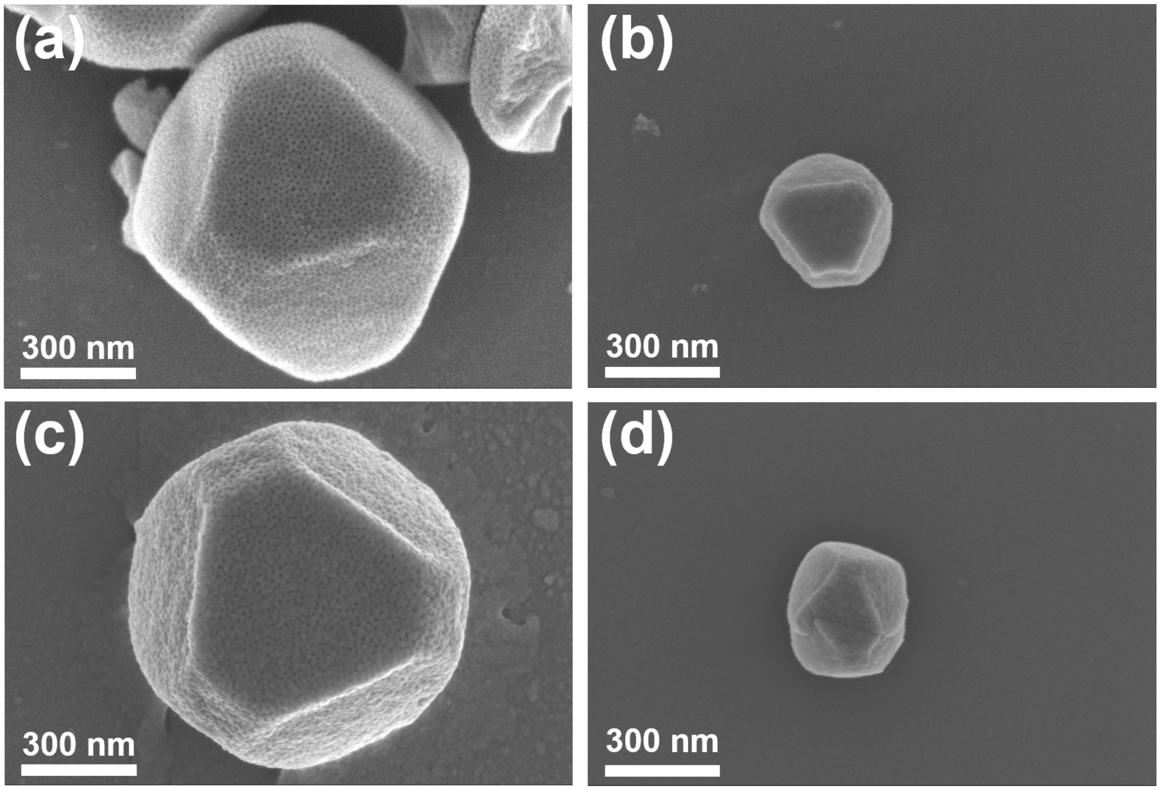

Figure S2. High-magnification SEM images of (a) MUiO-66(Ce), (b) UiO-66(Ce), (c) Cu-MUiO-66(Ce), and (d) Cu-UiO-66(Ce).

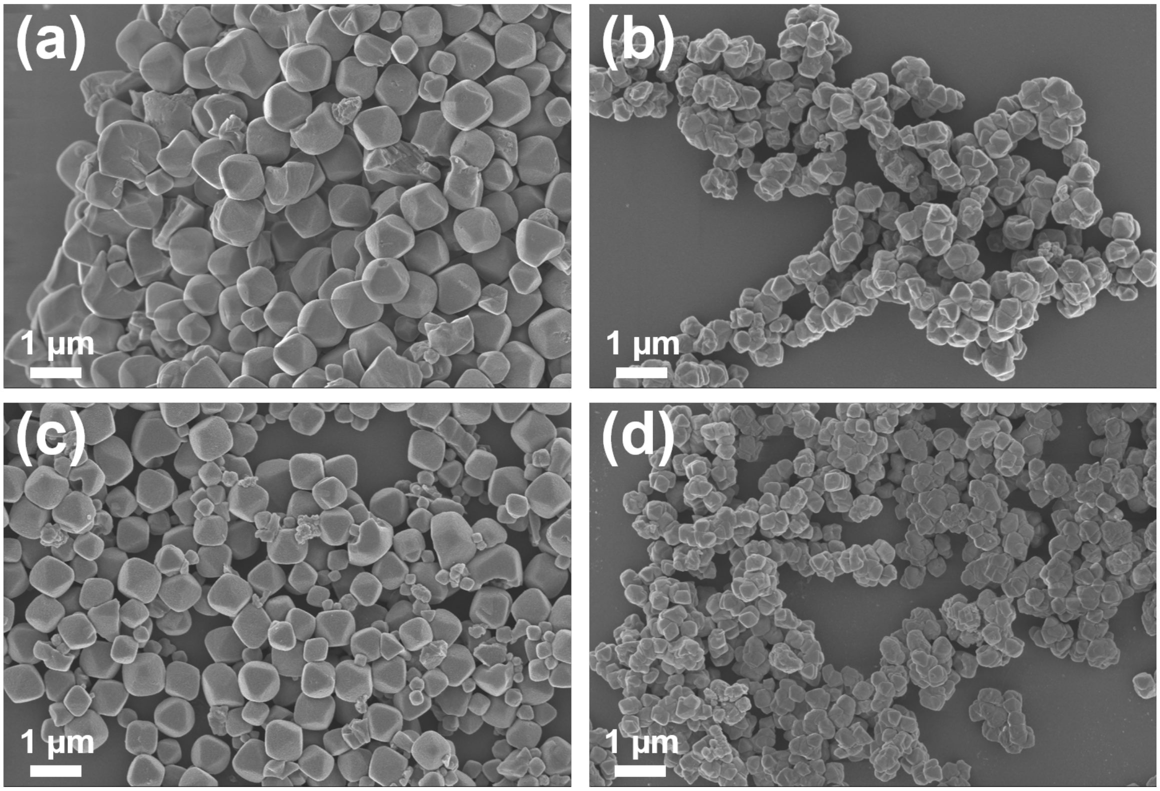

Figure S3. Low-magnification SEM images of (a) MUiO-66(Ce), (b) UiO-66(Ce), (c) Cu-MUiO-66(Ce), and (d) Cu-UiO-66(Ce).

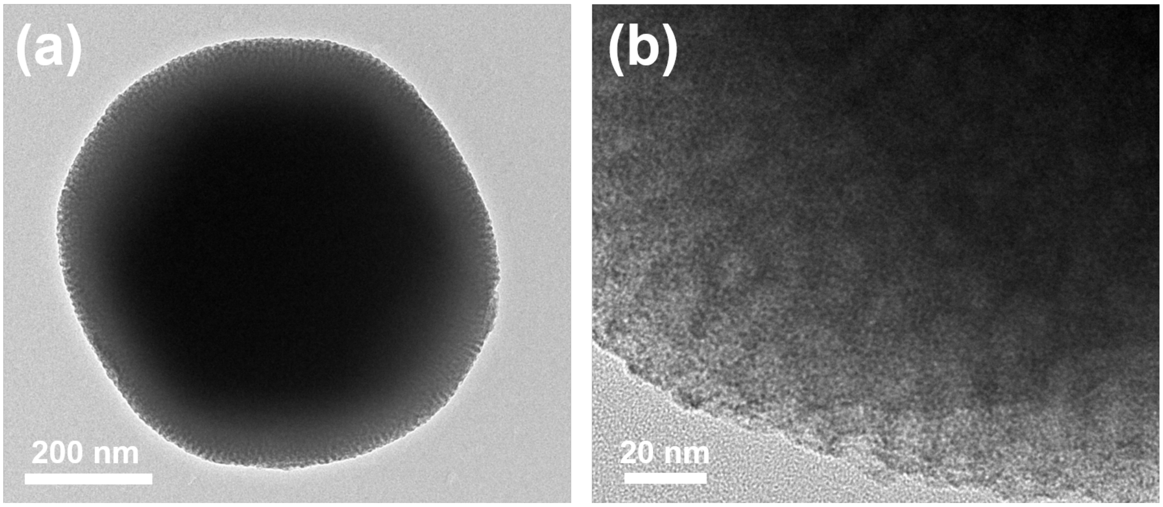

Figure S4. (a) TEM image and (b) HRTEM image of MUiO-66(Ce).

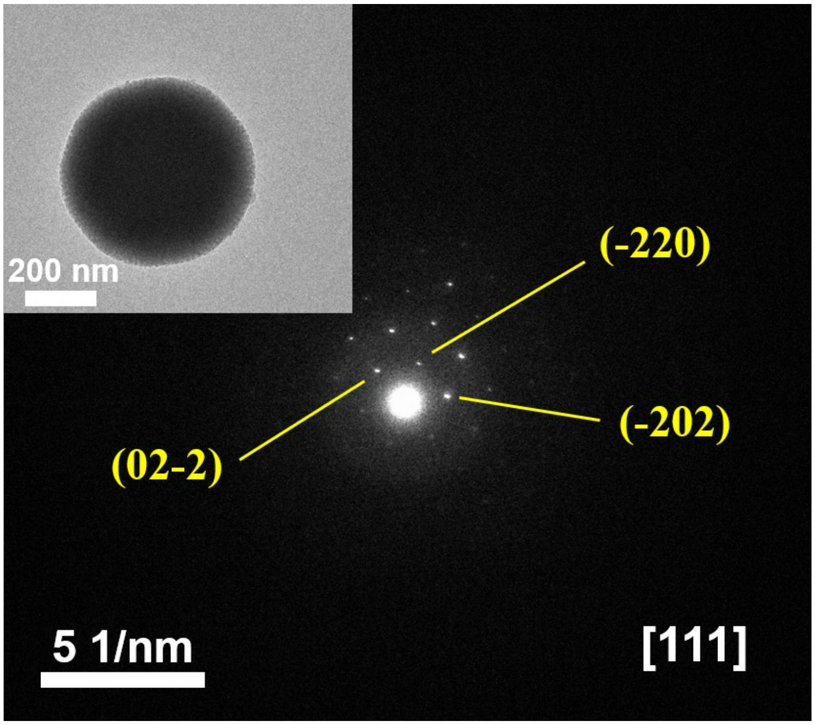

Figure S5. SAED pattern of MUiO-66(Ce) in [111] zone axis and the corresponding TEM image (inset).

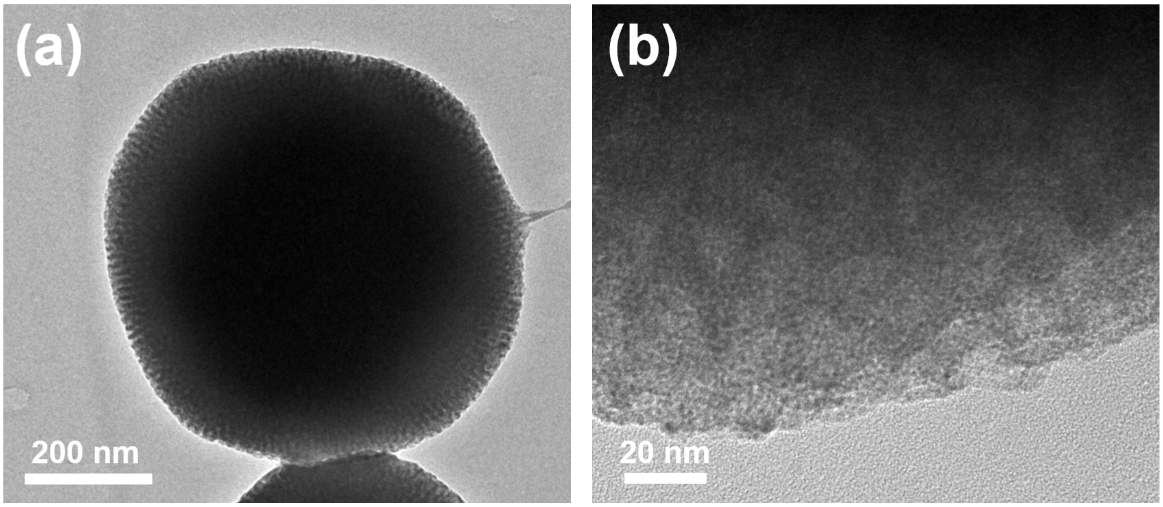

Figure S6. (a) TEM image and (b) HRTEM image of Cu-MUiO-66(Ce).

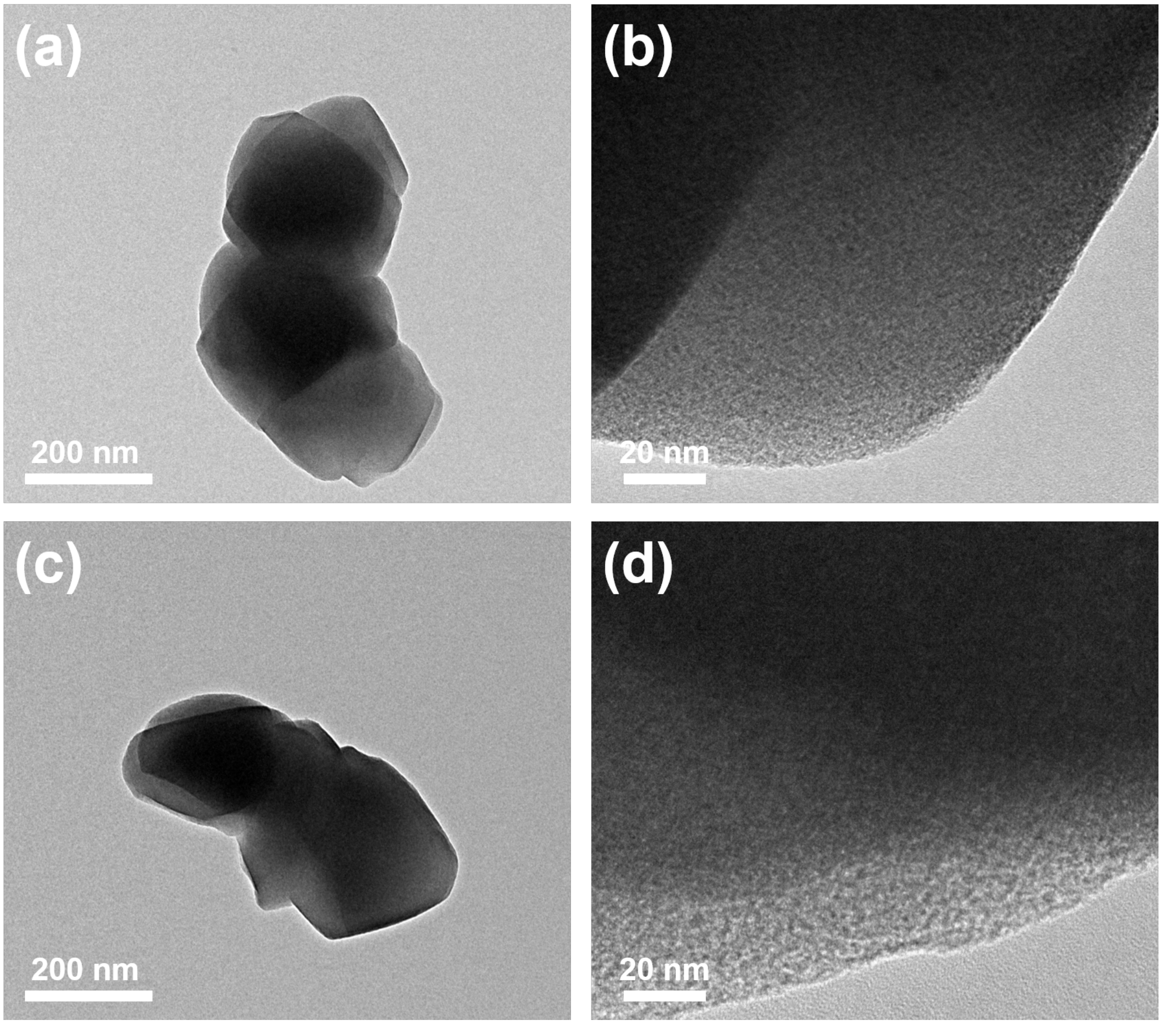

Figure S7. (a) TEM image and (b) HRTEM image of UiO-66(Ce). (c) TEM image and (d) HRTEM image of Cu-UiO-66(Ce).

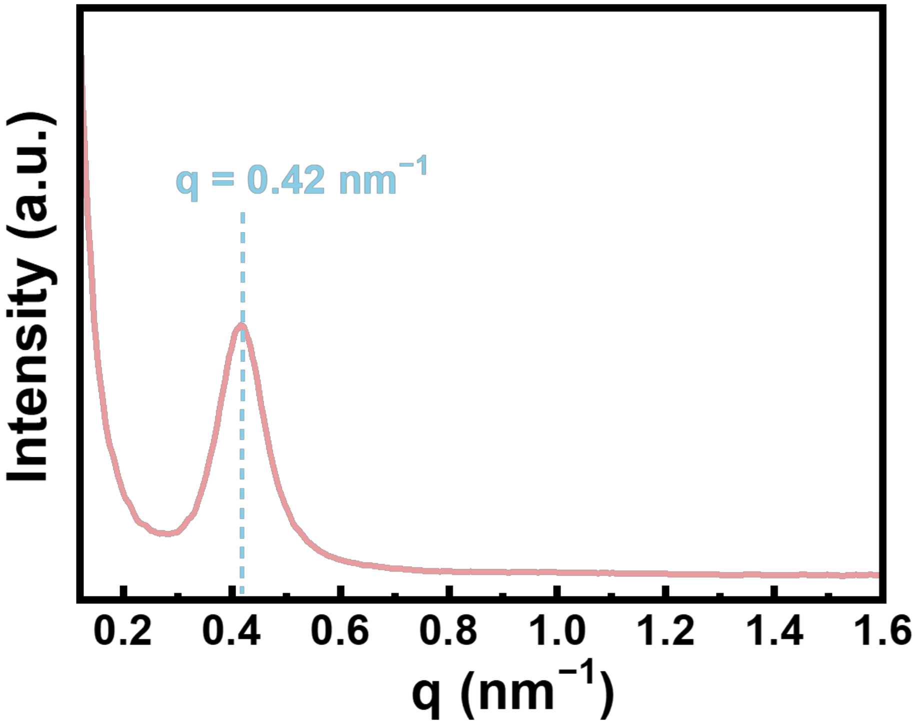

Figure S8. SAXS pattern of MUiO-66(Ce).

For determining the amount of −OH/−OH_2 pair present in the MOFs structure, each missing BDC linker is assumed to create two defective sites on the two neighboring hexa-cerium clusters, and each defective site is coordinated by either one −OH/−OH_2 pair or one monotopic modulator.[34] However, it was found that only the equivalence points with the p*K*a values of around 3.44 and 6.54 can be found for both UiO-66(Ce) and MUiO-66(Ce), which suggests that the defective sites present here are only coordinated by −OH/−OH_2 pairs. As a result, the molecular formula of the as-synthesized UiO-66(Ce) and MUiO-66(Ce) can be written as $Ce_6O_4(OH)_4(BDC)_{6-x}[(OH)(OH_2)]_{2x}$, where x is the number of missing linkers. From the difference in the amount of 0.1 M NaOH consumed between the first and the second equivalence points shown in Figure 2d-e, the amount of −OH/−OH_2 pair within 50 mg of titrated samples were calculated, and the results are listed at Table S1.

Table S1. Calculations and their respective results of acid−base titration experiments.

**Sample**	**Bridging −OH pK_a**	**Bridging −OH equivalence point (mL)**	**Ce−OH_2 pK_a**	**Ce−OH_2 equivalence point (mL)**	**−OH/OH_2 pair in 50 mg material (mmol)**	**x (Number of missing linkers)**	**Molecular weight**
UiO-66(Ce)	3.44	0.75	6.54	1.60	0.08500	1.54	1812.52
MUiO-66(Ce)	3.43	0.90	6.42	1.90	0.10000	1.79	1788.98

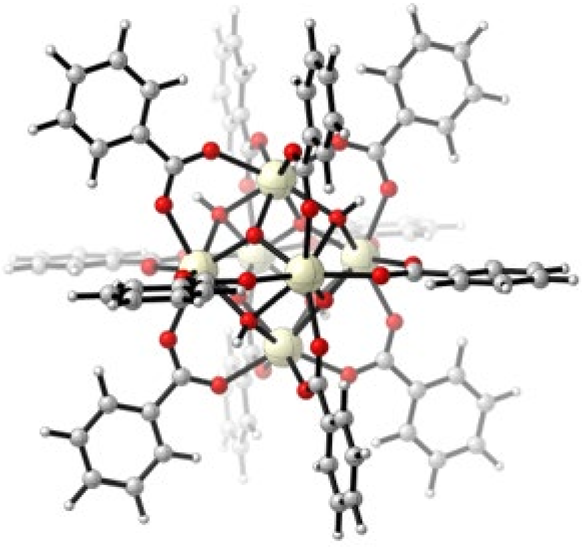

Figure S9. The $[Ce_6O_4(OH)_4](C_6H_5COO)_{12}$ cluster used in DFT calculations.

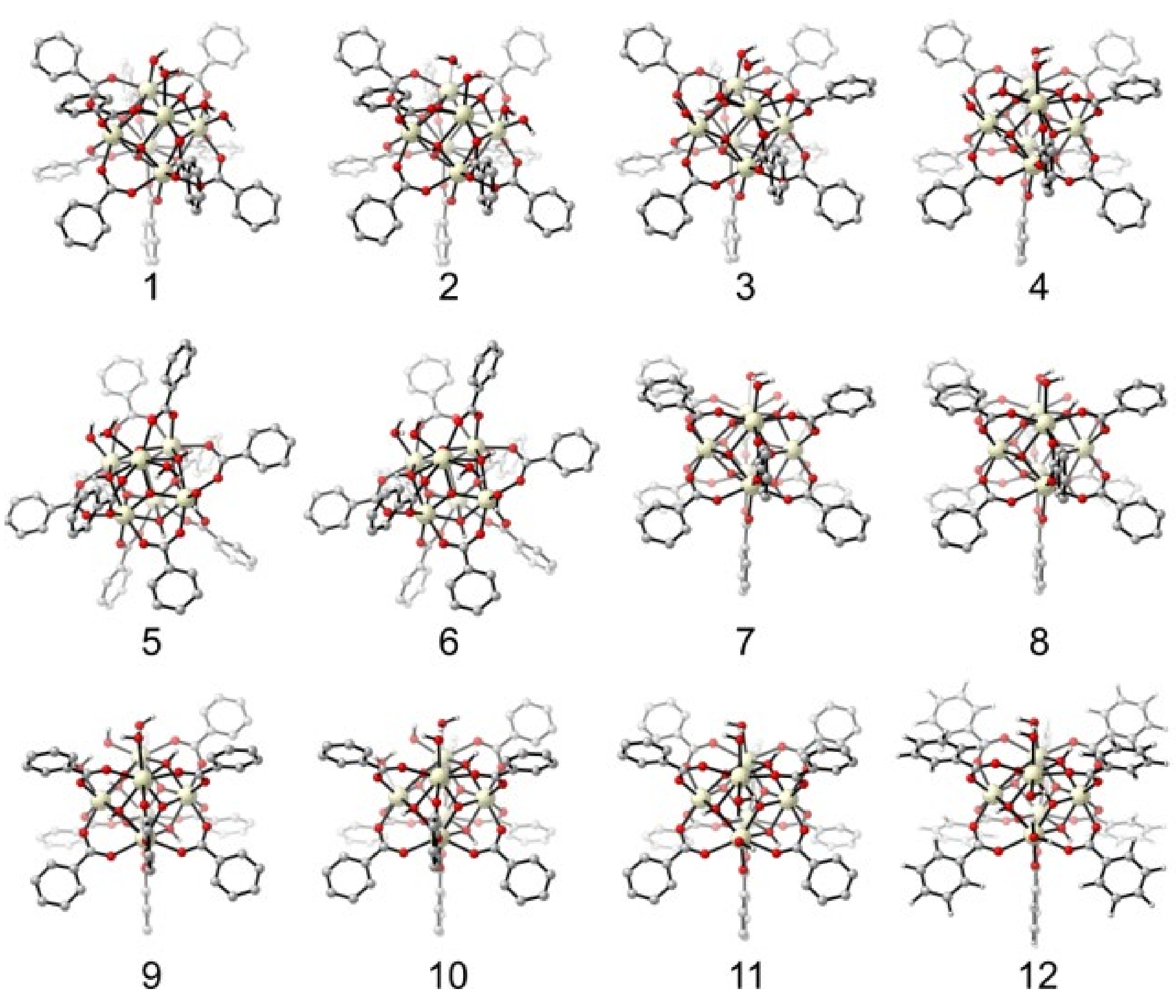

Figure S10. Twelve configurations of $D2[(OH)(OH_2)]_2$ calculated with DFT.

Table S2. Total energies (in E_h) of 12 configurations of D2[(OH)(OH_2)]$_2$ calculated with PBE/SDD/6-31G(d,p).

Configuration	E_{PBE}(gas)	Relative energy (kcal/mol)
1	−7957.577809	4.9
2	−7957.583932	1.0
3	−7957.578205	4.6
4	−7957.580119	3.4
5	−7957.585124	0.3
6	−7957.585587	0.0
7	−7957.580698	3.1
8	−7957.577441	5.1
9	−7957.582243	2.1
10	−7957.582811	1.7
11	−7957.585597	0.0
12	−7957.584320	0.8

Table S3. Total and Gibbs free energies (in E_h) of species calculated with PBE/SDD/6-31G(d,p) and B3LYP/def2-TZVP.

	E_{PBE}(gas)	G_{PBE}(gas)[a]	E_{B3LYP}(water)	G_{B3LYP}(water)[b]
D1(OH)(OH_2)	−8225.245848	−8224.182598	−8234.220637	−8233.154375
D2[(OH)(OH_2)]$_2$	−7957.580119	−7956.576879	−7966.128918	−7965.122666
H^+	0.000000	−0.010000	−0.423738[c]	−0.430726
Cu^{2+}	−196.223728	−196.240237	−1640.239374	−1640.252871
Cu^{2+}−D1(OH)(OH_2)	−8422.160798	−8421.101480	−9874.494323	−9873.431993
Cu^{2+}−D2[(OH)(OH_2)]$_2$	−8154.517663	−8153.513625	−9606.424418	−9605.417368
Cu^{2+}−D1$(OH)_2$	−8421.836185	−8420.783699	−9874.069827	−9873.014329
Cu^{2+}−D2$(OH)_3$(OH_2)	−8154.175237	−8153.182470	−9605.982820	−9604.987040

[a] Gibbs free energy corrections calculated with quasi-harmonic approximation proposed by Grimme.

[b] Gibbs free energy corrections taken from PBE/SDD/6-31G(d,p). The Gibbs free energies were corrected by +1.89 kcal/mol due to the conversion from p = 1 atm standard state to 1 M standard state.

[c] Energy of H^+ was chosen so that the hydration free energy of H^+ $\Delta G_{hyd}^{298}(H^+)$ is −265.9 kcal/mol.

The bulk electrical conductivity (σ) of each sample was then calculated by the following equation,

$$\sigma = \frac{l}{RA}$$

where R is the electrical resistance estimated from the slope of the I–V curve near 0 V in Figure S11, l is the thickness of the pellet, and A is the cross-section area of the pellet, which is 0.3848 cm^2. As shown in Figure S11, all pellets of the four MOFs only show current responses below the scale of 0.1 nA with significant noise, and the slopes can only be obtained by linearly fitting the noisy data. The obtained σ are listed in Table S4.

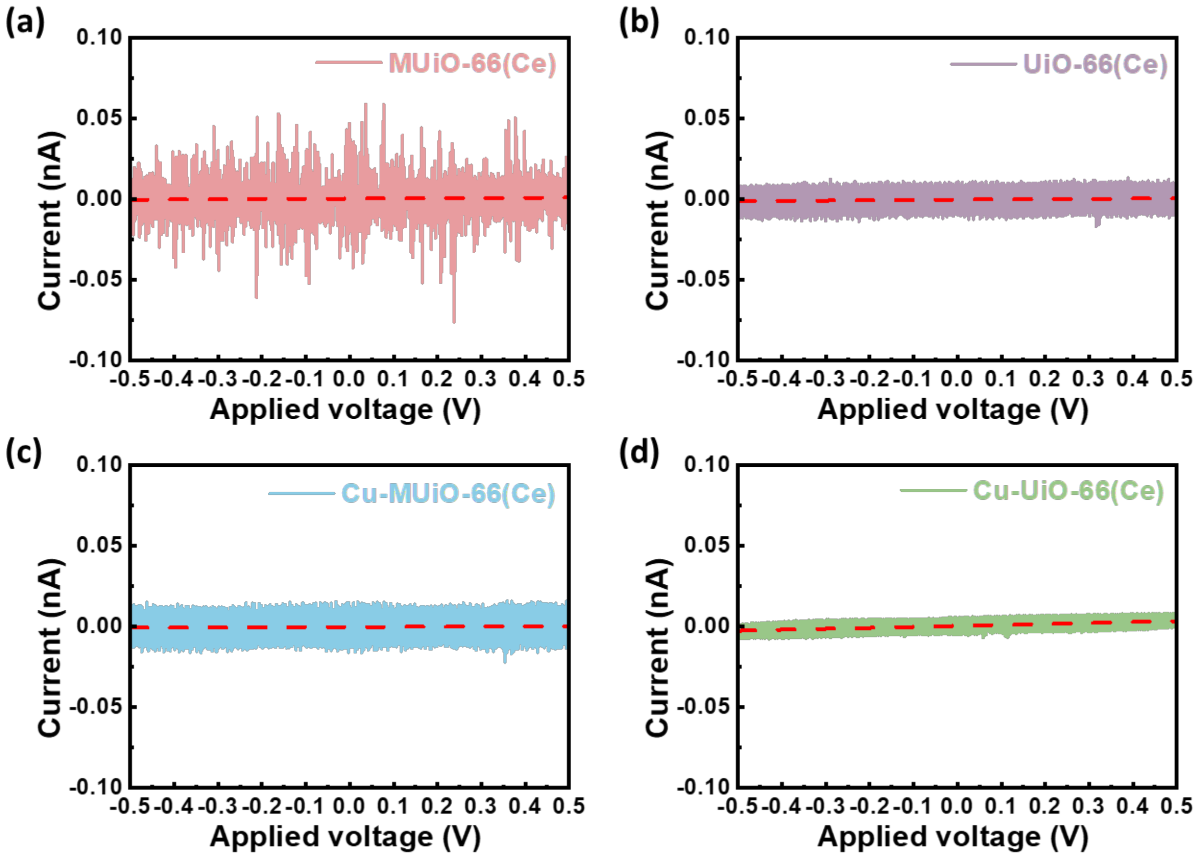

Figure S11. I–V curves of (a) MUiO-66(Ce), (b) UiO-66(Ce), (c) Cu-MUiO-66(Ce) and (d) Cu-UiO-66(Ce) pellets; the red dashed lines indicate the slope of the I–V curves for estimating the conductivity. All I–V curves were measured at a scan rate of 25 mV/s in air.

Table S4. Results calculated from the data shown in Figure S11.

	R (Ω)	*l* (cm)	*A* (cm^2)	Conductivity (S/cm)
MUiO-66(Ce)	7.09E+11	0.023	0.3848	8.43E-14
UiO-66(Ce)	6.10E+11	0.023	0.3848	9.80E-14
Cu-MUiO-66(Ce)	1.28E+12	0.019	0.3848	3.86E-14
Cu-UiO-66(Ce)	3.38E+11	0.021	0.3848	1.61E-13

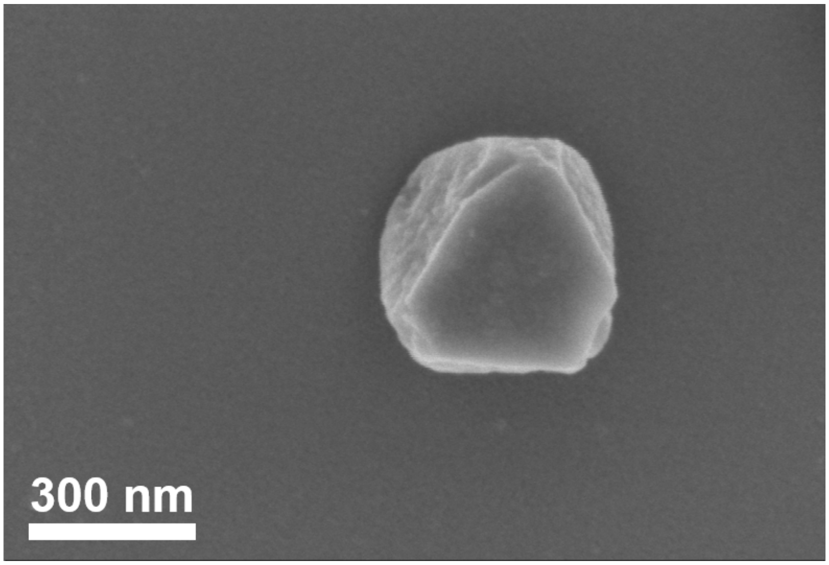

Figure S12. High-magnification SEM image of Cu@UiO-66(Ce).

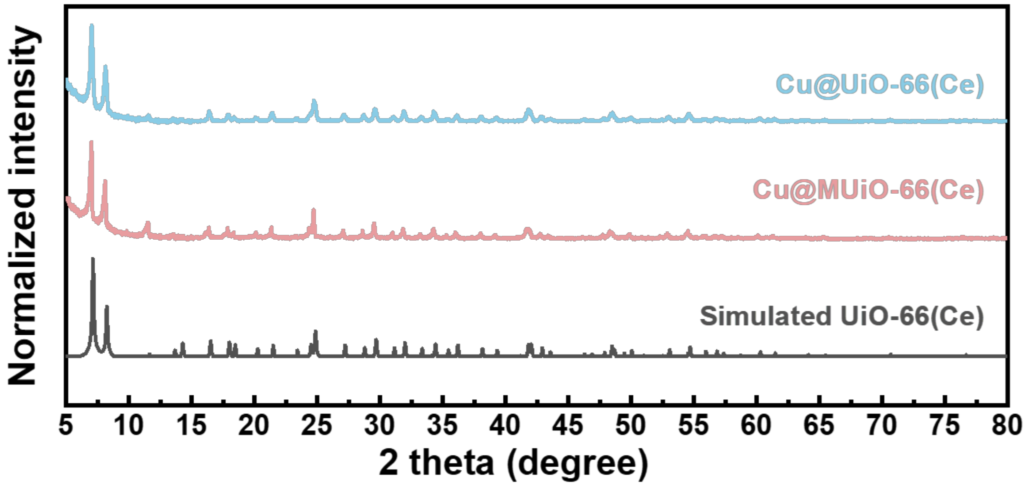

Figure S13. XRD patterns of Cu@MUiO-66(Ce) and Cu@UiO-66(Ce). The simulated pattern of UiO-66(Ce) is also shown.

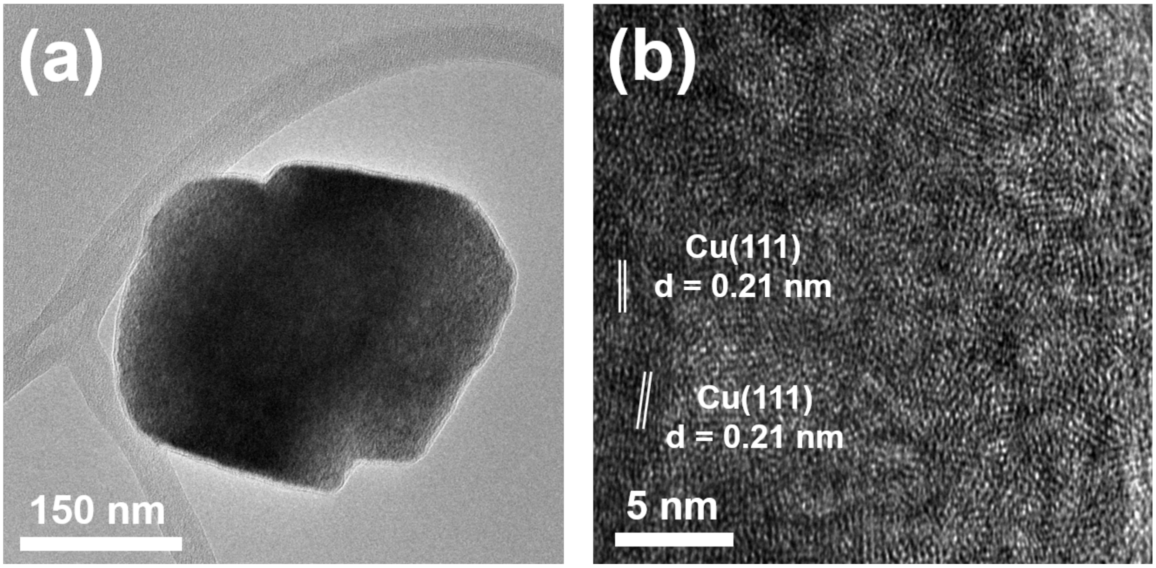

Figure S14. (a) TEM image and (b) HRTEM image of Cu@UiO-66(Ce).

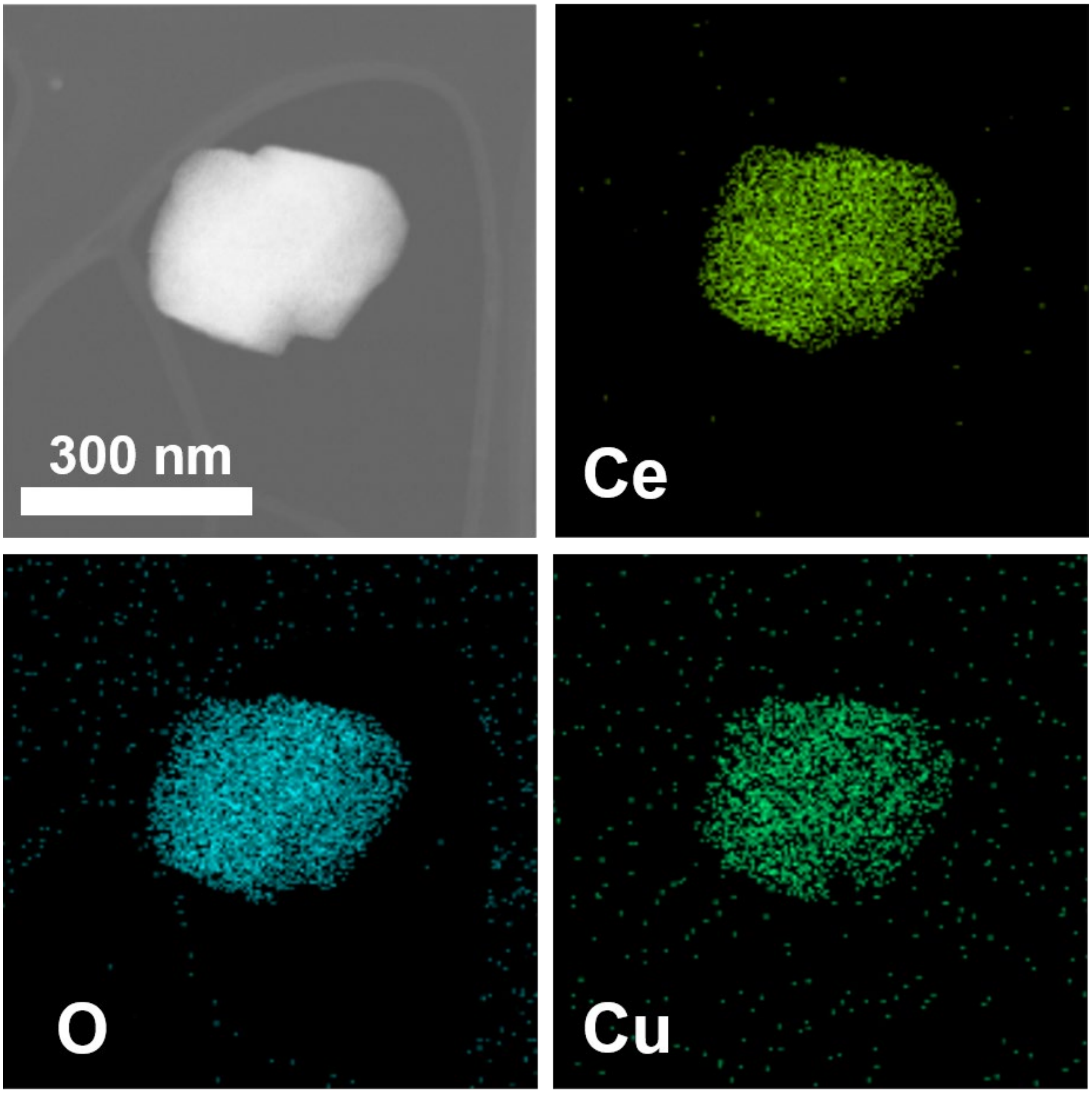

Figure S15. EDS elemental mapping data for Ce, O, and Cu of Cu@UiO-66(Ce).

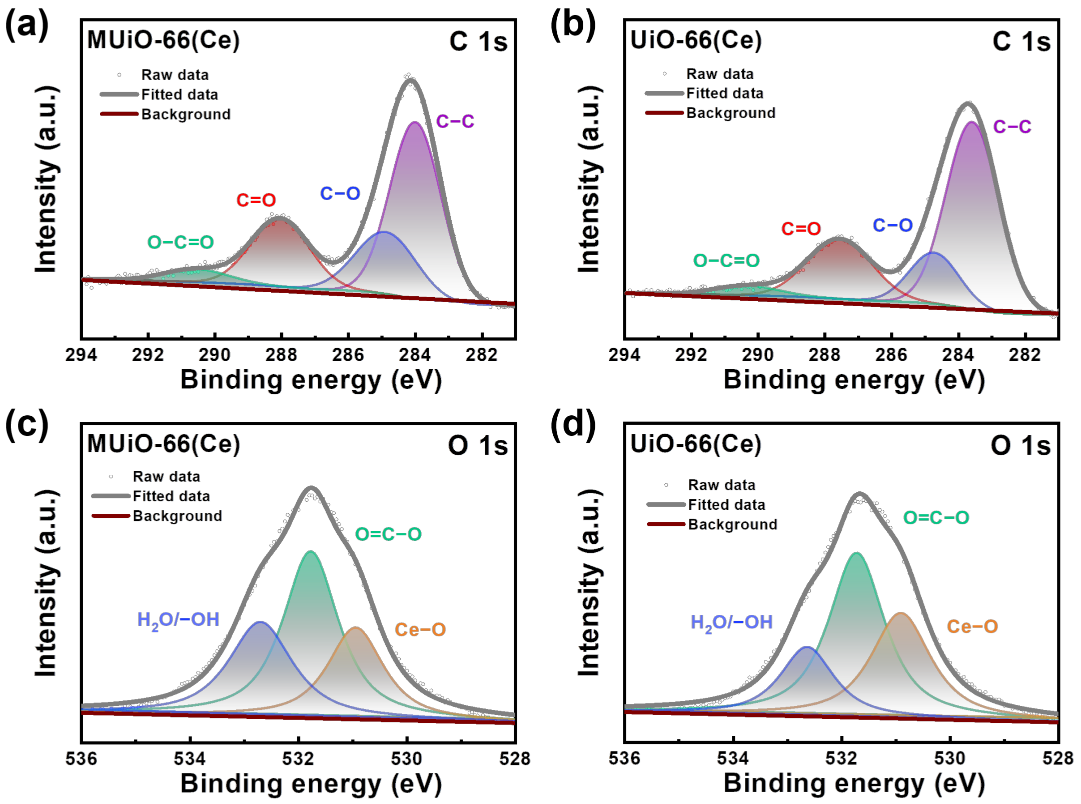

Figure S16. XPS spectra of the (a) MUiO-66(Ce) and (b) UiO-66(Ce), collected in the region of C 1s. XPS spectra of the (c) MUiO-66(Ce) and (d) UiO-66(Ce), collected in the region of O 1s.

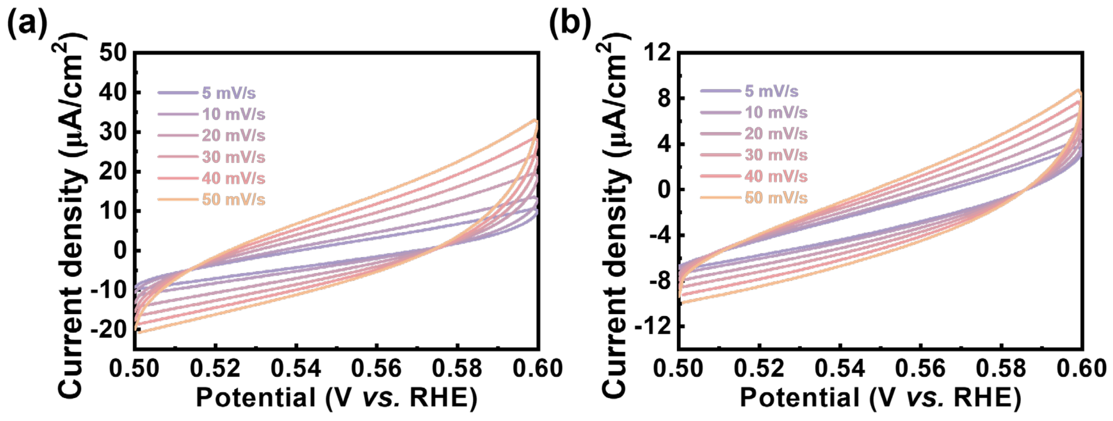

Figure S17. CV curves of the modified electrodes of (a) Cu@MUiO-66(Ce) and (b) Cu@UiO-66(Ce), collected at different scan rates from 5 mV/s to 50 mV/s.

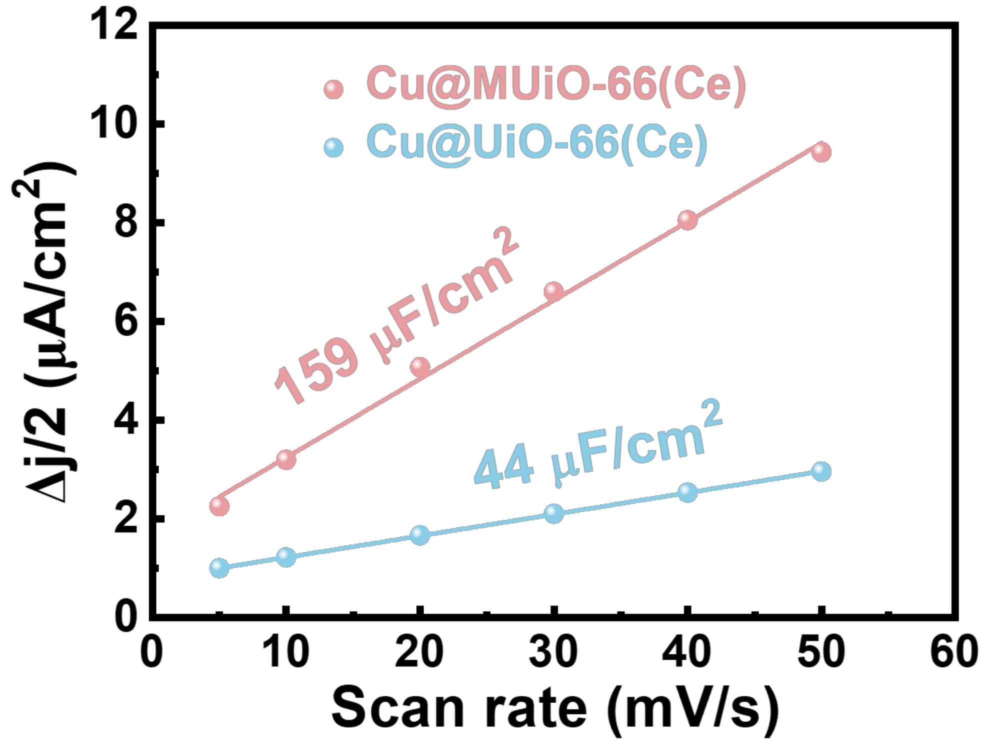

Figure S18. Plots of Δj/2 *vs*. scan rate, where Δj is the difference between the anodic and cathodic current density at +0.55 V *vs*. RHE in each CV curve extracted from the data shown in Figure S17.

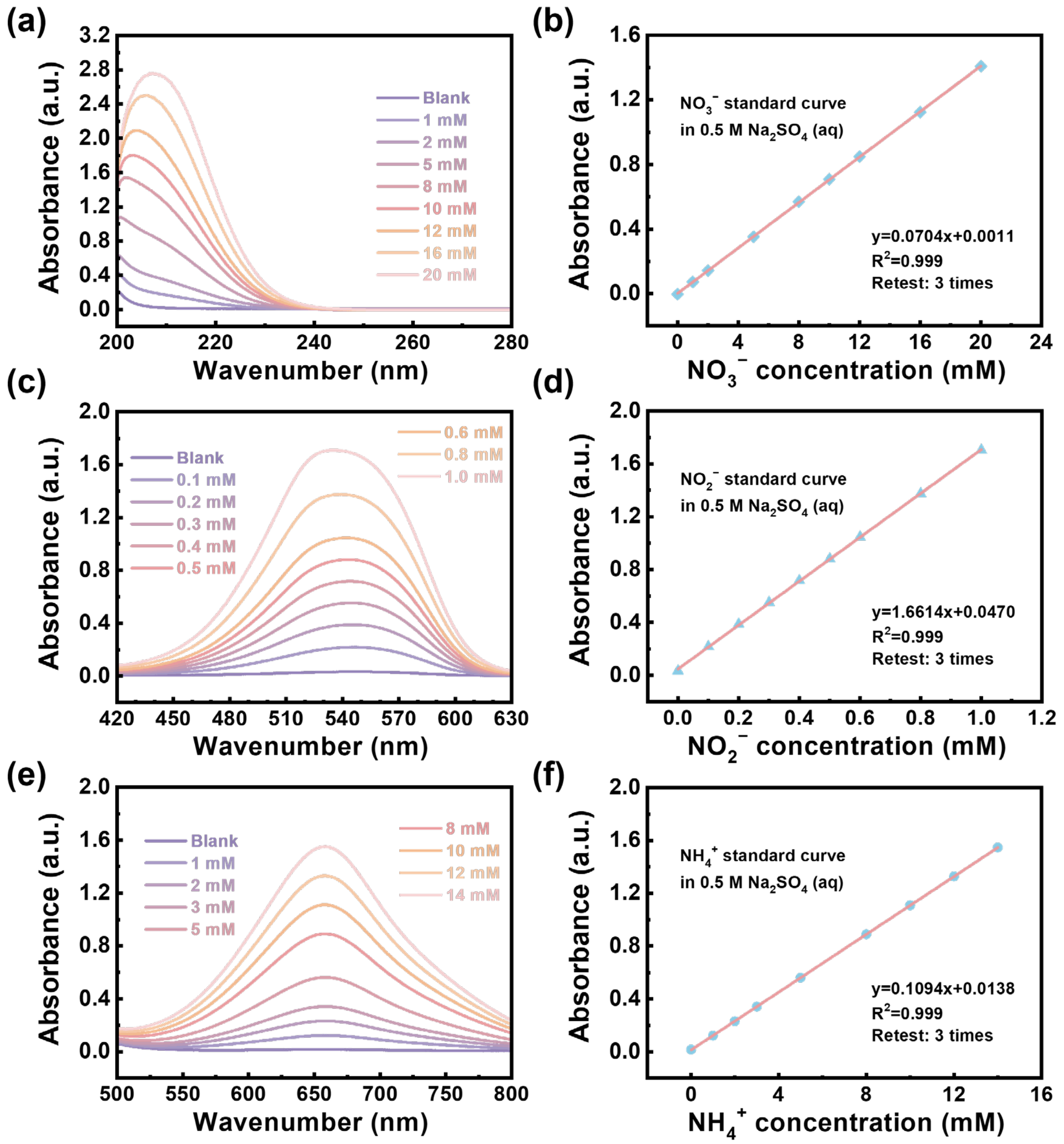

Figure S19. (a) The UV-visible spectra and (b) corresponding concentration calibration curve for the determination of nitrate in 0.5 M Na_2SO_4 aqueous solution. (c) The UV-visible spectra and (d) corresponding concentration calibration curve for the determination of nitrite in 0.5 M Na_2SO_4 aqueous solution. (e) The UV-visible spectra and (f) corresponding concentration calibration curve for the determination of ammonia in 0.5 M Na_2SO_4 aqueous solution.

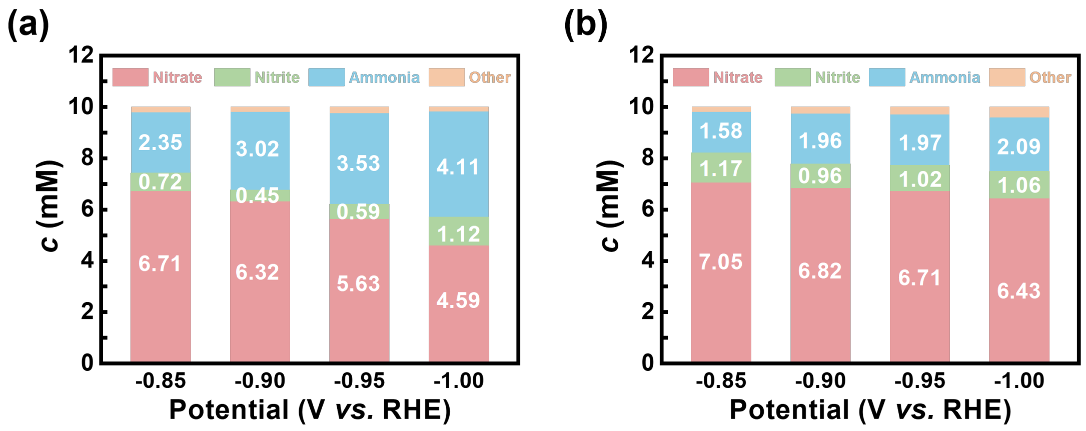

Figure S20. Concentrations of nitrate, nitrite, and ammonia in each electrolyte after electrolysis for 30 min at various applied potentials using (a) Cu@MUiO-66(Ce)-modified electrode and (b) Cu@UiO-66(Ce)-modified electrode.

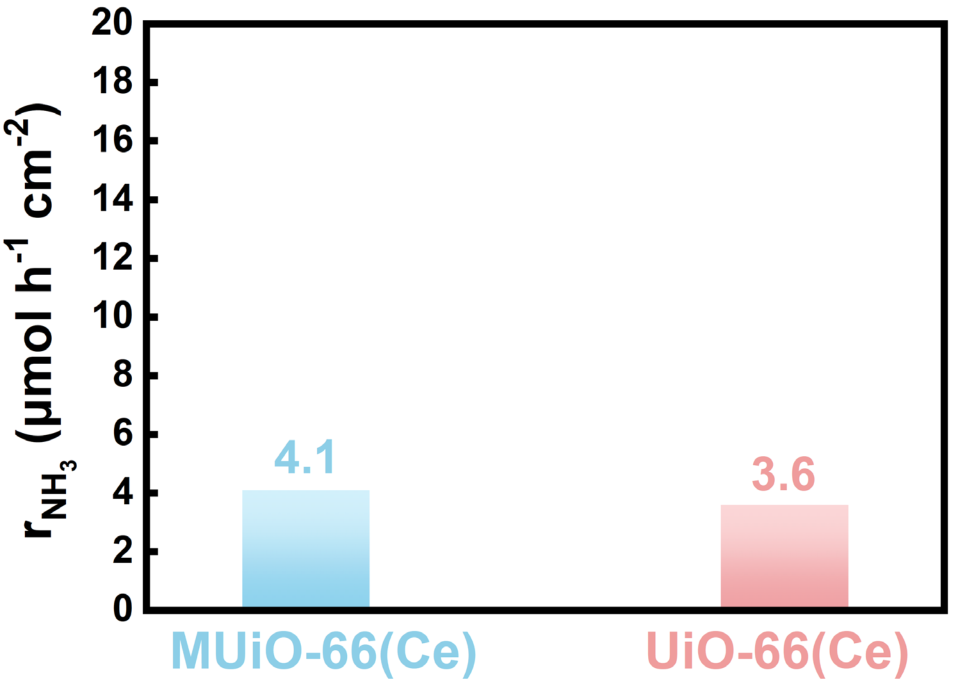

Figure S21. The r_{NH3} values achieved by MUiO-66(Ce)- and UiO-66(Ce)-modified electrodes at −0.95 V *vs.* RHE for 30 min.

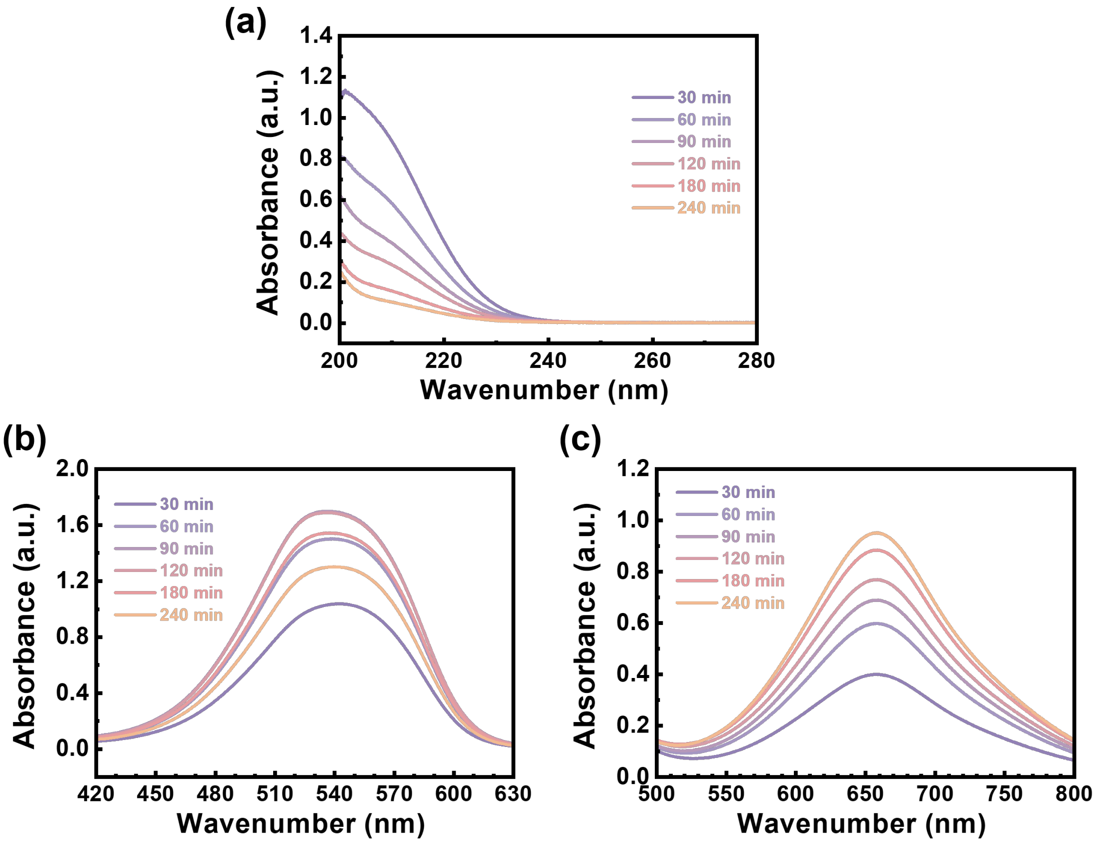

Figure S22. UV-visible spectra for determining the concentrations of (a) nitrate, (b) nitrite, and (c) ammonia in the electrolyte after electrolysis with the Cu@MUiO-66(Ce) electrocatalyst at −0.95 V *vs*. RHE for 30, 60, 90, 120, 180, and 240 min.

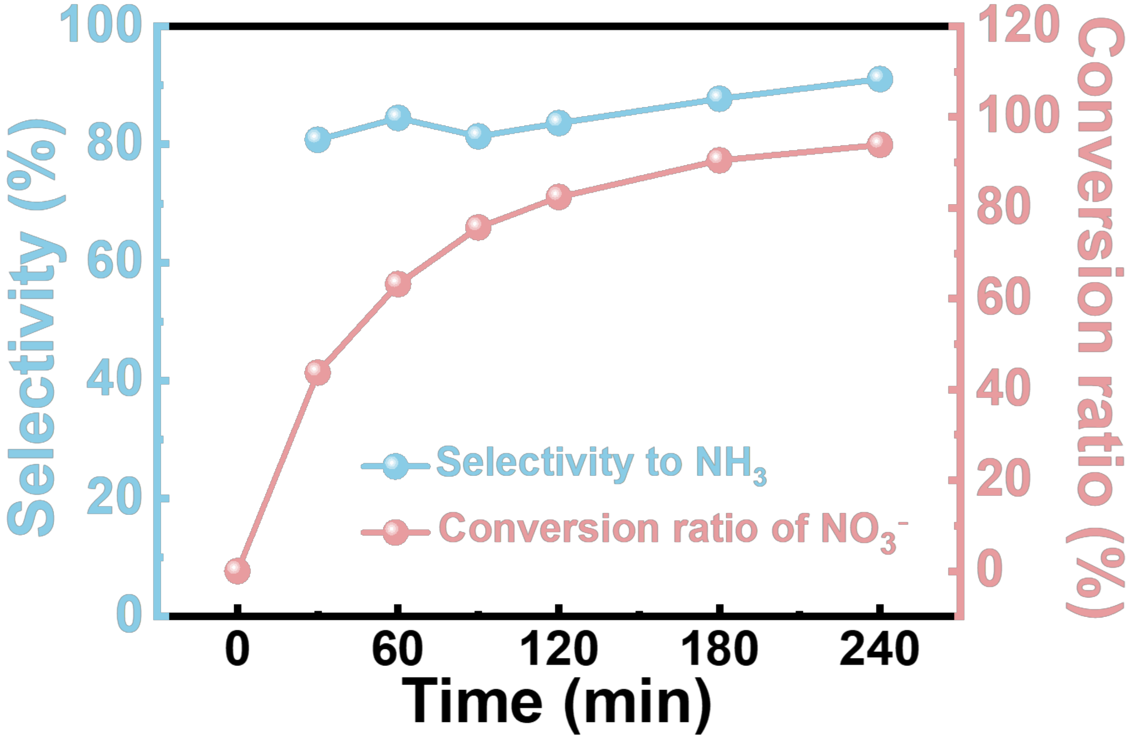

Figure S23. The selectivity for ammonia production and the conversion ratio of nitrate reduction during the electrolysis at −0.95 V *vs*. RHE for 4 h, extracted from the data shown in Figure 5c.

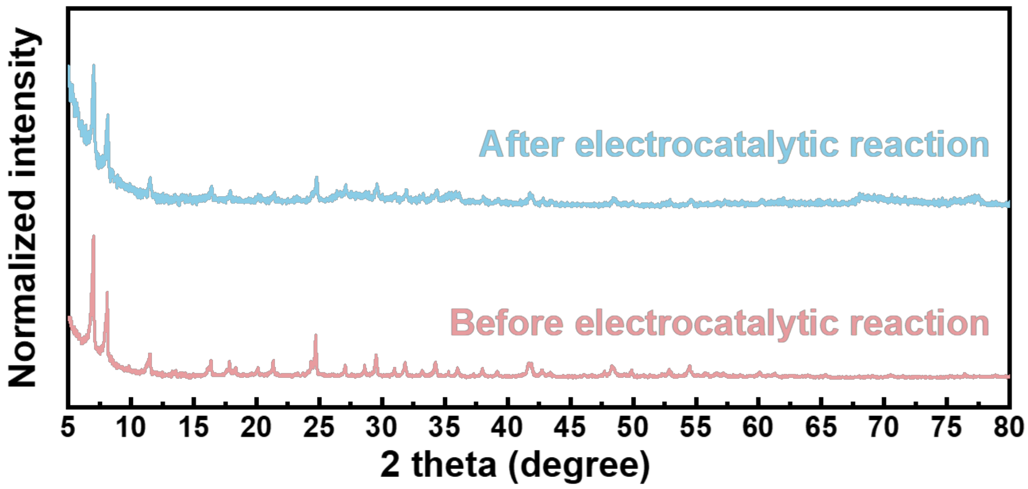

Figure S24. XRD patterns of Cu@MUiO-66(Ce) before and after electrolysis at −0.95 V *vs*. RHE for 4 h.

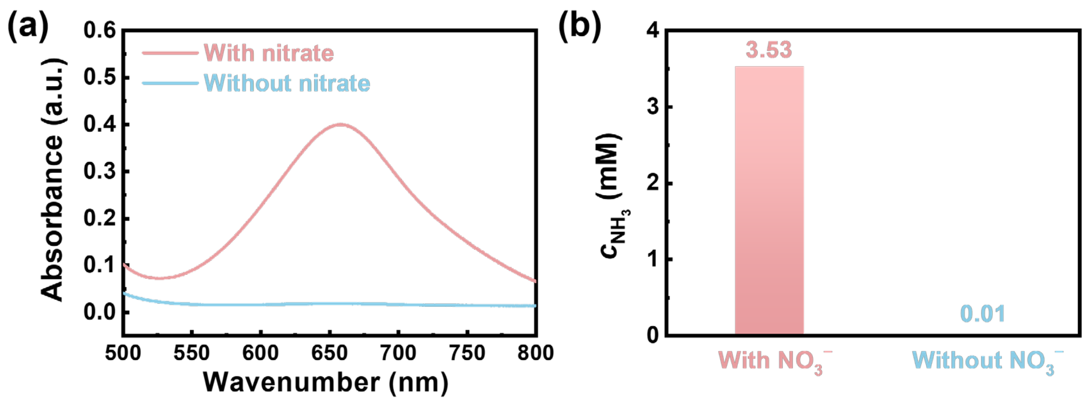

Figure S25. (a) The UV-visible spectra for determining the concentrations of ammonia in the electrolyte after electrolysis with and without 10 mM of nitrate. (b) Corresponding concentration values extracted from (a). The electrolysis was conducted with the Cu@MUiO-66(Ce)-modified electrode at −0.95 V *vs*. RHE for 30 min.

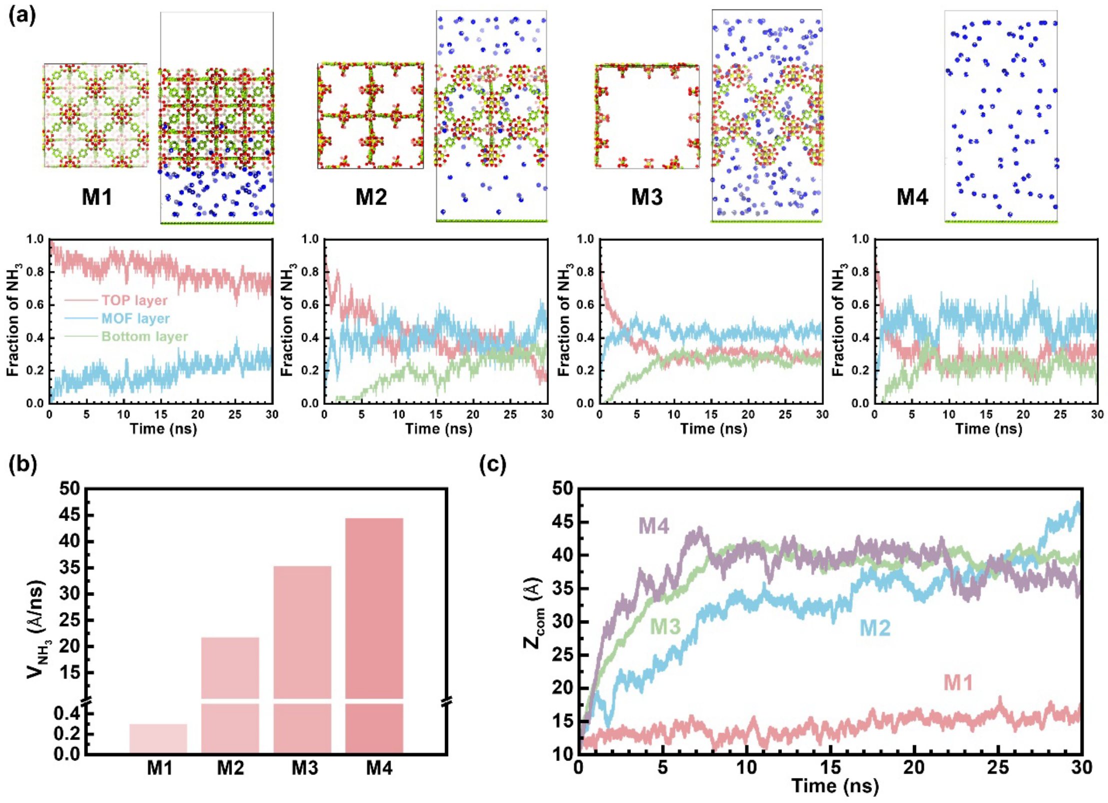

Figure S26. (a) Top and side views of the M1−M4 models. The side views are taken from snapshots at 30 ns. Yellow, red, green, blue, and white balls represent Ce, O, C, N, and H atoms, respectively. Hydrogen atoms of MOF and water molecules are omitted for clarity. The simulation cells of M1, M2, and M4 were duplicated to improve visibility. The subplots show the fraction of NH_3 molecules in each region (bottom, MOF, and top) throughout the simulations. (b) The z component of NH_3 velocity (in Å/ns) within the MOF region in M1−M4. (c) z-component of the center-of-mass (in Å) of NH_3 during the simulations in M1−M4 models.

Table S5. A partial list of Cu-based electrocatalysts for electrochemical nitrate reduction to ammonia with their electrocatalytic parameters, sorted according to the ammonia Faradaic efficiency.

Material	Electrolyte	NH_3 yield rate (mg h^{-1} mg_{cat}^{-1})	FE_{NH3} (%)	TOF (h^{-1})	Reference
Cu/Cu_2O NWAs [a]	200 ppm NO_3^- ($NaNO_3$) + 0.5 M Na_2SO_4	-	95.8	-	[35]
UiO-CuZn	200 ppm NO_3^- ($NaNO_3$) + 0.5 M Na_2SO_4	0.00388 [j]	91.4	36,180	[9]
Plasma-treated Cu_2O	50 ppm NO_3^- ($NaNO_3$) + 0.5 M Na_2SO_4	1.411 [j]	89.5	-	[36]
Cu@MUiO-66(Ce)	10 mM $NaNO_3$ + 0.5 M Na_2SO_4	1.875	88.7	98.48	This work
Cu@Cu_{2+1}O NWs [b]	50 ppm NO_3^- (KNO_3) + 0.5 M K_2SO_4	0.577	87.07	-	[37]
O-Cu−PTCDA [c]	500 ppm NO_3^- (KNO_3) + 0.1 M PBS [i]	0.033 [j]	85.9	-	[38]
Cu nanotubes	50 ppm NO_3^- (KNO_3) + 0.5 M K_2SO_4	0.7786	85.7	-	[39]
1-Cu’	5 mM $NaNO_3$ + 0.5 M Na_2SO_4	1.122 [j]	85.5	219.2	[4]
Defect-rich metallic Cu nanoplates	50 ppm NO_3^- (KNO_3) + 0.5 M K_2SO_4	0.78125	85.47	-	[40]

Cu−N−C SAC [d]	0.1 M KNO_3 + 1 M KOH	3.375 [j]	84.7	-	[41]
Cu@Cu_2O MSs [e]	500 ppm NO_3^- ($NaNO_3$) + 0.1 M Na_2SO_4	5.569 [j]	80.57	-	[42]
pCuO-5 [f]	0.05 M KNO_3 + 0.05 M H_2SO_4	9.92 [j]	80	-	[43]
Cu SAGs [g]	20 mM $NaNO_3$ + 0.1 M PBS [i]	1.1 [j]	78	-	[44]
Cu@C	1 mM KNO_3 + 1 M KOH	0.4695	72	-	[45]
Cu@CuHHTP	500 ppm NO_3^- ($NaNO_3$) + 0.5 M Na_2SO_4	3.68 [j]	67.55	-	[46]
CuCl_BEF [h]	100 ppm NO_3^- (KNO_3) + 0.5 M Na_2SO_4	1.655 [j]	44.7	64.4	[47]

[a] Cu/Cu_2O NWAs: Cu/Cu_2O nanowire arrays

[b] Cu@$Cu_{2+1}O$ NWs: Cu@$Cu_{2+1}O$ core-sheath nanowires

[c] O-Cu−PTCDA: 3,4,9,10-perylenetetracarboxylic dianhydride with an optimum content of Cu

[d] Cu−N−C SAC: Cu ions doped into porous nitrogen-doped carbon single-atom catalysts

[e] Cu@Cu_2O MSs: Cu@Cu_2O microspheres

[f] pCuO-5: plasma-treated CuO

[g] Cu SAGs: Cu single-atom gel

[h] CuCl_BEF: CuCl-based catalyst with a built-in electric field

[i] PBS: phosphate buffer solution

[j] Values reported in different units and calculated by us

4. Cartesian coordinates

D1(OH)(H_2O)

H	-1.9411453	-3.0226102	-0.2968878
O	-1.4194372	-2.2023893	-0.3270065
H	-7.6397401	-3.5191917	-3.8690170
C	-6.5733510	-3.6839350	-4.0470582
H	-2.7675256	-4.2767151	-4.6919932
C	-3.8378763	-4.1271204	-4.5309693
O	-2.0018397	-2.5583044	-3.1515937
H	-6.0939276	1.6014848	-2.6991425
C	-5.8694891	2.4297005	-3.3757219
O	-3.8130037	1.2746574	-1.8864525
H	-4.9442482	5.3882870	-5.7567363
C	-5.1939291	4.5573330	-5.0909373
O	-0.1446138	1.3911326	-4.4162139
H	-7.9145610	2.7807486	-3.9957860
C	-6.8695014	3.0876695	-4.0946248
H	-3.1412088	4.1845277	-4.4651209
C	-4.1915286	3.8957849	-4.3800405
O	-2.2512270	2.4660635	-3.0244801
H	-4.4865782	-5.6261600	-5.9527054
C	-4.7948112	-4.8711205	-5.2239094
H	-5.9004584	-2.1872102	-2.6114620
C	-5.6186212	-2.9423948	-3.3490030
O	-3.6532032	-1.6063430	-1.9161589
O	-0.1084414	-1.1711376	-4.5758881
C	-3.2337393	-2.3840112	-2.8336669
C	-3.4572800	2.1398520	-2.7571811
C	-4.2515707	-3.1654065	-3.5922513
C	-6.5257900	4.1481500	-4.9444200
C	-4.5315900	2.8348693	-3.5214528
C	-6.1559600	-4.6454300	-4.9774900
O	-1.0738311	-0.0644302	-1.8451498
H	-7.9671374	2.8059210	3.2339483
C	-6.9230926	3.0905265	3.3933595
H	-3.1954390	4.0912011	3.9687249
C	-4.2461134	3.8290515	3.8231375
O	-2.2698344	2.4913976	2.3866394
H	-4.5689517	-5.6241870	5.2915817
C	-4.8730085	-4.8933513	4.5366640

H	-5.9558259	-2.2794945	1.8469269
C	-5.6779625	-3.0172786	2.6035136
O	-3.6955491	-1.6356973	1.2192821
H	-0.1617901	-2.1820514	6.0660921
C	-0.1869197	-1.2537370	6.6420609
O	-0.0805101	-1.1552628	3.8609676
H	-0.2780388	2.1201235	8.6045928
C	-0.2522619	1.1776893	8.0499145
H	-7.7033204	-3.5891920	3.1102266
C	-6.6390995	-3.7466095	3.3076365
H	-2.8408279	-4.3034424	4.0144874
C	-3.9095649	-4.1658878	3.8338241
O	-2.0545790	-2.7119154	2.3578383
H	-6.1182250	1.6464534	1.9730304
C	-5.9038219	2.4419575	2.6909044
O	-3.8162149	1.2742563	1.2579757
H	-5.0278066	5.2722054	5.2344776
C	-5.2678150	4.4786148	4.5209695
H	-0.2015423	-2.2062388	8.5856876
C	-0.2087212	-1.2586037	8.0393239
H	-0.2425759	2.1188824	6.0838705
C	-0.2324499	1.1857947	6.6526223
O	-0.1641066	1.1114892	3.8658514
C	-3.4735970	2.1410876	2.1337982
C	-3.2834377	-2.4673998	2.0980147
C	-0.1506323	-0.0226457	4.4538130
C	-4.5637420	2.8134542	2.9029698
C	-6.2334700	-4.6834100	4.2690900
C	-0.1961126	-0.0306522	5.9471478
C	-4.3123829	-3.2296572	2.8647013
C	-6.6033500	4.1101600	4.3021400
C	-0.2379800	-0.0436700	8.7395900
O	-1.1128549	-0.0645205	1.2549632
H	-2.1786233	2.8240101	-0.2937539
O	-1.5934815	2.0468617	-0.3087624
Ce	-2.6700827	-0.1140527	-0.3164870
H	3.1873161	-4.1376133	-4.5072316
C	4.2402669	-3.9536571	-4.2812572
O	2.2634622	-2.6173387	-2.8565875
H	7.9710353	-3.2217425	-3.3856147
C	6.9240521	-3.4197615	-3.6322187
H	4.9347818	-5.4747732	5.1260890

C	5.1858337	-4.6989537	4.3970772
H	6.0810263	-1.9334084	1.7915838
C	5.8560081	-2.7105507	2.5259188
O	3.7865799	-1.4054385	1.2060577
H	0.4488375	-8.8794812	-2.4776807
C	0.4531457	-8.3238055	-1.5355691
H	0.4723450	-6.3411469	1.8276647
C	0.4677342	-6.9185616	0.9000294
O	0.2518288	-4.1483265	0.8097719
H	3.1175958	-4.2588136	3.8681922
C	4.1734825	-4.0303300	3.7045113
O	2.2198616	-2.6144675	2.3161661
H	7.9159330	-3.1337403	3.0384767
C	6.8660094	-3.3801200	3.2206011
H	5.0277153	-5.3217664	-5.7628950
C	5.2648524	-4.6050984	-4.9713833
H	6.1135400	-2.0407709	-2.1512708
C	5.9019348	-2.7629161	-2.9435358
O	3.8054746	-1.4268172	-1.6952416
H	0.6084620	-8.8604526	1.8465337
C	0.5426419	-8.3132190	0.9017723
H	0.3184788	-6.3599624	-2.4714659
C	0.3801668	-6.9289484	-1.5406043
O	0.2126111	-4.1535633	-1.4591670
C	3.4679875	-2.3125427	-2.5529678
C	3.4301833	-2.3046875	2.0422111
C	0.2825664	-4.7390712	-0.3231766
C	4.5603389	-3.0294370	-3.2704840
C	6.5272600	-4.3698500	4.1543500
C	0.3837002	-6.2271365	-0.3218020
C	4.5092911	-3.0340837	2.7701023
C	6.6010500	-4.3337000	-4.6452400
C	0.5317700	-9.0095700	-0.3151600
O	1.1633307	-1.5081422	-0.2900035
H	2.1078789	0.0860366	-3.2391344
O	1.5081137	0.0576151	-2.4728909
Ce	0.1085068	-1.8847053	-2.1751187
H	2.0314827	0.0745226	2.6502305
O	1.4782525	0.0517810	1.8505287
Ce	0.0588057	-1.9047301	1.6050041
H	2.7536767	4.4269403	3.9453502
C	3.8240526	4.3108646	3.7590590

O	1.9943846	2.7758031	2.3291841
H	7.6247610	3.8200032	3.0073160
C	6.5588493	3.9518744	3.2141075
H	5.9851550	2.4445683	-2.2418724
C	5.6940529	3.1985436	-2.9771923
O	3.7299359	1.6760627	-1.7097206
H	4.5336954	5.8610133	-5.5916609
C	4.8501247	5.1146839	-4.8573230
H	-0.0537372	6.3855369	-2.4238592
C	-0.1128046	6.9485345	-1.4891769
O	-0.1443978	4.1690152	-1.4378513
H	-0.3219248	8.8560244	1.9119982
C	-0.2627526	8.3153648	0.9629942
H	2.8294167	4.4935555	-4.3263154
C	3.9015310	4.3629585	-4.1619774
O	2.0813563	2.8245965	-2.7593290
H	7.7084549	3.8055585	-3.4892062
C	6.6404856	3.9543215	-3.6723129
H	5.8974966	2.4829665	1.7455870
C	5.6087831	3.2076605	2.5106397
O	3.6563394	1.7173500	1.2043634
H	4.4622276	5.7721109	5.2234002
C	4.7766799	5.0521193	4.4622853
H	-0.2498795	6.3348202	1.8727792
C	-0.2241769	6.9190464	0.9496714
O	-0.1266062	4.1392493	0.8309538
H	-0.1138626	8.9077810	-2.4098144
C	-0.1470462	8.3447938	-1.4726098
C	3.2261528	2.5744131	2.0506069
C	3.3080811	2.5832244	-2.5100736
C	-0.1339014	4.7436652	-0.2975549
C	4.2408670	3.3855851	2.7853592
C	6.1398500	4.8683800	4.1890700
C	4.3255913	3.4031750	-3.2255126
C	6.2136200	4.9045600	-4.6105000
C	-0.1532117	6.2357600	-0.2773951
C	-0.2255200	9.0222000	-0.2474000
O	1.0413674	1.5774435	-0.2805388
Ce	-0.0965445	1.8867716	1.5971042
Ce	2.6821197	0.1144250	-0.2670349
Ce	-0.0426916	1.9099009	-2.2247207
H	7.4034741	-4.8441278	-5.1873958

H	7.3195758	-4.8931494	4.6989348
H	0.5880189	-10.1027628	-0.3122488
H	-6.9053154	-5.2291963	-5.5217315
H	-6.9876091	-5.2552180	4.8190916
H	6.9575589	5.4932998	-5.1569306
H	6.8853322	5.4490747	4.7415782
H	-0.2524636	-0.0488592	9.8339750
H	-0.2554095	10.1163655	-0.2354015
H	-7.4034155	4.6200443	4.8479004
H	-7.3123664	4.6650800	-5.5036239
H	-0.1241056	-0.1269648	-4.5729530
H	-1.0376853	-1.4487650	-4.7009465
H	-1.0100079	1.7123496	-4.7366905

D2[(OH)(H_2O)]$_2$

H	-3.0294157	-1.8912015	-0.0326509
O	-2.2414360	-1.3332945	-0.1380676
O	-2.9840076	-1.6307475	-2.9725198
H	-4.8619426	4.1836968	-2.3078663
C	-4.3240182	4.8145962	-3.0193022
O	-2.9358711	2.8506017	-1.6021818
H	-2.2880275	7.0098066	-5.5361341
C	-2.8497018	6.3889425	-4.8327397
O	0.2835240	1.4284680	-4.3115946
H	-6.0258325	6.0276562	-3.5886590
C	-4.9586269	5.8348647	-3.7303834
H	-1.1497652	5.1564745	-4.2529594
C	-2.2144957	5.3665855	-4.1279180
O	-1.0444463	3.2691302	-2.7876283
O	-4.2734417	0.1317111	-1.5716444
O	-0.8041033	-0.9292470	-4.4633491
C	-2.2649001	3.4885744	-2.4859680
C	-4.2173527	6.6123195	-4.6307657
C	-2.9536049	4.5817433	-3.2243425
O	-1.1198951	0.4523487	-1.7244310
H	-5.7573136	6.0532232	3.5115189
C	-4.7011198	5.8212038	3.6766121
H	-0.9332422	4.9872133	4.2592090
C	-1.9867482	5.2375282	4.1122848
O	-0.8226545	3.2117481	2.5810383
H	-6.3290310	-3.0791807	5.6251413

C	-6.3270225	-2.2880519	4.8698133
H	-6.3183732	0.5412044	2.1800710
C	-6.3462342	-0.2471506	2.9362162
O	-4.0686507	0.0894185	1.3713016
H	-0.7925421	-1.9597551	6.2050721
C	-0.4107640	-1.1073551	6.7722363
O	-0.3933735	-1.0405221	3.9841016
H	0.9723848	1.9905725	8.7021993
C	0.5861286	1.1239104	8.1578141
H	-8.3890546	0.1141287	3.5557084
C	-7.4886919	-0.4893484	3.7024768
H	-4.2676649	-2.6285742	4.2410193
C	-5.1821759	-2.0457695	4.1074695
O	-2.9458433	-1.4944395	2.5520183
H	-4.6376039	4.2031230	2.2179874
C	-4.0879023	4.7931976	2.9550064
O	-2.7928327	2.7780130	1.5401360
H	-2.0317816	6.8401399	5.5668148
C	-2.6033469	6.2642603	4.8333028
H	-0.7640123	-1.9732783	8.7235699
C	-0.3912784	-1.1065593	8.1699940
H	0.9342382	1.9792954	6.1838336
C	0.5684735	1.1258551	6.7599475
O	0.5360109	1.0296346	3.9708384
C	-2.0676882	3.4172538	2.3813110
C	-3.9806352	-0.7905151	2.3013258
C	0.0657694	0.0024657	4.5686682
C	-2.7285874	4.5015475	3.1699035
C	-7.4757225	-1.5115709	4.6620087
C	0.0711331	0.0086909	6.0642880
C	-5.1943138	-1.0286309	3.1364168
C	-3.9576277	6.5559235	4.6123977
C	0.1084220	0.0076590	8.8608954
O	-0.9656806	0.4338252	1.4191627
H	-0.7108030	3.5058423	-0.1260076
O	-0.5204337	2.5519490	-0.1436053
Ce	-2.4311424	1.0898541	-0.0981027
H	0.7992680	-5.3175445	-4.2860627
C	1.8683848	-5.5168406	-4.1801967
O	0.7276298	-3.3624467	-2.8121915
H	5.7006085	-6.1317155	-3.7333566
C	4.6277308	-5.9552408	-3.8525408

H	2.2433022	-7.1006171	5.1281272
C	2.7805619	-6.5209638	4.3720212
H	4.6899938	-4.4503335	1.6703659
C	4.1755682	-5.0430247	2.4303817
O	2.8354022	-2.9573566	1.1585014
H	-3.3841867	-8.2386081	-2.2819298
C	-3.1871485	-7.7135079	-1.3429489
H	-2.4640196	-5.8497425	2.0127168
C	-2.6774081	-6.3917056	1.0883831
O	-1.6250395	-3.8179242	0.9721451
H	1.0933355	-5.2251042	3.8980958
C	2.1353323	-5.4828660	3.6943168
O	0.9376862	-3.3664683	2.3337941
H	5.8621381	-6.3207683	2.8871182
C	4.8182122	-6.0815948	3.1099387
H	1.9200004	-7.1894987	-5.5539197
C	2.4968638	-6.5500933	-4.8796199
H	4.5550788	-4.2766006	-2.4654346
C	4.0011143	-4.9256288	-3.1473792
O	2.6887236	-2.9259332	-1.7570720
H	-3.4248290	-8.1848597	2.0447960
C	-3.2083901	-7.6833400	1.0973398
H	-2.4288458	-5.9019597	-2.2880051
C	-2.6593767	-6.4209291	-1.3546191
O	-1.7272063	-3.8122266	-1.2967767
C	1.9620248	-3.5905979	-2.5740788
C	2.1534320	-3.6133691	2.0175834
C	-1.8739341	-4.3686497	-0.1543359
C	2.6220425	-4.7046597	-3.3142912
C	4.1205777	-6.8158095	4.0806287
C	-2.4088998	-5.7601231	-0.1387148
C	2.8336198	-4.7403719	2.7246296
C	3.8733057	-6.7621055	-4.7156487
C	-3.4635168	-8.3349224	-0.1173330
O	0.3653423	-1.8675104	-0.2151085
H	1.7728830	-0.8117416	-3.2305925
O	1.2561779	-0.5862118	-2.4363565
Ce	-0.8594360	-1.7297832	-2.0840573
H	1.9797556	-0.8582366	2.6686814
O	1.4420603	-0.6276866	1.8914188
Ce	-0.6993910	-1.7432359	1.7214056
H	4.5835960	2.7650886	3.8225124

C	5.4854520	2.1785828	3.6315040
O	3.1260827	1.5761114	2.2996487
H	8.6514723	0.0276094	2.8688897
C	7.7611230	0.6270665	3.0797571
H	6.3215173	-0.5055426	-2.4794470
C	6.3818308	0.3395262	-3.1695691
O	4.0040257	-0.1504137	-1.7930436
H	6.4834413	3.3904384	-5.6083309
C	6.4480356	2.5377212	-4.9242284
H	2.5934561	5.8141311	-2.3752732
C	2.8605838	6.3212036	-1.4451308
O	1.6810301	3.8047642	-1.3668433
H	3.7785217	8.0346285	1.9418877
C	3.5183830	7.5491922	0.9968684
H	4.3874445	2.8777983	-4.2987122
C	5.2846943	2.2618759	-4.2034380
O	2.9986672	1.6237633	-2.7817832
H	8.4284004	-0.0160175	-3.7799854
C	7.5437601	0.6172852	-3.8932789
H	6.4865687	-0.4299063	1.6615223
C	6.5644897	0.3665177	2.4056402
O	4.1127639	-0.1007250	1.1341387
H	6.7362401	3.2424618	5.0414183
C	6.6832311	2.4368974	4.3031944
H	2.7543350	5.7294195	1.9216641
C	2.9514775	6.2723850	0.9943225
O	1.7859717	3.7490780	0.9007174
H	3.6192429	8.1197167	-2.3815501
C	3.4283941	7.5974533	-1.4396367
C	4.1343913	0.8531217	1.9838797
C	4.0001672	0.8625990	-2.5779247
C	1.9901834	4.3064425	-0.2343663
C	5.4235311	1.1404600	2.6838108
C	7.8176224	1.6591289	4.0284847
C	5.2527676	1.1619224	-3.3279362
C	7.5704505	1.7128529	-4.7678737
C	2.6190488	5.6602551	-0.2274859
C	3.7515887	8.2073764	-0.2191070
O	1.6195774	0.9555259	-0.2374744
Ce	0.8366182	1.7216049	1.6906765
Ce	2.4399578	-1.0903353	-0.2856062
Ce	0.7156729	1.7507485	-2.1392199

H	4.3645063	-7.5698849	-5.2674066
H	4.6257973	-7.6272000	4.6137321
H	-3.8804325	-9.3470722	-0.1086189
H	-8.3726568	-1.7026318	5.2597272
H	8.4818298	1.9280651	-5.3349300
H	8.7549810	1.8604668	4.5563715
H	0.1241115	0.0067308	9.9551207
H	4.1945792	9.2083423	-0.2156598
H	-4.4388755	7.3614674	5.1755812
H	-4.7184065	7.4120925	-5.1858444
H	-1.7812174	-0.8440559	-4.4887800
H	-0.4041382	0.0202446	-4.4768211
H	-0.3681196	2.1073169	-4.5753331
H	-3.4431131	-2.4899707	-3.0078716
H	-3.8174353	-0.5869990	-2.1658073
H	-4.8278143	-0.3544067	-0.9338006

Cu^{2+}–D1(OH)(H_2O)

H	-3.0664323	-1.8721890	-0.0019999
O	-2.2446404	-1.3657857	-0.1322062
H	-8.4169852	0.3216629	-3.5416313
C	-7.5705913	-0.3387489	-3.7470552
H	-4.5574622	-2.7138574	-4.4897349
C	-5.4035587	-2.0472765	-4.3035298
O	-2.8856786	-1.3511475	-3.2043062
H	-4.8827661	4.1773517	-2.3201600
C	-4.3346283	4.8006013	-3.0305849
O	-2.9346192	2.8264557	-1.6032078
H	-2.2866024	6.9938382	-5.5405737
C	-2.8525864	6.3723978	-4.8420430
O	0.4272895	2.0052917	-4.6360339
H	-6.0233402	6.0384862	-3.5780561
C	-4.9604320	5.8336697	-3.7301443
H	-1.1582610	5.1373722	-4.2830615
C	-2.2241472	5.3374762	-4.1498737
O	-1.0760575	3.1812744	-2.8414338
H	-6.7999780	-3.1760150	-5.5107867
C	-6.6566027	-2.3072523	-4.8631754
H	-6.1680447	0.7713021	-2.5102321
C	-6.3247759	-0.0816796	-3.1745139
O	-3.9010306	0.1165664	-1.8071537

O	-1.7651803	-0.8901873	-5.4188957
C	-3.9467497	-0.6842784	-2.7779029
C	-2.2932308	3.4522109	-2.5151080
C	-5.2372515	-0.9364329	-3.4516429
C	-4.2173527	6.6123195	-4.6307657
C	-2.9654754	4.5494821	-3.2448925
C	-7.7353644	-1.4552299	-4.5811087
O	-0.9917260	0.4207433	-1.7044443
H	-5.7426480	6.0345708	3.4955857
C	-4.6847098	5.8164328	3.6646583
H	-0.9102746	5.0212967	4.2552669
C	-1.9665563	5.2535724	4.0987330
O	-0.7756634	3.2178758	2.5394558
H	-6.3269493	-3.0702167	5.6396687
C	-6.3210202	-2.2807162	4.8833224
H	-6.2906894	0.5387136	2.1854800
C	-6.3148343	-0.2478019	2.9435314
O	-3.9650089	0.1348666	1.4455511
H	-0.7711767	-1.9557564	6.1953321
C	-0.3910279	-1.1009177	6.7596309
O	-0.3282594	-1.0391745	3.9660599
H	0.9793874	1.9884832	8.7114023
C	0.5968695	1.1241733	8.1619694
H	-8.3670242	0.1065597	3.5258252
C	-7.4691307	-0.4940363	3.6939217
H	-4.2566880	-2.6274574	4.2943305
C	-5.1635804	-2.0388043	4.1366798
O	-2.8990776	-1.5090385	2.5716877
H	-4.6114031	4.2176640	2.1919055
C	-4.0584126	4.7997681	2.9328057
O	-2.7279392	2.8135766	1.4761317
H	-2.0305812	6.8399316	5.5674731
C	-2.5987545	6.2690117	4.8282795
H	-0.7691080	-1.9697076	8.7034142
C	-0.3871770	-1.1025922	8.1580774
H	0.9728233	1.9904700	6.2020004
C	0.5959773	1.1318146	6.7630696
O	0.5888422	1.0245003	3.9456696
C	-2.0246575	3.4473696	2.3469742
C	-3.9340504	-0.7782364	2.3500560
C	0.1174792	0.0079333	4.5703189
C	-2.6935642	4.5140022	3.1436715

C	-7.4757225	-1.5115709	4.6620087
C	0.1034548	0.0163098	6.0554206
C	-5.1561634	-1.0221149	3.1601283
C	-3.9576277	6.5559235	4.6123977
C	0.1084220	0.0076590	8.8608954
O	-0.9240105	0.4166931	1.3980905
H	-0.6836713	3.4957973	-0.0852830
O	-0.4719282	2.5489044	-0.1720271
Ce	-2.4310246	1.0818992	-0.0925702
H	0.7719553	-5.3326449	-4.4321189
C	1.8430779	-5.4967250	-4.2921707
O	0.6233486	-3.2755004	-3.0800058
H	5.6662124	-6.0375921	-3.7321300
C	4.5949270	-5.8842017	-3.8875144
H	2.2425604	-7.1019783	5.1259431
C	2.7775000	-6.5261238	4.3664992
H	4.7033277	-4.4846971	1.6483796
C	4.1819917	-5.0662644	2.4117373
O	2.8322991	-3.0313539	1.0804541
H	-3.3887234	-8.2089635	-2.2804665
C	-3.1839726	-7.6986107	-1.3357739
H	-2.4424656	-5.8920890	2.0508330
C	-2.6583849	-6.4136099	1.1154901
O	-1.5625710	-3.8293214	1.0071208
H	1.0870601	-5.2469302	3.8783334
C	2.1309368	-5.4989798	3.6779349
O	0.9596915	-3.3608150	2.3027445
H	5.8690078	-6.3320331	2.8931555
C	4.8238633	-6.0925478	3.1057055
H	1.9326170	-7.2493610	-5.5543468
C	2.4957770	-6.5653630	-4.9139149
H	4.4976057	-4.1168662	-2.6256399
C	3.9480331	-4.8121254	-3.2648400
O	2.5369824	-2.6748804	-2.0565645
H	-3.4222973	-8.2105986	2.0468550
C	-3.2013224	-7.6994682	1.1063089
H	-2.4143126	-5.8901315	-2.2643284
C	-2.6443504	-6.4121237	-1.3328996
O	-1.6556165	-3.8224699	-1.2508807
C	1.8737083	-3.4611530	-2.8232171
C	2.1612166	-3.6624925	1.9881149
C	-1.8297664	-4.3979634	-0.1056509

C	2.5661014	-4.6099063	-3.4653849
C	4.1205777	-6.8158095	4.0806287
C	-2.3837615	-5.7675461	-0.1063793
C	2.8351153	-4.7665649	2.7006783
C	3.8733057	-6.7621055	-4.7156487
C	-3.4635168	-8.3349224	-0.1173330
O	0.4239753	-1.8281340	-0.2862375
H	1.5549328	-0.7481213	-3.7911643
O	0.6935346	-0.4532971	-3.4347898
Ce	-0.8408554	-1.7910501	-1.9580929
H	1.9814297	-0.8438832	2.6962744
O	1.4934225	-0.6487744	1.8755969
Ce	-0.6941880	-1.7549826	1.7776872
H	4.5909894	2.7759608	3.8153785
C	5.4906568	2.1858697	3.6254691
O	3.1392575	1.5677102	2.2486347
H	8.6638307	0.0297281	2.8751588
C	7.7714617	0.6269408	3.0794902
H	6.3111418	-0.5648806	-2.5414487
C	6.3592206	0.2906904	-3.2194965
O	3.9411904	-0.2523669	-1.8839843
H	6.4562700	3.3633928	-5.6274469
C	6.4242173	2.5030677	-4.9536921
H	2.6316200	5.8162326	-2.3970914
C	2.8850271	6.3133398	-1.4578407
O	1.7586159	3.7548836	-1.3881713
H	3.7733300	8.0216991	1.9415946
C	3.5218426	7.5386821	0.9938036
H	4.3353652	2.7836868	-4.4154794
C	5.2408915	2.1872332	-4.2802162
O	2.8876662	1.4123353	-2.9661021
H	8.4322199	-0.0034678	-3.7601667
C	7.5371673	0.6081674	-3.9011861
H	6.5120965	-0.4297752	1.6565377
C	6.5805816	0.3671831	2.4003125
O	4.1481923	-0.1117077	1.1265658
H	6.7379542	3.2426975	5.0420757
C	6.6841566	2.4400534	4.3019606
H	2.7822602	5.7123138	1.9147951
C	2.9711848	6.2554059	0.9858784
O	1.8375357	3.6990983	0.8687920
H	3.6224169	8.1242904	-2.3822527

C	3.4363949	7.5965440	-1.4432737
C	4.1694273	0.8593303	1.9763960
C	3.9460094	0.7324560	-2.7075685
C	2.0533043	4.2856191	-0.2481231
C	5.4380790	1.1453928	2.6762116
C	7.8176224	1.6591289	4.0284847
C	5.2050906	1.0787269	-3.4090519
C	7.5704505	1.7128529	-4.7678737
C	2.6493954	5.6404515	-0.2415771
C	3.7515887	8.2073764	-0.2191070
O	1.6390849	0.8306983	-0.3763236
Ce	0.8589071	1.7099629	1.6916294
Ce	2.5871752	-1.1520928	-0.2036166
Ce	0.7444626	1.8147626	-2.1126875
H	4.3829285	-7.5973706	-5.2033977
H	4.6257297	-7.6194548	4.6242939
H	-3.8898378	-9.3423164	-0.1203399
H	-8.7155587	-1.6628512	-5.0196237
H	-8.3796860	-1.7032755	5.2466323
H	8.4933826	1.9591081	-5.3002563
H	8.7514692	1.8595598	4.5618534
H	0.1124884	0.0042611	9.9542868
H	4.1834388	9.2121671	-0.2104104
H	-4.4473164	7.3502349	5.1818828
H	-4.7116107	7.4231437	-5.1738672
H	-2.3630996	-1.1606164	-4.6136996
H	-1.5135107	-1.6844352	-5.9251747
H	-0.2742194	2.6960027	-4.5892833
Cu	-0.4061810	0.3776645	-4.8458380

Cu^{2+}–D2[(OH)(H_2O)]$_2$

H	-3.0360150	-1.8904870	0.0205231
O	-2.2828871	-1.3164636	-0.2018890
O	-3.3762852	-1.7701544	-3.0324162
H	-4.8536732	4.1512097	-2.3398099
C	-4.3123863	4.7864624	-3.0447989
O	-2.9004290	2.8015064	-1.6518801
H	-2.2940624	7.0147562	-5.5479415
C	-2.8510694	6.3846869	-4.8500587
O	0.4699116	1.7938072	-4.5690589
H	-6.0139268	6.0124584	-3.5757703

C	-4.9496494	5.8197512	-3.7333576
H	-1.1444221	5.1554086	-4.3112567
C	-2.2099594	5.3503989	-4.1686239
O	-1.0286158	3.2140211	-2.8511854
O	-4.4900939	0.2767850	-1.4207148
O	-1.2667988	-1.4620885	-4.5589044
C	-2.2562695	3.4568915	-2.5458034
C	-4.2173527	6.6123195	-4.6307657
C	-2.9411280	4.5498928	-3.2660596
O	-1.1027874	0.4203177	-1.8308111
H	-5.7535039	6.0530394	3.5039479
C	-4.6977844	5.8219812	3.6694738
H	-0.9310478	4.9869967	4.2556731
C	-1.9847547	5.2318236	4.1016180
O	-0.8196669	3.1856427	2.5550574
H	-6.3207489	-3.0766503	5.6210388
C	-6.3229446	-2.2875756	4.8644073
H	-6.3268421	0.5447022	2.1780979
C	-6.3433883	-0.2494396	2.9286173
O	-4.0424402	0.1013424	1.3666817
H	-0.8003389	-1.9840116	6.2247591
C	-0.4148911	-1.1226467	6.7756783
O	-0.3827512	-1.0650858	3.9615690
H	0.9734510	1.9889358	8.6822890
C	0.5860900	1.1186850	8.1458100
H	-8.3821010	0.1152233	3.5498422
C	-7.4843617	-0.4908227	3.6976282
H	-4.2711369	-2.6424293	4.2384211
C	-5.1789374	-2.0508290	4.0983464
O	-2.9377334	-1.5130390	2.5015251
H	-4.6488407	4.2188778	2.2024387
C	-4.0873284	4.7968559	2.9402002
O	-2.7701186	2.7871912	1.4879850
H	-2.0261496	6.8242650	5.5627447
C	-2.6014983	6.2557036	4.8273293
H	-0.7673797	-1.9726103	8.7318763
C	-0.3936983	-1.1102338	8.1733527
H	0.9363210	1.9670414	6.1752866
C	0.5681811	1.1126853	6.7482964
O	0.5435350	0.9934288	3.9461094
C	-2.0656680	3.4248706	2.3612080
C	-3.9823295	-0.7952887	2.2903899

C	0.0704631	-0.0243716	4.5714181
C	-2.7246650	4.4972886	3.1505863
C	-7.4757225	-1.5115709	4.6620087
C	0.0693955	-0.0105417	6.0554635
C	-5.1865460	-1.0322171	3.1234538
C	-3.9576277	6.5559235	4.6123977
C	0.1084220	0.0076590	8.8608954
O	-0.9527542	0.4138856	1.3862845
H	-0.7131224	3.5003832	-0.0858871
O	-0.5013809	2.5516829	-0.1548427
Ce	-2.4347383	1.1091525	-0.0980769
H	0.7863177	-5.3168165	-4.3831753
C	1.8566204	-5.4941060	-4.2529204
O	0.6647122	-3.3063235	-2.9547448
H	5.6787630	-6.0842944	-3.7230248
C	4.6087355	-5.9151016	-3.8695255
H	2.2438405	-7.1025210	5.1290900
C	2.7759595	-6.5280613	4.3664901
H	4.6815141	-4.4830745	1.6402380
C	4.1631064	-5.0644383	2.4059230
O	2.8052020	-3.0139547	1.0886921
H	-3.3894537	-8.2577433	-2.2828156
C	-3.1971493	-7.7212171	-1.3502366
H	-2.4905897	-5.8274992	1.9967428
C	-2.6971675	-6.3735364	1.0734138
O	-1.6308673	-3.7874280	0.9142294
H	1.0760015	-5.2611285	3.8826564
C	2.1207685	-5.5054428	3.6766022
O	0.9201279	-3.3827884	2.2783301
H	5.8586205	-6.3192902	2.8820961
C	4.8128501	-6.0872540	3.1001308
H	1.9274603	-7.2147005	-5.5606728
C	2.4971263	-6.5513928	-4.9045325
H	4.5305059	-4.1800669	-2.5620646
C	3.9743150	-4.8539208	-3.2179192
O	2.6104434	-2.7257768	-1.9785369
H	-3.4238533	-8.1652943	2.0436932
C	-3.2138647	-7.6695067	1.0925740
H	-2.4604536	-5.9207634	-2.3184829
C	-2.6850001	-6.4240179	-1.3751760
O	-1.7926821	-3.7915757	-1.3410808
C	1.9209314	-3.4991145	-2.7340230

C	2.1338223	-3.6648859	1.9797624
C	-1.9163934	-4.3676919	-0.1874862
C	2.5933678	-4.6370734	-3.4079779
C	4.1205777	-6.8158095	4.0806287
C	-2.4380694	-5.7482776	-0.1627581
C	2.8147164	-4.7679287	2.6949164
C	3.8733057	-6.7621055	-4.7156487
C	-3.4635168	-8.3349224	-0.1173330
O	0.4016986	-1.8249034	-0.3035527
H	1.8703611	-0.7917908	-3.3964077
O	1.0136621	-0.4559632	-3.0639413
Ce	-0.8527479	-1.7838152	-2.0062128
H	1.9521228	-0.8694869	2.6972783
O	1.4601358	-0.6567847	1.8834233
Ce	-0.7096195	-1.7530298	1.7479130
H	4.5844689	2.7633714	3.8351398
C	5.4839287	2.1752459	3.6386845
O	3.1216662	1.5501202	2.2815032
H	8.6579778	0.0295319	2.8706191
C	7.7657891	0.6251019	3.0806374
H	6.2730537	-0.5179172	-2.5148561
C	6.3392507	0.3353343	-3.1944606
O	3.8663690	-0.1843389	-1.9174825
H	6.4982485	3.3949189	-5.6196790
C	6.4497169	2.5413494	-4.9384188
H	2.5877306	5.7927815	-2.3398226
C	2.8556432	6.3067445	-1.4142021
O	1.6848920	3.8037978	-1.3094017
H	3.7917032	8.0690923	1.9444313
C	3.5259593	7.5679245	1.0101397
H	4.3851302	2.8986712	-4.3581687
C	5.2715493	2.2713785	-4.2388454
O	2.9552311	1.6641941	-2.8192620
H	8.3937323	-0.0250849	-3.7649724
C	7.5160344	0.6137310	-3.8945189
H	6.5026845	-0.4383473	1.6664663
C	6.5712489	0.3603479	2.4085121
O	4.1275566	-0.1106880	1.1305205
H	6.7352159	3.2392674	5.0453229
C	6.6813480	2.4348911	4.3070689
H	2.7721405	5.7680885	1.9729324
C	2.9608111	6.2918994	1.0329123

O	1.7958659	3.7297854	0.9451188
H	3.6071760	8.0905716	-2.3794055
C	3.4217900	7.5813949	-1.4301937
C	4.1538495	0.8495377	1.9935804
C	3.9474150	0.8705084	-2.6524090
C	2.0058195	4.3209672	-0.1712152
C	5.4266442	1.1345660	2.6888594
C	7.8176224	1.6591289	4.0284847
C	5.2118628	1.1648865	-3.3653581
C	7.5704505	1.7128529	-4.7678737
C	2.6221342	5.6623232	-0.1815374
C	3.7515887	8.2073764	-0.2191070
O	1.6040047	0.8682497	-0.3195632
Ce	0.8429706	1.7147451	1.7270860
Ce	2.5527174	-1.1270127	-0.2107895
Ce	0.7530609	1.8602078	-2.0775445
H	4.3730102	-7.5896218	-5.2265986
H	4.6317463	-7.6149763	4.6247184
H	-3.8689347	-9.3507433	-0.0984956
H	-8.3699581	-1.6994436	5.2628812
H	8.4920176	1.9244215	-5.3173603
H	8.7544378	1.8640309	4.5545848
H	0.1253649	0.0135880	9.9541493
H	4.1941869	9.2076619	-0.2318892
H	-4.4373961	7.3579589	5.1797557
H	-4.7208127	7.4223523	-5.1664998
H	-2.9258078	-1.6329902	-3.9106645
H	-0.8681492	-2.1689568	-5.1021971
H	-0.2540257	2.4488555	-4.6770678
Cu	-0.2322612	0.0906313	-4.5327997
H	-3.7373187	-2.6764454	-3.0444720
H	-4.2385664	-0.4336896	-2.0614830
H	-5.0273176	-0.1505634	-0.7258573

Cu^{2+}–D1(OH)$_2$

H	-3.0639410	-1.8323721	-0.0374401
O	-2.2248655	-1.3456660	-0.1192766
H	-8.5343878	0.2844834	-3.5642513
C	-7.6643107	-0.3626073	-3.7047674
H	-4.5705779	-2.6865298	-4.2214183
C	-5.4457422	-2.0447839	-4.0959311

O	-3.0871914	-1.4138897	-2.8116485
H	-4.8394731	4.1733793	-2.3127596
C	-4.3054016	4.8048609	-3.0267043
O	-2.8832916	2.8688785	-1.5920013
H	-2.2961331	7.0148675	-5.5512450
C	-2.8510187	6.3895681	-4.8469799
O	0.4706785	1.7959338	-4.5511176
H	-6.0137201	6.0165014	-3.5735522
C	-4.9479432	5.8268780	-3.7272802
H	-1.1410194	5.1669259	-4.2929638
C	-2.2065223	5.3643373	-4.1542230
O	-1.0276318	3.2332652	-2.8391333
H	-6.7093596	-3.1499145	-5.4617957
C	-6.6349575	-2.2993404	-4.7790489
H	-6.3853306	0.7310677	-2.3205309
C	-6.4784327	-0.1085285	-3.0136447
O	-4.0767656	0.2187672	-1.5937044
O	-1.0275925	-1.5813682	-4.5354694
C	-4.1009258	-0.6894931	-2.4891532
C	-2.2453386	3.4860621	-2.5081691
C	-5.3687297	-0.9498192	-3.2137676
C	-4.2173527	6.6123195	-4.6307657
C	-2.9348580	4.5731854	-3.2446736
C	-7.7353644	-1.4552299	-4.5811087
O	-1.0282223	0.4514413	-1.7676315
H	-5.7500773	6.0361427	3.5069465
C	-4.6920572	5.8148260	3.6726006
H	-0.9186401	5.0107916	4.2597567
C	-1.9740710	5.2493740	4.1075635
O	-0.7982603	3.2015466	2.5784735
H	-6.3436149	-3.0793516	5.6441709
C	-6.3276611	-2.2872380	4.8903546
H	-6.2553100	0.5407009	2.2039747
C	-6.2937105	-0.2475674	2.9595585
O	-3.9335305	0.1492057	1.5026768
H	-0.7828742	-1.9784614	6.2164258
C	-0.4009877	-1.1209602	6.7756295
O	-0.3433710	-1.0711845	3.9769887
H	0.9769814	1.9879156	8.6939988
C	0.5927715	1.1187255	8.1530874
H	-8.3497718	0.1146315	3.5234855
C	-7.4559902	-0.4907671	3.6980754

H	-4.2591634	-2.6414052	4.3131985
C	-5.1633338	-2.0489325	4.1534760
O	-2.8927855	-1.5406510	2.5894345
H	-4.6214657	4.2051004	2.2097116
C	-4.0706892	4.7939022	2.9469904
O	-2.7460093	2.8136344	1.4924742
H	-2.0309462	6.8466719	5.5648289
C	-2.6000240	6.2702022	4.8302809
H	-0.7694860	-1.9710854	8.7300969
C	-0.3904222	-1.1097852	8.1732062
H	0.9562760	1.9639075	6.1806316
C	0.5844387	1.1123999	6.7554849
O	0.5721390	0.9981630	3.9627692
C	-2.0424784	3.4348164	2.3670610
C	-3.9102606	-0.7853868	2.3791621
C	0.0995616	-0.0265232	4.5744183
C	-2.7076478	4.5085361	3.1598182
C	-7.4757225	-1.5115709	4.6620087
C	0.0897194	-0.0096531	6.0628125
C	-5.1428530	-1.0287183	3.1826645
C	-3.9576277	6.5559235	4.6123977
C	0.1084220	0.0076590	8.8608954
O	-0.9242540	0.4170472	1.4171001
H	-0.7223648	3.4998828	-0.0566701
O	-0.4861524	2.5584507	-0.1317092
Ce	-2.4294995	1.0763681	-0.0955459
H	0.8047441	-5.3071442	-4.2738670
C	1.8761274	-5.4982567	-4.1775504
O	0.7248471	-3.3676041	-2.7968523
H	5.7138361	-6.0964553	-3.7810754
C	4.6392260	-5.9247772	-3.8887763
H	2.2424106	-7.0953709	5.1272814
C	2.7787551	-6.5230918	4.3657378
H	4.6998686	-4.4884031	1.6410787
C	4.1813401	-5.0690343	2.4072080
O	2.8370692	-3.0167234	1.0918410
H	-3.4092592	-8.2134866	-2.2811552
C	-3.1983078	-7.7007795	-1.3389631
H	-2.4336566	-5.8833194	2.0360204
C	-2.6568317	-6.4104177	1.1055121
O	-1.5633029	-3.8352164	0.9885415
H	1.0900494	-5.2406787	3.8721485

C	2.1333962	-5.4964046	3.6737011
O	0.9539902	-3.3777742	2.2943506
H	5.8674386	-6.3369793	2.8887471
C	4.8228007	-6.0956881	3.1029734
H	1.9025231	-7.2060780	-5.5044325
C	2.4933108	-6.5505106	-4.8590234
H	4.6020171	-4.1982380	-2.5636199
C	4.0275440	-4.8690136	-3.2070589
O	2.7116900	-2.7246917	-1.9474120
H	-3.4062363	-8.2097484	2.0463589
C	-3.1947144	-7.6985953	1.1033516
H	-2.4466543	-5.8851181	-2.2738180
C	-2.6652165	-6.4113450	-1.3419539
O	-1.7055779	-3.8206867	-1.2704640
C	1.9839644	-3.5205750	-2.6480148
C	2.1606625	-3.6562600	1.9810820
C	-1.8481527	-4.3909585	-0.1266063
C	2.6432284	-4.6536962	-3.3503523
C	4.1205777	-6.8158095	4.0806287
C	-2.3972013	-5.7670905	-0.1189125
C	2.8368485	-4.7666810	2.6961138
C	3.8733057	-6.7621055	-4.7156487
C	-3.4635168	-8.3349224	-0.1173330
O	0.4263693	-1.8032456	-0.2870295
H	1.8700215	-0.8460348	-3.3942758
O	1.0416290	-0.4761128	-3.0286222
Ce	-0.8741322	-1.8015474	-2.0431694
H	1.9776628	-0.8678437	2.7103870
O	1.4862193	-0.6546552	1.8972228
Ce	-0.6901713	-1.7569405	1.7364596
H	4.5888008	2.7676627	3.8090869
C	5.4906349	2.1794978	3.6241135
O	3.1401709	1.5551521	2.2754756
H	8.6636137	0.0231346	2.8846804
C	7.7710263	0.6214666	3.0862032
H	6.2754695	-0.4829030	-2.4794586
C	6.3433875	0.3577292	-3.1745002
O	3.8818832	-0.1564458	-1.8986909
H	6.4924969	3.3732970	-5.6533550
C	6.4468050	2.5316647	-4.9567607
H	2.5989972	5.7791671	-2.3302035
C	2.8665047	6.3004878	-1.4085287

O	1.7030843	3.8028454	-1.2985660
H	3.7958085	8.0760417	1.9444513
C	3.5319574	7.5709058	1.0114637
H	4.3763940	2.8843931	-4.3840573
C	5.2685343	2.2671489	-4.2547383
O	2.9616188	1.6829991	-2.8205012
H	8.4021418	-0.0019978	-3.7333884
C	7.5210136	0.6296706	-3.8761738
H	6.5075086	-0.4435477	1.6693018
C	6.5792028	0.3572030	2.4088742
O	4.1478872	-0.1120668	1.1244737
H	6.7374573	3.2480287	5.0331595
C	6.6845428	2.4402003	4.2982890
H	2.7870298	5.7651446	1.9753882
C	2.9738401	6.2915214	1.0364026
O	1.8218533	3.7348932	0.9607865
H	3.6073973	8.0871835	-2.3789586
C	3.4255036	7.5785260	-1.4284683
C	4.1626572	0.8479970	1.9808733
C	3.9468024	0.8887587	-2.6421390
C	2.0217347	4.3123494	-0.1614673
C	5.4381094	1.1354084	2.6810828
C	7.8176224	1.6591289	4.0284847
C	5.2143836	1.1775677	-3.3629836
C	7.5704505	1.7128529	-4.7678737
C	2.6378108	5.6600106	-0.1753783
C	3.7515887	8.2073764	-0.2191070
O	1.6278273	0.8928115	-0.2942481
Ce	0.8444307	1.7055803	1.7248344
Ce	2.5321674	-1.1218128	-0.2143791
Ce	0.7422076	1.8439230	-2.0587057
H	4.3557575	-7.5855221	-5.2502762
H	4.6256707	-7.6184354	4.6263340
H	-3.8853603	-9.3446324	-0.1153360
H	-8.6678289	-1.6519114	-5.1190702
H	-8.3859202	-1.7008296	5.2382906
H	8.4926569	1.9200877	-5.3186615
H	8.7523273	1.8638020	4.5593194
H	0.1172844	0.0136175	9.9547015
H	4.1877724	9.2108731	-0.2343081
H	-4.4447155	7.3554058	5.1779190
H	-4.7241105	7.4144257	-5.1760085

H	-2.0002898	-1.4563893	-4.5971709
H	-0.2747440	2.4334013	-4.6110846
Cu	-0.2080506	0.0763890	-4.5068293

Cu^{2+}–D2(OH)$_3$(H_2O)

H	-3.0822176	-1.8665442	-0.0321286
O	-2.2641985	-1.3511047	-0.1426686
O	-3.0496389	-1.5972267	-2.8384945
H	-4.8467879	4.1710444	-2.3189271
C	-4.3110654	4.8044528	-3.0295905
O	-2.8925574	2.8684486	-1.5958666
H	-2.2951864	7.0178892	-5.5477615
C	-2.8505962	6.3911997	-4.8451677
O	0.4769528	1.7626917	-4.5445738
H	-6.0176596	6.0166189	-3.5806848
C	-4.9514632	5.8270382	-3.7305746
H	-1.1426354	5.1658665	-4.2888669
C	-2.2080766	5.3653729	-4.1525411
O	-1.0334906	3.2388131	-2.8361419
O	-4.2842703	0.2288466	-1.5452672
O	-0.8999020	-1.6386418	-4.6697338
C	-2.2519839	3.4888028	-2.5098699
C	-4.2173527	6.6123195	-4.6307657
C	-2.9399796	4.5744529	-3.2456767
O	-1.0784807	0.4759486	-1.7575604
H	-5.7491356	6.0444831	3.5014481
C	-4.6931748	5.8168014	3.6717789
H	-0.9269855	4.9908977	4.2729846
C	-1.9805270	5.2353469	4.1166598
O	-0.8082294	3.1856326	2.5868775
H	-6.3289701	-3.0848355	5.6174407
C	-6.3242358	-2.2871438	4.8694561
H	-6.2984203	0.5634534	2.2050588
C	-6.3247012	-0.2332587	2.9525169
O	-4.0156661	0.1250472	1.4102917
H	-0.7788545	-1.9894030	6.2213789
C	-0.3988928	-1.1283444	6.7766045
O	-0.3486342	-1.0832633	3.9709863
H	0.9721963	1.9894485	8.6836217
C	0.5895071	1.1168301	8.1470576
H	-8.3717167	0.1231898	3.5536124

C	-7.4744089	-0.4826093	3.7072302
H	-4.2628192	-2.6300527	4.2600441
C	-5.1716197	-2.0402004	4.1184489
O	-2.9127657	-1.4818419	2.5647035
H	-4.6287698	4.2035033	2.2130587
C	-4.0760578	4.7898099	2.9508252
O	-2.7617500	2.7878699	1.5140865
H	-2.0317529	6.8359323	5.5701658
C	-2.6024405	6.2616913	4.8351245
H	-0.7642966	-1.9739821	8.7331679
C	-0.3875205	-1.1126321	8.1746600
H	0.9506393	1.9571046	6.1725479
C	0.5805952	1.1059129	6.7491065
O	0.5712374	0.9837686	3.9544377
C	-2.0523837	3.4170170	2.3807691
C	-3.9518652	-0.7699753	2.3307648
C	0.0968872	-0.0390211	4.5682932
C	-2.7152487	4.4962441	3.1684105
C	-7.4757225	-1.5115709	4.6620087
C	0.0882476	-0.0188579	6.0581883
C	-5.1700588	-1.0145446	3.1533317
C	-3.9576277	6.5559235	4.6123977
C	0.1084220	0.0076590	8.8608954
O	-0.9376786	0.4196965	1.4021677
H	-0.7123150	3.5180051	-0.0525459
O	-0.4949207	2.5719401	-0.1275262
Ce	-2.4311384	1.0963177	-0.1014221
H	0.7749342	-5.3478987	-4.3772257
C	1.8482535	-5.5145713	-4.2578271
O	0.6595441	-3.2974340	-3.0478560
H	5.6820669	-6.0505125	-3.7546073
C	4.6083624	-5.8956403	-3.8916831
H	2.2381894	-7.0937587	5.1207898
C	2.7763875	-6.5240924	4.3584702
H	4.6974719	-4.4958224	1.6306401
C	4.1771126	-5.0738892	2.3976260
O	2.8206686	-3.0326692	1.0587531
H	-3.3732099	-8.2078427	-2.2795051
C	-3.1668921	-7.7021973	-1.3324298
H	-2.4335609	-5.9043558	2.0602872
C	-2.6470432	-6.4263839	1.1245409
O	-1.6086947	-3.8259324	1.0314721

H	1.0840242	-5.2509006	3.8548102
C	2.1290705	-5.5025298	3.6594876
O	0.9426751	-3.3900953	2.2691692
H	5.8669306	-6.3322436	2.8894616
C	4.8205783	-6.0953811	3.1007302
H	1.9281555	-7.2586884	-5.5355647
C	2.4944304	-6.5756226	-4.8967087
H	4.5149729	-4.1392353	-2.6092893
C	3.9660849	-4.8331416	-3.2503551
O	2.5836187	-2.7300676	-2.0089268
H	-3.4371412	-8.2177905	2.0473049
C	-3.2031237	-7.7067771	1.1095473
H	-2.3678240	-5.8969886	-2.2485916
C	-2.6103094	-6.4228689	-1.3225068
O	-1.5544237	-3.8710677	-1.2286662
C	1.8967326	-3.4924014	-2.7812939
C	2.1490400	-3.6685810	1.9558686
C	-1.7971513	-4.4117642	-0.0919239
C	2.5836928	-4.6386347	-3.4350286
C	4.1205777	-6.8158095	4.0806287
C	-2.3552097	-5.7846305	-0.0934214
C	2.8304059	-4.7733329	2.6792689
C	3.8733057	-6.7621055	-4.7156487
C	-3.4635168	-8.3349224	-0.1173330
O	0.3877257	-1.7934977	-0.3030835
H	1.9155345	-0.8452377	-3.2297240
O	1.0523108	-0.4582378	-2.9743405
Ce	-0.9660153	-1.7926015	-2.0733890
H	1.9719904	-0.8832766	2.6887292
O	1.4749047	-0.6655605	1.8801523
Ce	-0.7017239	-1.7547607	1.7193692
H	4.5800555	2.7444688	3.7908822
C	5.4851969	2.1608025	3.6076257
O	3.1407385	1.5344835	2.2543490
H	8.6734251	0.0290229	2.8824642
C	7.7773091	0.6228924	3.0823262
H	6.2400195	-0.5303011	-2.5441857
C	6.3188759	0.3299180	-3.2137444
O	3.8351578	-0.1930871	-1.9635600
H	6.5143277	3.4138227	-5.6021402
C	6.4576039	2.5524281	-4.9308925
H	2.5908808	5.7827633	-2.3307203

C	2.8601360	6.3025811	-1.4086972
O	1.6899859	3.8061731	-1.2992420
H	3.7957043	8.0736873	1.9444356
C	3.5302192	7.5701664	1.0109832
H	4.3933819	2.9210874	-4.3471713
C	5.2749603	2.2853424	-4.2359652
O	2.9586282	1.7028840	-2.8117902
H	8.3723328	-0.0443346	-3.7814346
C	7.5015865	0.6051858	-3.9072560
H	6.5313964	-0.4502184	1.6558794
C	6.5901471	0.3507308	2.3967323
O	4.1445730	-0.1253545	1.0902695
H	6.7135519	3.2369268	5.0256510
C	6.6741433	2.4291533	4.2897006
H	2.7812852	5.7661135	1.9747477
C	2.9687011	6.2921074	1.0356388
O	1.8145581	3.7347473	0.9596794
H	3.6055004	8.0873339	-2.3789083
C	3.4227796	7.5792057	-1.4283060
C	4.1641912	0.8279979	1.9532205
C	3.9255856	0.8819922	-2.6652860
C	2.0125292	4.3137780	-0.1631860
C	5.4397819	1.1185904	2.6607399
C	7.8176224	1.6591289	4.0284847
C	5.2012307	1.1710053	-3.3762800
C	7.5704505	1.7128529	-4.7678737
C	2.6305059	5.6612418	-0.1760138
C	3.7515887	8.2073764	-0.2191070
O	1.6120090	0.8929502	-0.3003343
Ce	0.8460049	1.7008581	1.7181297
Ce	2.4867766	-1.1335141	-0.2421146
Ce	0.7193050	1.8503105	-2.0694628
H	4.3791243	-7.5920665	-5.2176261
H	4.6270418	-7.6140313	4.6311794
H	-3.9031712	-9.3369743	-0.1250914
H	-8.3771123	-1.7073383	5.2500070
H	8.4957348	1.9219537	-5.3124760
H	8.7481419	1.8686905	4.5641585
H	0.1176772	0.0170422	9.9545563
H	4.1909837	9.2094193	-0.2341227
H	-4.4413467	7.3599700	5.1742459
H	-4.7229243	7.4150917	-5.1763072

H	-3.1036505	-1.6210155	-3.8127795
H	-0.2777753	-2.3679601	-4.8722288
H	-0.2924894	2.3522759	-4.6905556
Cu	-0.0392604	-0.0183622	-4.5759056
H	-3.8621185	-0.5074128	-2.1464200
H	-4.9626669	-0.2128475	-1.0031676

References

[1] J. Yang, K. Li, C. Li, J. Gu, *Small* **2021**, *17*, 2101455.

[2] M. Lammert, M. T. Wharmby, S. Smolders, B. Bueken, A. Lieb, K. A. Lomachenko, D. De Vos, N. Stock, *Chem. Commun.* **2015**, *51*, 12578-12581.

[3] a) S. Rojas-Buzo, D. Salusso, T.-H. T. Le, M. A. Ortuño, K. A. Lomachenko, S. Bordiga, *J. Phys. Chem. Lett.* **2024**, *15*, 3962-3967; b) X. He, B. G. Looker, K. T. Dinh, A. W. Stubbs, T. Chen, R. J. Meyer, P. Serna, Y. Román-Leshkov, K. M. Lancaster, M. Dincă, *ACS Catal.* **2020**, *10*, 7820-7825.

[4] Y.-T. Xu, M.-Y. Xie, H. Zhong, Y. Cao, *ACS Catal.* **2022**, *12*, 8698-8706.

[5] C.-H. Shen, Y.-H. Chen, Y.-C. Wang, T.-E. Chang, Y.-L. Chen, C.-W. Kung, *Phys. Chem. Chem. Phys.* **2022**, *24*, 9855-9865.

[6] a) C.-H. Chuang, J.-H. Li, Y.-C. Chen, Y.-S. Wang, C.-W. Kung, *J. Phys. Chem. C* **2020**, *124*, 20854-20863; b) Y.-N. Chang, C.-H. Shen, C.-W. Huang, M.-D. Tsai, C.-W. Kung, *ACS Appl. Nano Mater.* **2023**, *6*, 3675-3684.

[7] Y. D. Song, W. H. Ho, Y. C. Chen, J. H. Li, Y. S. Wang, Y. J. Gu, C. H. Chuang, C. W. Kung, *Chem. Eur. J.* **2021**, *27*, 3560-3567.

[8] C. Jia, Y. Zhao, S. Song, Q. Sun, Q. Meyer, S. Liu, Y. Shen, C. Zhao, *Adv. Energy Mater.* **2023**, *13*, 2302007.

[9] Z. Wang, S. Liu, M. Wang, L. Zhang, Y. Jiang, T. Qian, J. Xiong, C. Yang, C. Yan, *ACS Catal.* **2023**, *13*, 9125-9135.

[10] D. Zhu, L. Zhang, R. E. Ruther, R. J. Hamers, *Nat. Mater.* **2013**, *12*, 836-841.

[11] V. Bernales, M. A. Ortuño, D. G. Truhlar, C. J. Cramer, L. Gagliardi, *ACS Cent. Sci.* **2018**, *4*, 5-19.

[12] a) J. P. Perdew, K. Burke, M. Ernzerhof, *Phys. Rev. Lett.* **1996**, *77*, 3865; b) S. Grimme, S. Ehrlich, L. Goerigk, *J. Comput. Chem.* **2011**, *32*, 1456-1465; c) G. Kresse, J. Furthmüller, *Comput. Mater. Sci.* **1996**, *6*, 15-50; d) G. Kresse, J. Hafner, *Phys. Rev. B* **1993**, *47*, 558.

[13] a) P. E. Blöchl, *Phys. Rev. B* **1994**, *50*, 17953; b) G. Kresse, D. Joubert, *Phys. Rev. B*

1999, *59*, 1758.

[14] Y. Zhu, J. Zheng, J. Ye, Y. Cui, K. Koh, L. Kovarik, D. M. Camaioni, J. L. Fulton, D. G. Truhlar, M. Neurock, *Nat. Commun.* **2020**, *11*, 5849.

[15] M. A. Ortuño, V. Bernales, L. Gagliardi, C. J. Cramer, *J. Phys. Chem. C* **2016**, *120*, 24697-24705.

[16] a) M. Dolg, U. Wedig, H. Stoll, H. Preuss, *J. Chem. Phys.* **1987**, *86*, 866-872; b) M. Dolg, H. Stoll, H. Preuss, *J. Chem. Phys.* **1989**, *90*, 1730-1734.

[17] S. Grimme, *Chem. Eur. J.* **2012**, *18*, 9955-9964.

[18] a) A. D. Becke, *J. Chem. Phys.* **1992**, *96*, 2155-2160; b) C. Lee, W. Yang, R. G. Parr, *Phys. Rev. B* **1988**, *37*, 785; c) S. H. Vosko, L. Wilk, M. Nusair, *Can. J. Phys.* **1980**, *58*, 1200-1211; d) F. Weigend, R. Ahlrichs, *Phys. Chem. Chem. Phys.* **2005**, *7*, 3297-3305.

[19] A. V. Marenich, C. J. Cramer, D. G. Truhlar, *J. Phys. Chem. B* **2009**, *113*, 6378-6396.

[20] a) M. J. Frisch, G. W. Trucks, H. B. Schlegel, G. E. Scuseria, M. A. Robb, J. R. Cheeseman, G. Scalmani, V. Barone, G. A. Petersson, H. Nakatsuji, *Gaussian Inc: Wallingford, CT, USA* **2016,** 2016; b) S. G. Balasubramani, G. P. Chen, S. Coriani, M. Diedenhofen, M. S. Frank, Y. J. Franzke, F. Furche, R. Grotjahn, M. E. Harding, C. Hättig, *J. Chem. Phys.* **2020**, *152*.

[21] T. A. Manz, N. G. Limas, *RSC Adv.* **2016**, *6*, 47771-47801.

[22] A. P. Thompson, H. M. Aktulga, R. Berger, D. S. Bolintineanu, W. M. Brown, P. S. Crozier, P. J. In't Veld, A. Kohlmeyer, S. G. Moore, T. D. Nguyen, *Comput. Phys. Commun.* **2022**, *271*, 108171.

[23] L. Liu, Z. Chen, J. Wang, D. Zhang, Y. Zhu, S. Ling, K.-W. Huang, Y. Belmabkhout, K. Adil, Y. Zhang, *Nat. Chem.* **2019**, *11*, 622-628.

[24] M. A. Addicoat, N. Vankova, I. F. Akter, T. Heine, *J. Chem. Theory Comput.* **2014**, *10*, 880-891.

[25] P. G. Boyd, S. M. Moosavi, M. Witman, B. Smit, *J. Phys. Chem. Lett.* **2017**, *8*, 357-363.

[26] C. Campañá, B. Mussard, T. K. Woo, *J. Chem. Theory Comput.* **2009**, *5*, 2866-2878.

[27] a) L. S. Dodda, J. Z. Vilseck, J. Tirado-Rives, W. L. Jorgensen, *J. Phys. Chem. B* **2017**, *121*, 3864-3870; b) L. S. Dodda, I. Cabeza de Vaca, J. Tirado-Rives, W. L. Jorgensen, *Nucleic Acids Res.* **2017**, *45*, W331-W336; c) W. L. Jorgensen, D. S. Maxwell, J. Tirado-Rives, *J. Am. Chem. Soc.* **1996**, *118*, 11225-11236; d) B. Doherty, X. Zhong, S. Gathiaka, B. Li, O. Acevedo, *J. Chem. Theory Comput.* **2017**, *13*, 6131-6145.

[28] J. L. Abascal, C. Vega, *J. Chem. Phys.* **2005**, *123*.

[29] H. J. Berendsen, J. v. Postma, W. F. Van Gunsteren, A. DiNola, J. R. Haak, *J. Chem. Phys.* **1984**, *81*, 3684-3690.

[30] T. Darden, D. York, L. Pedersen, *J. Chem. Phys.* **1993**, *98*, 10089-10092.

[31] J.-P. Ryckaert, G. Ciccotti, H. J. Berendsen, *J. Comput. Phys.* **1977**, *23*, 327-341.

[32] a) W. G. Hoover, *Phys. Rev. A* **1985**, *31*, 1695; b) S. Nosé, *J. Chem. Phys.* **1984**, *81*, 511-519.

[33] N. Michaud-Agrawal, E. J. Denning, T. B. Woolf, O. Beckstein, *J. Comput. Chem.* **2011**, *32*, 2319-2327.

[34] M. R. DeStefano, T. Islamoglu, S. J. Garibay, J. T. Hupp, O. K. Farha, *Chem. Mater.* **2017**, *29*, 1357-1361.

[35] Y. Wang, W. Zhou, R. Jia, Y. Yu, B. Zhang, *Angew. Chem., Int. Ed.* **2020**, *59*, 5350-5354.

[36] Z. Gong, W. Zhong, Z. He, Q. Liu, H. Chen, D. Zhou, N. Zhang, X. Kang, Y. Chen, *Appl. Catal. B: Environ.* **2022**, *305*, 121021.

[37] T. Ren, K. Ren, M. Wang, M. Liu, Z. Wang, H. Wang, X. Li, L. Wang, Y. Xu, *Chem. Eng. J.* **2021**, *426*, 130759.

[38] G.-F. Chen, Y. Yuan, H. Jiang, S.-Y. Ren, L.-X. Ding, L. Ma, T. Wu, J. Lu, H. Wang, *Nat. Energy* **2020**, *5*, 605-613.

[39] C. Li, S. Liu, Y. Xu, T. Ren, Y. Guo, Z. Wang, X. Li, L. Wang, H. Wang, *Nanoscale* **2022**, *14*, 12332-12338.

[40] Y. Xu, M. Wang, K. Ren, T. Ren, M. Liu, Z. Wang, X. Li, L. Wang, H. Wang, *J. Mater.*

Chem. A **2021**, *9*, 16411-16417.

[41] J. Yang, H. Qi, A. Li, X. Liu, X. Yang, S. Zhang, Q. Zhao, Q. Jiang, Y. Su, L. Zhang, *J. Am. Chem. Soc.* **2022**, *144*, 12062-12071.

[42] M. Jiang, Q. Zhu, X. Song, Y. Gu, P. Zhang, C. Li, J. Cui, J. Ma, Z. Tie, Z. Jin, *Environ. Sci. Technol.* **2022**, *56*, 10299-10307.

[43] R. Daiyan, T. Tran-Phu, P. Kumar, K. Iputera, Z. Tong, J. Leverett, M. H. A. Khan, A. A. Esmailpour, A. Jalili, M. Lim, *Energy Environ. Sci.* **2021**, *14*, 3588-3598.

[44] P. Li, R. Li, Y. Liu, M. Xie, Z. Jin, G. Yu, *J. Am. Chem. Soc.* **2023**, *145*, 6471-6479.

[45] Z. Song, Y. Liu, Y. Zhong, Q. Guo, J. Zeng, Z. Geng, *Adv. Mater.* **2022**, *34*, 2204306.

[46] X. Zhu, H. Huang, H. Zhang, Y. Zhang, P. Shi, K. Qu, S.-B. Cheng, A.-L. Wang, Q. Lu, *ACS Appl. Mater. Interfaces* **2022**, *14*, 32176-32182.

[47] W. J. Sun, H. Q. Ji, L. X. Li, H. Y. Zhang, Z. K. Wang, J. H. He, J. M. Lu, *Angew. Chem., Int. Ed.* **2021**, *60*, 22933-22939.
